# Synthesis and Evaluation of Chalcone-Quinoline Based Molecular Hybrids as Potential Anti-Malarial Agents

**DOI:** 10.3390/molecules26134093

**Published:** 2021-07-05

**Authors:** Bonani Vinindwa, Godwin Akpeko Dziwornu, Wayiza Masamba

**Affiliations:** 1 Department of Chemical and Physical Sciences, Faculty of Natural Sciences, Walter Sisulu University, Nelson Mandela Drive, Mthatha 5117, South Africa; athulevinindwa@gmail.com; 2 Department of Chemistry, University of Cape Town, Rondebosch 7700, South Africa; godwin.dziwornu@uct.ac.za

**Keywords:** chalcones, malaria, molecular hybrids, quinoline-sulfonamide, *P. falciparum*

## Abstract

Molecular hybridization is a drug discovery strategy that involves the rational design of new chemical entities by the fusion (usually via a covalent linker) of two or more drugs, both active compounds and/or pharmacophoric units recognized and derived from known bioactive molecules. The expected outcome of this chemical modification is to produce a new hybrid compound with improved affinity and efficacy compared to the parent drugs. Additionally, this strategy can result in compounds presenting modified selectivity profiles, different and/or dual modes of action, reduced undesired side effects and ultimately lead to new therapies. In this study, molecular hybridization was used to generate new molecular hybrids which were tested against the chloroquine sensitive (NF54) strain of *P. falciparum*. To prepare the new molecular hybrids, the quinoline nucleus, one of the privileged scaffolds, was coupled with various chalcone derivatives via an appropriate linker to produce a total of twenty-two molecular hybrids in 11%–96% yield. The synthesized compounds displayed good antiplasmodial activity with IC_50_ values ranging at 0.10–4.45 μM.

## 1. Introduction

Malaria is a life-threatening disease caused by the *Plasmodium* parasites that are transmitted to people through the bites of infected female *Anopheles* mosquitoes [1]. Pregnant women, children under the age of 5 years, and people with weakened immune system are most likely to succumb to the morbidity and transience of the disease [2,3]. In 2019 the estimated malaria cases were 229 million with an approximated death toll of 409,000 [4]. Worldwide, children aged under 5 years, the most vulnerable group, accounted for 67% of the total malaria deaths in 2019. The World Health Organization (WHO) Africa region accounted for 82% of all cases and about 10% in the WHO South-East Asia region. *P. falciparum* is the most prevalent malaria parasite in the WHO regions of Africa, South-East Asia, the Eastern Mediterranean and the Western Pacific, accounting for 99.7% of all malaria cases in Africa, 50% in South-East Asia, 69% in Eastern Mediterranean, and 65% in Western Pacific. On the other hand, *P. vivax* is predominant in the WHO region of the Americas, representing 75% of malaria cases [5].

For a long time, chloroquine (CQ) remained the antimalarial drug of choice due to its high efficacy, low cost, and tolerable adverse effects. However, large scale use of CQ soon led to the emergence of CQ-resistant parasite strains [2]. Many other alternative drugs followed, including artemisinin combined therapies (ACTs), which for some time became one of the most effective, especially against *P. falciparum*, allowing millions of patients to be cured [1,2,3,6,7,8]. However, recent evidence of resistance to artemisinin reported in South Asia and along the Cambodia–Thailand border threatens this strategy [9]. Indeed, as soon as a new drug is introduced, it is just a matter of time before resistance appears. Under these circumstances, there is need to be one step ahead of the malaria parasite by designing new strategies in the fight against this pathology. Hence, multi-target hybrid drugs are needed as an alternative or compliment to existing therapies.

Molecular hybridization (MH) is a strategy of rational design of new ligands or prototypes based on the recognition of pharmacophoric subunits in the molecular structure of two or more known bioactive derivatives which through the adequate fusion of these subunits, leads to the formation of new hybrid architectures that maintain pre-selected characteristics of the original templates [8,10,11]. Hybrid drugs are designed to counterbalance the known side effects associated with the other hybrid part or to augment its effect through action on another biological target or to interact with multiple targets as one single molecule, lowering the risk of drug-drug interactions and surmount drug resistance [11]. Over the last few years, molecular hybridization has emerged as a novel approach that involves a combination of pharmacophoric elements which are so far known for different enzyme inhibition, leading to active compounds which show a balanced inhibition of enzyme and offers the opportunity to treat different diseases with better tolerated drugs, thus enhancing patient compliance [11]. We therefore sought to apply this concept of molecular hybridization in the design and synthesis of new hybrid compounds based on new hybrid compounds based on the quinoline and chalcone privileged scaffolds connected by an aminoalkylsulphonamide linker.

**Figure 1:**
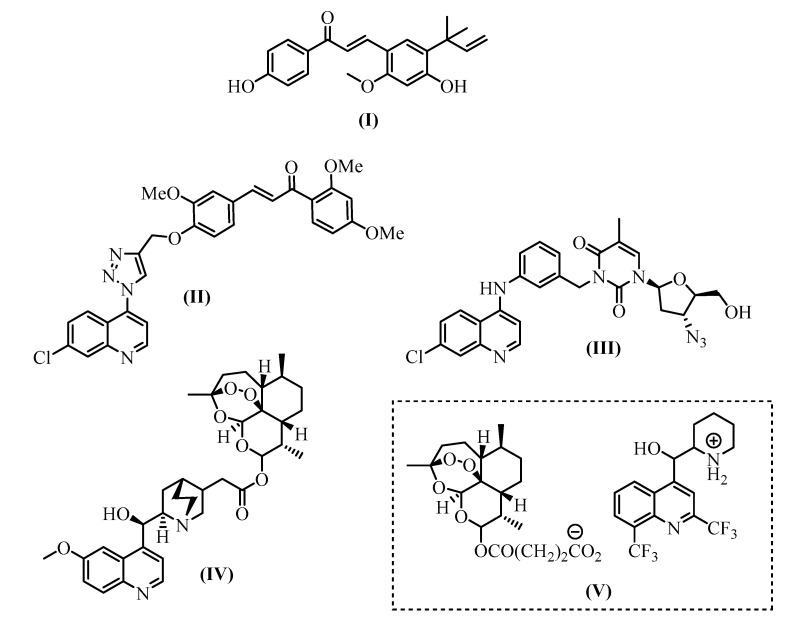
Structures of licochalcone (**I**) and molecular hybrids **II–V**.

Privileged structures are core structural units known for binding to proteins, and which can be coupled to other active molecules to generate potential new drugs. 1,3-Diarylprop-2-en-1-ones or benzalacetophenones commonly known as chalcones belong to an important class of natural products widely distributed in the plant kingdom. They display an array of biological activities [12] such as antifungal [13], antiparasitic [14], antitumor and antioxidant [15], immunomodulatory [16] antileishmania [17], antimitotic [18], anti-invasive [19,20], anti-inflammatory [21,22] and antimalarial [23,24]. Licochalcone (Figure 1**I**), for example, is a natural compound isolated from Chinese liquorice roots, which has shown potent antiplasmodial activity [25]. Due to this broad range of biological activities, the chalcone molecule is one of the templates extensively used in molecular hybridization. When combined with quinolines, chalcones may be expected to exhibit high antiplasmodial activity.

Of the several antimalarial compounds described in literature, quinoline derivatives are still the dominant class [7]. Furthermore, the diverse pharmacological properties of the quinoline scaffold and derivatives attracted worldwide attention in the last few decades because of their wide occurrence in natural products, and drugs [26]. Synthetic hybrid compounds have been designed to increase the efficacy of quinoline derivatives because the antimalarial hybrid drugs have a lower risk of drug–drug adverse interactions and greater treatment adherence than the combined drugs. Some of the quinoline derivatives shown in Figure 1 have reached the stage of clinical trials. They include chalcone–chloroquinoline (**II**) [8], AZT–CQ (**III**) [27], quinine–dihydroartemisinin (**IV**) [28] and artesunate–mefloquine MEFAS (**V**) [7].

**Figure 2:**
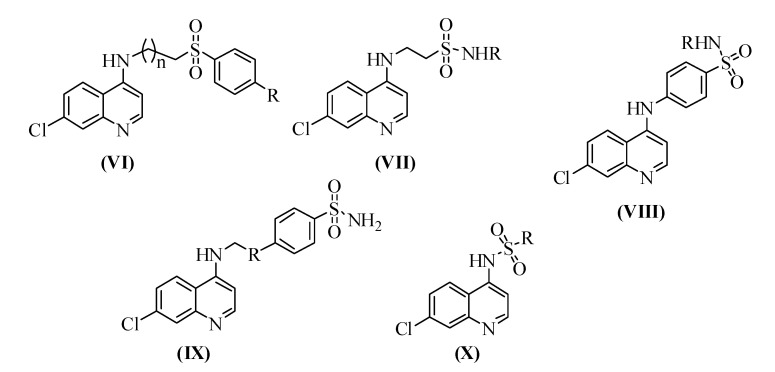
Representative quinoline–sulfonamide molecular hybrids **VI**–**X**.

Quinoline–sulfonamide hybrid compounds such as those in Figure 2, which contain various linker groups connecting arylsulfonamide moieties to the aminoquinoline molecule, have been reported as potential antimalarial drugs [7,29,30,31,32,33]. Though most of them were less active when compared to chloroquine, they showed good antimalarial activity, acting by inhibiting parasite growth without interfering with the integrity of the red blood cell membrane [29].

Based on the above properties and literature precedents, we sought in this study to synthesize molecular hybrids comprising of a quinoline–sulfonamide and the relevant chalcones as potential antiplasmodial agents.

## 2. Results

### 2.1. Chemistry

**Scheme 1:**
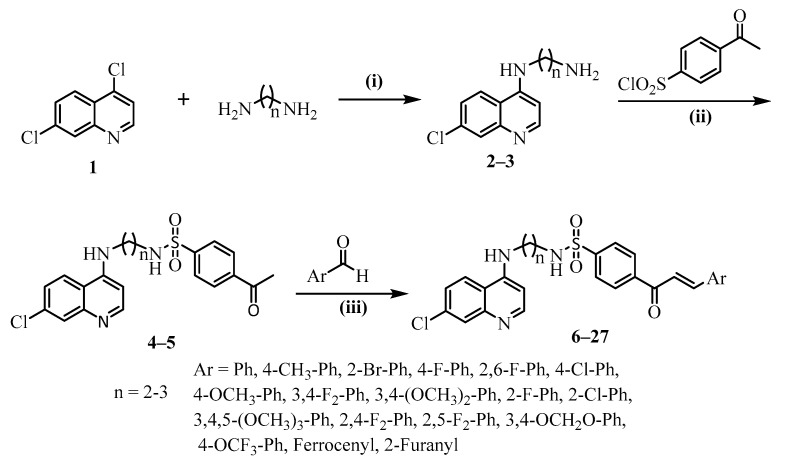
Synthesis of molecular hybrids **1–2**. Reagents and conditions: (i) appropriate diamine, reflux, 16 h; (ii) 4-acetylbenzenesulfonyl chloride, dioxane, NaOH, rt, 24 h; (iii) benzaldehyde derivative, MeOH, NaOH, reflux overnight.

The synthesis of molecular hybrids was carried out in three consecutive steps as outlined in Scheme 1. In a first step, the nucleophilic aromatic substitution reaction (S_N_Ar) between 4,7-dichloroquinoline and the relevant diamines (n = 2 and 3) afforded the corresponding N-(7-chloroquinol-4-yl) alkyldiamines **2** and **3** in excellent yields (89% and 95%, respectively) [7,34], which were condensed with 4-acetylbenzenesulfonyl chloride in the presence of NaOH to afford 4-acetyl-N-(2-[(7-chloroquinolin-4-yl)amino]alkyl)benzenesulfonamides **4** and **5** in moderate yields (29 and 41%, respectively). Finally, a base-catalyzed Claisen-Schmidt coupling of these intermediates with various aryl aldehydes led to the expected molecular hybrids in 11%–96% yield. Table 1 summarizes the overall results in these reactions. Except for compounds **11**, **12**, **14**, **18**, **23**, and **25**, good to excellent yields were obtained in the condensation step. Only trans isomers of the chalcone moiety were obtained as confirmed by the coupling constant between the two olefinin protons (~15.65 Hz in all cases).

### 2.2. In-Vitro Antiplasmodial Activity Evaluation

All the synthesized molecular hybrids (**6–27**) were evaluated for *in vitro* antiplasmodial activity against the drug-sensitive strain (NF54) of *P. falciparum* and for cytotoxicity against the Chinese Hamster Ovary (CHO) mammalian cell line using emetine as a reference drug. Aqueous solubility was determined at pH 6.5 (Table 2).

## 3. Discussion

All synthesized compounds presented good activity with IC_50_ values ranging from 0.10 to 4.45 μM against the NF54 sensitive strain of *P. falciparum*.

Compared to the unsubstituted molecular hybrid **6** (IC_50_ = 1.67μM), fluoro-substituted derivatives **9**, **15,** and **24** are all more active (with IC_50_ (NF54) values of 0.28, 0.86, and 0.57 μM, respectively), suggesting the importance of electronic effects brought about by the more electronegative fluorine atom. The same trend can be noted with other halogens, such as bromine (compound **8**; IC_50_ = 0.10 μM), chlorine (compound **11**, IC_50_ = 0.10 μM), as well as a methoxy group (compound **12**, IC_50_ = 0.11 μM). Compounds **8, 11,** and **12** were the most active, displaying IC_50_ values of 0.10, 0.10, and 0.11 μM, respectively. These compounds were further tested against the multi-drug resistant K1 strain of *P. falciparum*. Compound **12** showed about two-fold improvement in resistivity index ((RI = 5.36), *Pf*K1 IC_50_ = 0.59 μM) relative to CQ, making it the frontrunner compound in the present study. Meanwhile, compounds **8** and **11** were less active against the K1 strain with IC_50_ values of 2.97 μM and 6 μM, respectively.

In accordance with literature precedents [7], compounds with three methylene groups (n = 3) as linker showed greater activity against *P. falciparum* than those with two methylene groups (n = 2). Indeed, the introduction of an additional CH_2_ group (n = 3) in compound **12** enhances its potency approximately five-fold as compared to compound **17** (n = 2), while a two-fold increase is noticed going from compound **9** (n = 2, 0.28 μM) to compound **24** (n = 3, 0.57 μM). In addition, almost all molecular hybridscontaining three methylene groups as linker (**7–12**, **14–20**) were amongst the most active of the series, with IC_50_ values ranging from 0.10–0.86 μM.

The less polar methyl group has very little effect on the activity as is observed in compounds **7** and **22** which both have a methyl group in *para* position and exhibited the same activity (IC_50_ = 0.37 μM and 0.39 μM, respectively).

Compounds **21** and **23**, respectively, containing a 2-furanyl and ferrocenyl as the aromatic rings are among the less active, suggesting the importance of the chalcone unit in the overall activity of the molecule.

Except for compounds **7**, **17**, **18**, **22,** and **26**, most compounds were poorly soluble (<5 μM). As previously noted, a methyl group has no significant effect on the activity but its presence in compounds **7** and **22** significantly increases the solubility (130 and 190 μM, respectively) most likely by the disruption of the crystalline packing [35,36]. The high solubility of compounds **17** and **26** (185 and 200 μM) may be attributed to a combination of steric and polar effect of the electronegative oxygen atoms.

## 4. Materials and Methods

### 4.1. General

All commercially available chemicals were purchased from local chemical suppliers. Analytical thin-layer chromatography (TLC) was performed on aluminium-backed silica-gel 60 F254 (70–230 mesh) plates. Flash column chromatography was performed with Merck silica-gel 60 (70–230 mesh) on a Biotage Isolera ™ system (Biotage AB, Uppsala, Sweden). Products were characterized by ^1^H- and ^13^C-NMR spectra recorded on a 300 MHz, 400 MHz or 600 MHz Varian or Bruker NMR spectrometers. Chemical shifts (δ) are given in ppm downfield from trimethylsilane (TMS) as the internal standard. Coupling constants (*J*), are recorded in hertz (Hz). Purity was determined by HPLC. The NMR spectra and HPLC-MS spectra of compounds described in this study can be found in Supplementary Materials.

**Aqueous solubility.** Water solubility was analyzed using a miniaturized shake flask method. Ten millimolar stock solutions of each of the compounds were used to prepare calibration standards (10−220 μM) in DMSO. The same 10 mM stock solutions were accurately dispensed in duplicate into 96-well plates, and the DMSO, dried down (MiVac GeneVac,90 min, 37 °C). Thereafter, the samples were reconstituted (200 μM) in an aqueous solution and shaken (20 h, 25 °C). The solutions were analyzed by means of HPLC-DAD (Agilent1200 Rapid Resolution HPLC with a diode array detector). Best fit calibration curves were constructed using the calibration standards, which were used to determine the aqueous samples’ solubility [1,37].

**In-vitro anti-plasmodium assay.** Compounds were tested using parasite lactate dehydrogenase assay as a marker for parasite survival. The respective stock solutions of CQ diphosphate and test compounds were prepared by dissolving 2 mg/mL in distilled water (for CQ) and 100% DMSO for test compounds. The solutions were then stored at −20 °C, with further dilutions prepared on the day of the experiment. The cultures were synchronized in the ring stage as described previously using 15 mL of 5% (*w*/*v*) d-sorbitol in water. Synchronous cultures of *Pf*NF54 (CQS) in the late trophozoite stage were prepared to 2% parasitemia and 2% hematocrit. Compounds were tested at starting concentrations of 10 000 ng/mL (1000 ng/mL for CQ), which were then serially diluted two-fold in complete medium to give 10 concentrations with a final volume of 200 μL in each well. Parasites were incubated in the presence of the compounds at 37 °C in a specialized atmosphere of 4% CO_2_ and 3% O_2_ in nitrogen for 48 h. Following incubation, 100 μL of MalStat reagent and 15 μL of resuspended culture were combined, followed by addition of 25 μL of nitro blue tetrazolium chloride (NBT). The plates were kept in the dark for about 10 min to fully develop, and absorbance was measured at 620 nm on a microplate reader. Raw data were exported to Microsoft Excel for dose-response analysis [1,37].

**Cytotoxicity assay.** Compounds were screened for in vitro cytotoxicity against Chinese hamster ovarian (CHO) mammalian cell lines, using the 3-(4,5-dimethylthiazol-2-yl)-2,5-diphenyltetrazolium bromide (MTT) assay. The reference standard, emetine, was prepared to 2 mg/mL in distilled water while stock solutions of test compounds were prepared to 20 mg/mL in 100% DMSO with the highest concentration of solvent to which the cells were exposed having no measurable effect on the cell viability. The initial concentration of the compounds and control was 100 μg/mL, which was serially diluted in complete medium with 10-fold dilutions to give six concentrations, the lowest being 0.001 μg/mL. Plates were incubated for 48 h with 100 μL of drug and 100 μL of cell suspension in each well and developed afterward by adding 25 μL of sterile MTT (Thermo Fisher Scientific) to each well, followed by 4 h of incubation in the dark. The plates were then centrifuged; the medium was aspirated, and 100 μL of DMSO was added to dissolve crystals before reading the absorbance at 540 nm. Data were analyzed, and the sigmoidal dose-response was derived using GraphPad Prism v 4.0 software (La Jolla). All experiments were performed for at least three independent biological repeats, each with technical triplicates [1].

### 4.2. Experimental Section

General procedure for preparing 4-acetyl-N-(((7-chloroquinolin-4-yl)amino)alkyl) benzenesulfonamides **4** and **5**: 4-acetylbenzenesulfonyl chloride was treated with the respective N-(7-chloroquinolin-4-yl) alkyldiamine (**2** or **3**) in 7.5 mL dioxane as solvent and 2.5 mL 10 M NaOH. The reaction mixture was stirred at room temperature for 24 h and poured into 50 mL of ice-cold water. The precipitate was filtered, dried and the crude product purified using Biotage Isolera One^®^ column chromatography in a gradient mixture of CH_2_Cl_2_/MeOH. Fractions were monitored and collected at 254 and 280 nm. The solvent of the combined fractions was removed under reduced pressure to afford compounds **4** and **5** as white solids in 27 and 41% yield, respectively.

*4-acetyl-N-(2-((7-chloroquinolin-4-yl)amino)ethyl)benzenesulfonamide* (**4**); white solid (0.97 g, 27%); m.p. 200–204 °C; Rf = 0.61 (MeOH-CH_2_Cl_2_ 1:9); ^1^H NMR (600 MHz, DMSO-*d*_6_) δ 8.35 (d, *J* = 5.4 Hz, 1H), 8.09 (d, *J* = 9.1 Hz, 1H), 8.01 (t, *J* = 5.7 Hz, 1H), 7.99–7.94 (m, 2H), 7.90–7.84 (m, 2H), 7.75 (d, *J* = 2.2 Hz, 1H), 7.43 (dd, *J* = 9.0, 2.3 Hz, 1H), 7.23 (t, *J* = 5.8 Hz, 1H), 6.40 (d, *J* = 5.5 Hz, 1H), 3.37 (q, *J* = 6.3 Hz, 2H), 3.10 (t, *J* = 6.1 Hz, 2H), 2.56 (s, 3H). ^13^C NMR (151 MHz, DMSO-*d*_6_) δ 197.48, 152.10, 150.20, 149.24, 144.55, 139.67, 133.99, 129.31, 127.81, 127.15, 124.63, 124.38, 117.81, 99.02, 42.46, 41.26, 27.34; HPLC-MS (APCI/ESI): Purity = 80%. t_R_ = 0.898 min, calcd. *m*/*z* = 403.88, *m*/*z* [M]^+^ = 404.0.

*4-acetyl-N-(3-((7-chloroquinolin-4-yl)amino)propyl)benzenesulfonamide* (**5**); white solid (0.144 g, 41%); m.p. 181–183 °C; Rf = 0.46 (MeOH-CH_2_Cl_2_,1:9); ^1^H NMR (600 MHz, DMSO-*d*_6_) δ 8.36 (d, *J* = 5.4 Hz, 1H), 8.19 (d, *J* = 9.0 Hz, 1H), 8.08–8.03 (m, 2H), 7.92–7.84 (m, 3H), 7.77 (d, *J* = 2.2 Hz, 1H), 7.43 (dd, *J* = 9.0, 2.3 Hz, 1H), 7.21 (t, *J* = 5.5 Hz, 1H), 6.38 (d, *J* = 5.5 Hz, 1H), 3.24 (td, *J* = 6.9, 5.3 Hz, 2H), 2.97–2.90 (m, 2H), 2.59 (s, 3H), 1.77 (p, *J* = 7.0 Hz, 2H). ^13^C NMR (151 MHz, DMSO-*d*_6_) δ 197.64, 152.17, 150.45, 149.33, 144.60, 139.79, 133.90, 129.41, 127.83, 127.24, 124.55, 124.49, 117.85, 99.14, 40.57, 40.31, 28.22, 27.41; HPLC-MS (APCI/ESI): Purity = 100%. t_R_ = 2.186 min, calcd. *m*/*z* = 417.91, *m*/*z* [M]^+^ = 418.0.

General procedure for the synthesis of molecular hybrids **6**–**27**: To a solution of chloroquinolinesulfonamide (4 or 5) and aryl aldehyde derivatives in methanol (2 mL), NaOH (24 mg, 6 mmoles, 2.5 equiv.) was added. The reaction mixture was stirred under reflux overnight. The product was purified using Biotage Isolera One ^®^ column chromatography in a gradient mixture of CH_2_Cl_2_/MeOH. Fractions were monitored and collected at 254 and 280 nm. The solvent of the combined fractions was removed under reduced pressure. In some cases, the products were further purified by recrystallization in CH_2_Cl_2_ and MeOH to afford crystalline solids with 11–88% yield.

*N-(3-((7-chloroquinolin-4-yl)amino)propyl)-4-cinnamoylbenzenesulfonamide* (**6**); white solid (0.087 g, 72%); m.p. 210–212 °C; Rf = 0.39 (MeOH-CH_2_Cl_2_, 1:9); ^1^H NMR (600 MHz, DMSO-*d*_6_) δ 8.37 (d, *J* = 5.4 Hz, 1H), 8.28–8.23 (m, 2H), 8.21 (d, *J* = 9.1 Hz, 1H), 7.97–7.92 (m, 2H), 7.92–7.88 (m, 3H), 7.88 (d, *J* = 13.5 Hz, 1H), 7.78 (d, *J* = 15.7 Hz, 1H), 7.76 (d, *J* = 2.2 Hz, 1H), 7.64–7.58 (m, 1H), 7.53–7.46 (m, 2H), 7.46–7.36 (m, 1H), 7.30 (t, *J* = 5.4 Hz, 1H), 6.40 (d, *J* = 5.5 Hz, 1H), 3.25 (q, *J* = 6.6 Hz, 2H), 2.96 (q, *J* = 6.7 Hz, 2H), 1.79 (p, *J* = 7.0 Hz, 2H). ^13^C NMR (151 MHz, DMSO-*d*_6_) δ 188.99, 167.83, 151.87, 150.65, 145.48, 144.52, 140.76, 134.96, 134.06, 133.24, 131.39, 129.77, 129.71, 127.55, 127.31, 124.64, 124.54, 122.35, 117.79, 99.14, 41.01, 40.56, 28.25; HPLC-MS (APCI/ESI): Purity = 100%. tR = 2.470 min, calcd. *m*/*z* = 506.0, *m*/*z* [M]^+^ = 506.0.

*(E)-N-(3-((7-chloroquinolin-4-yl)amino)propyl)-4-(3-(p-tolyl)acryloyl)benzenesulfonamide* (**7**); white solid (0.0738 g, 59%); m.p. 209–213 °C; Rf = 0.48 (MeOH-CH_2_Cl_2_, 1:9); ^1^H NMR (600 MHz, DMSO-*d*_6_) δ 8.36 (d, *J* = 5.4 Hz, 1H), 8.27–8.22 (m, 2H), 8.20 (d, *J* = 9.0 Hz, 1H), 7.96–7.91 (m, 2H), 7.89 (t, *J* = 5.8 Hz, 1H), 7.83 (d, *J* = 15.6 Hz, 1H), 7.81–7.78 (m, 2H), 7.78–7.73 (m, 2H), 7.42 (dd, *J* = 9.0, 2.2 Hz, 1H), 7.30 (d, *J* = 7.9 Hz, 2H), 7.23 (t, *J* = 5.4 Hz, 1H), 6.39 (d, *J* = 5.5 Hz, 1H), 3.24 (t, *J* = 6.5 Hz, 2H), 2.96 (q, *J* = 6.7 Hz, 2H), 2.37 (s, 3H), 1.79 (q, *J* = 7.0 Hz, 2H). ^13^C NMR (151 MHz, DMSO-*d*_6_) δ 188.92, 152.13, 150.50, 149.28, 145.58, 144.43, 141.57, 140.90, 133.92, 132.26, 130.06, 129.71, 129.57, 127.79, 127.29, 124.56, 124.49, 121.28, 117.85, 99.15, 41.03, 40.17, 28.27, 21.60; HPLC-MS (APCI/ESI): Purity = 100%, t_R_ = 2.528 min, calcd. *m*/*z* = 520.0, *m*/*z* [M]^+^ = 520.1.

*(E)-4-(3-(2-bromophenyl)acryloyl)-N-(3-((7-chloroquinolin-4-yl)amino)propyl)benzenesulfonamide* (**8**); white solid (0.1089 g, 78%); m.p. 211–213 °C; Rf = 0.48 (MeOH-CH_2_Cl_2_, 1:9); ^1^H NMR (600 MHz, DMSO-*d*_6_) δ 8.36 (d, *J* = 5.4 Hz, 1H), 8.28–8.24 (m, 2H), 8.23–8.16 (m, 2H), 8.02 (d, *J* = 15.5 Hz, 1H), 7.95 (s, 1H), 7.94 (d, *J* = 2.0 Hz, 1H), 7.92–7.85 (m, 2H), 7.80–7.72 (m, 2H), 7.56–7.49 (m, 1H), 7.49–7.39 (m, 2H), 7.26–7.16 (m, 1H), 6.44–6.36 (m, 1H), 3.24 (q, *J* = 6.5 Hz, 2H), 2.96 (q, *J* = 6.6 Hz, 2H), 1.77 (h, *J* = 7.1 Hz, 2H). ^13^C NMR (151 MHz, DMSO-*d*_6_) δ 188.25, 151.78, 151.75, 149.91, 148.95, 144.22, 142.09, 139.90, 133.74, 133.33, 132.42, 129.39, 128.82, 128.24, 127.40, 126.82, 125.50, 124.62, 124.05, 123.95, 117.36, 98.64, 40.50, 40.46, 27.71; HPLC-MS (APCI/ESI): Purity = 100%. t_R_ = 2.530 min, calcd. *m*/*z* = 584.9, *m*/*z* [M + H]^+^ = 585.9.

*(E)-N-(3-((7-chloroquinolin-4-yl)amino)propyl)-4-(3-(4-fluorophenyl)acryloyl)benzenesulfonamide* (**9**); white solid (0.0763 g, 61%); m.p. 216–218 °C; Rf = 0.46 (MeOH-CH_2_Cl_2_, 1:9); ^1^H NMR (600 MHz, DMSO-*d*_6_) δ 8.37 (d, *J* = 5.5 Hz, 1H), 8.27–8.23 (m, 2H), 8.21 (d, *J* = 9.0 Hz, 1H), 8.02–7.73 (m, 8H), 7.44 (dd, *J* = 8.9, 2.2 Hz, 1H), 7.35–7.28 (m, 3H), 6.41 (d, *J* = 5.5 Hz, 1H), 3.26 (q, *J* = 6.5 Hz, 2H), 2.96 (q, *J* = 6.6 Hz, 2H), 1.78 (p, *J* = 7.0 Hz, 2H). ^13^C NMR (151 MHz, DMSO-*d*_6_) δ 188.86, 164.04 (d, *J*C-F = 249.6 Hz), 151.65, 150.78, 144.52, 144.23, 140.73, 134.17, 131.95, 131.93 (d, *J*C-F = 8.5 Hz), 131.68, 129.77, 127.34, 127.30, 124.71, 124.57, 122.23, 117.74, 116.47 (d, *J*C-F = 21.8 Hz), 99.14, 41.00, 40.23, 28.24. HPLC-MS (APCI/ESI); Purity = 100%, t_R_ = 2.476 min, calcd. *m*/*z* = 524.0, *m*/*z* [M]+ = 524.0.

*(E)-N-(3-((7-chloroquinolin-4-yl)amino)propyl)-4-(3-(2,6-difluorophenyl)acryloyl)benzenesulfonamide* (**10**); white solid (0.1216 g, 70%); m.p. 223–225 °C; Rf = 0.44 (MeOH-CH_2_Cl_2_, 1:9); ^1^H NMR (600 MHz, DMSO-*d*_6_) δ 8.34 (d, *J* = 5.3 Hz, 1H), 8.17 (d, *J* = 9.0 Hz, 1H), 8.15–8.11 (m, 2H), 7.97–7.92 (m, 2H), 7.90 (q, *J* = 5.5 Hz, 1H), 7.77 (s, 1H), 7.75–7.68 (m, 2H), 7.59 (tt, *J* = 8.2, 6.4 Hz, 1H), 7.40 (dd, *J* = 9.0, 2.3 Hz, 1H), 7.27 (t, *J* = 8.8 Hz, 2H), 7.16 (t, *J* = 5.5 Hz, 1H), 6.37 (d, *J* = 5.4 Hz, 1H), 3.23 (q, *J* = 6.5 Hz, 2H), 2.96 (q, *J* = 6.5 Hz, 2H), 1.78 (p, *J* = 6.9 Hz, 2H). ^13^C NMR (151 MHz, DMSO-*d*_6_) δ 188.48, 161.10 (d, *J*C-F = 253.9 Hz), 151.81, 149.92, 148.98, 144.27, 139.69, 133.32, 132.89 (t, *J*C-F = 11.2 Hz), 130.08, 129.20, 127.42, 127.17, 126.99, 124.02, 123.98, 117.39, 112.49, 112.32 (d, *J*C-F = 21.7 Hz), 98.63, 40.49, 40.04, 27.78. HPLC-MS (APCI/ESI); Purity = 100%, t_R_ = 2.477 min, calcd. *m*/*z* = 542.0, *m*/*z* [M]^+^ = 542.0.

*(E)-4-(3-(4-chlorophenyl)acryloyl)-N-(3-((7-chloroquinolin-4-yl)amino)propyl)benzenesulfonamide* (**11**); white solid (0.045 g, 35%); m.p. 222–225 °C; Rf = 0.47 (MeOH-CH_2_Cl_2_, 1:9); ^1^H NMR (600 MHz, DMSO-*d*_6_) δ 8.35 (d, *J* = 5.3 Hz, 1H), 8.27–8.23 (m, 2H), 8.18 (d, *J* = 9.0 Hz, 1H), 7.95 (s, 1H), 7.96–7.88 (m, 5H), 7.77 (d, *J* = 15.7 Hz, 1H), 7.74 (d, *J* = 2.2 Hz, 1H), 7.57–7.52 (m, 2H), 7.41 (dd, *J* = 9.0, 2.3 Hz, 1H), 7.18 (t, *J* = 5.4 Hz, 1H), 6.37 (d, *J* = 5.4 Hz, 1H), 3.23 (q, *J* = 6.5 Hz, 2H), 2.96 (t, *J* = 6.9 Hz, 2H), 1.78 (p, *J* = 7.0 Hz, 2H). ^13^C NMR (151 MHz, DMSO-*d*_6_) δ 188.36, 151.80, 149.87, 148.98, 144.08, 143.44, 140.11, 135.37, 133.44, 133.32, 130.69, 129.30, 128.96, 127.45, 126.79, 123.99, 123.93, 122.54, 117.37, 98.63, 48.55, 40.51, 27.73; HPLC-MS (APCI/ESI): Purity = 100%. t_R_ = 2.527 min, calcd. *m*/*z* = 540.4, *m*/*z* [M]^+^ = 540.0.

*(E)-N-(3-((7-chloroquinolin-4-yl)amino)propyl)-4-(3-(4-methoxyphenyl)acryloyl)benzenesulfonamide* (**12**); white solid (0.0261 g, 20%); m.p. 221–223 °C; Rf = 0.45 (MeOH-CH_2_Cl_2_, 1:9); ^1^H NMR (600 MHz, DMSO-*d*_6_) δ 8.36 (d, *J* = 5.4 Hz, 1H), 8.25–8.21 (m, 2H), 8.19 (d, *J* = 9.0 Hz, 1H), 7.98–7.91 (m, 2H), 7.91–7.84 (m, 3H), 7.75 (d, *J* = 3.0 Hz, 3H), 7.42 (dd, *J* = 9.0, 2.3 Hz, 1H), 7.20 (t, *J* = 5.5 Hz, 1H), 7.06–7.01 (m, 2H), 6.38 (d, *J* = 5.4 Hz, 1H), 3.83 (s, 3H), 3.26–3.21 (m, 2H), 2.96 (q, *J* = 6.6 Hz, 2H), 1.78 (p, *J* = 7.0 Hz, 2H). ^13^C NMR (151 MHz, DMSO-*d*_6_) δ 188.29, 161.63, 151.76, 149.93, 148.92, 145.05, 140.62, 133.38, 131.88, 131.00, 129.14, 127.40, 127.11, 126.77, 124.04, 123.96, 119.30, 117.37, 114.46, 98.65, 55.40, 54.85, 40.53, 27.76; HPLC-MS (APCI/ESI): Purity = 100%. t_R_ = 2.471 min, calcd. *m*/*z* = 536.04, *m*/*z* [M]^+^ = 536.1.

*(E)-N-(3-((7-chloroquinolin-4-yl)amino)propyl)-4-(3-(3,4-difluorophenyl)acryloyl)benzenesulfonamide* (**13**); light yellow solid (0.0651 g, 38%); m.p. 212–214 °C; Rf = 0.43 (MeOH-CH_2_Cl_2_, 1:9); ^1^H NMR (600 MHz, DMSO-*d*_6_) δ 8.35 (d, *J* = 5.4 Hz, 1H), 8.28–8.23 (m, 2H), 8.18 (d, *J* = 9.1 Hz, 1H), 8.13 (ddd, *J* = 12.1, 7.9, 2.1 Hz, 1H), 7.96–7.88 (m, 4H), 7.78–7.72 (m, 3H), 7.54 (dt, *J* = 10.6, 8.5 Hz, 1H), 7.41 (dd, *J* = 9.0, 2.3 Hz, 1H), 7.20 (t, *J* = 5.4 Hz, 1H), 6.37 (d, *J* = 5.4 Hz, 1H), 3.24 (q, *J* = 6.5 Hz, 2H), 2.97 (q, *J* = 6.5 Hz, 2H), 1.78 (p, *J* = 7.0 Hz, 2H). ^13^C NMR (151 MHz, DMSO-*d*_6_) δ 188.22, 151.71, 151.67, 149.9 (d, *J*C-F =10.9 Hz), 148.92 (d, *J*C-F = 13.3 Hz), 142.59, 140.01, 132.44 (d, *J*C-F = 2.8 Hz), 129.51, 129.34, 127.36, 127.0 (d, *J*C-F = 3.4 Hz), 126.98 (d, *J*C-F =6.9 Hz), 123.95, 123.01, 118.06,118.0 (d, *J*C-F = 17.4 Hz), 117.98, 117.35, 117.26, 117.08, 98.63, 40.51, 40.04, 27.73; HPLC-MS (APCI/ESI): Purity = 100%. t_R_ = 2.475 min, calcd. *m*/*z* = 542.11, *m*/*z* [M]^+^ = 542.0.

*(E)-N-(3-((7-chloroquinolin-4-yl)amino)propyl)-4-(3-(3,4-dimethoxyphenyl)acryloyl)benzenesulfonamide* (**14**); white solid (0.0146 g, 11%); m.p. 222–224 °C; Rf = 0.37 (MeOH-CH_2_Cl_2_, 1:9); ^1^H NMR (600 MHz, DMSO-*d*_6_) δ 8.36 (d, *J* = 5.4 Hz, 1H), 8.26–8.22 (m, 2H), 8.19 (d, *J* = 9.0 Hz, 1H), 7.96–7.91 (m, 2H), 7.89 (t, *J* = 5.7 Hz, 1H), 7.80–7.71 (m, 3H), 7.55 (d, *J* = 2.0 Hz, 1H), 7.45–7.39 (m, 2H), 7.20 (t, *J* = 5.5 Hz, 1H), 7.04 (d, *J* = 8.4 Hz, 1H), 6.38 (d, *J* = 5.5 Hz, 1H), 3.87 (s, 3H), 3.83 (s, 3H), 3.24 (q, *J* = 6.5 Hz, 2H), 2.96 (q, *J* = 6.6 Hz, 2H), 1.78 (p, *J* = 7.0 Hz, 2H). ^13^C NMR (151 MHz, DMSO-*d*_6_) δ 188.33, 151.76, 151.58, 149.92, 149.03, 148.92, 145.57, 143.79, 140.67, 133.36, 129.14, 127.39, 127.29, 126.74, 124.25, 124.03, 123.95, 119.38, 117.37, 111.59, 110.96, 98.65, 55.75, 55.62, 40.52, 40.04, 27.76; HPLC-MS (APCI/ESI): Purity = 100%. t_R_ = 2.417 min, calcd. *m*/*z* = 566.07, *m*/*z* [M]^+^ = 566.1.

*(E)-N-(3-((7-chloroquinolin-4-yl)amino)propyl)-4-(3-(2-fluorophenyl)acryloyl)benzenesulfonamide* (**15**); white solid (0.1093 g, 65%); m.p. 223–225 °C; Rf = 0.40 (MeOH-CH_2_Cl_2_, 1:9); ^1^H NMR (600 MHz, DMSO-*d*_6_) δ 8.35 (d, *J* = 5.5 Hz, 1H), 8.25–8.22 (m, 2H), 8.22–8.16 (m, 1H), 8.14–8.06 (m, 1H), 7.94 (dd, *J* = 7.6, 5.9 Hz, 2H), 7.92–7.81 (m, 3H), 7.78–7.70 (m, 1H), 7.58–7.49 (m, 1H), 7.41 (dd, *J* = 9.0, 2.3 Hz, 1H), 7.37–7.31 (m, 2H), 7.24–7.16 (m, 1H), 6.37 (d, *J* = 5.4 Hz, 1H), 3.24 (p, *J* = 6.5, 6.1 Hz, 2H), 2.96 (q, *J* = 6.5 Hz, 2H), 1.78 (p, *J* = 7.1 Hz, 2H). ^13^C NMR (151 MHz, DMSO-*d*_6_) δ 188.31, 160.98 (d, *J*C-F = 252.0 Hz), 151.81, 149.93, 148.98, 144.20, 139.95, 136.08, 133.36, 132.99,132.96 (d, *J*C-F = 8.8 Hz), 129.29 (d, *J*C-F = 3.0 Hz), 127.42, 127.38 (d, *J*C-F = 9.4 Hz), 127.01, 126.75, 124.98 (d, *J*C-F = 3.3 Hz), 124.03, 122.07, 117.40, 116.11 (d, *J*C-F = 21.5 Hz), 98.67, 40.51, 40.04, 27.72. HPLC-MS (APCI/ESI); Purity = 100%, t_R_ = 2.444 min, calcd. *m*/*z* = 524.0, *m*/*z* [M]^+^ = 524.1.

*(E)-N-(3-((7-chloroquinolin-4-yl)amino)propyl)-4-(3-(3,4,5-trimethoxyphenyl)acryloyl)benzenesulfonamide* (**16**); white solid (0.1088 g, 76%); m.p. 197–200 °C; Rf = 0.48 (MeOH-CH_2_Cl_2_, 1:9); ^1^H NMR (600 MHz, DMSO-*d*_6_) δ 8.37 (d, *J* = 5.5 Hz, 1H), 8.24–8.18 (m, 3H), 7.96–7.91 (m, 3H), 7.89 (t, *J* = 5.8 Hz, 1H), 7.82–7.73 (m, 3H), 7.43 (dd, *J* = 9.0, 2.2 Hz, 1H), 7.30 (t, *J* = 5.5 Hz, 1H), 6.95 (d, *J* = 8.9 Hz, 1H), 6.40 (d, *J* = 5.5 Hz, 1H), 3.89 (s, 3H), 3.88 (s, 3H), 3.78 (s, 3H), 3.25 (t, *J* = 6.5 Hz, 2H), 2.96 (q, *J* = 6.6 Hz, 2H), 1.78 (p, *J* = 7.0 Hz, 2H). ^13^C NMR (151 MHz, DMSO-*d*_6_) δ 188.88, 156.58, 153.74, 151.86, 150.66, 148.96, 144.32, 142.24, 141.13, 139.99, 134.06, 129.60, 127.54, 127.29, 124.65, 124.53, 124.19, 121.27, 120.66, 117.79, 109.00, 99.14, 62.03, 60.96, 56.62, 41.02, 40.57, 28.26; HPLC-MS (APCI/ESI): Purity = 100%, t_R_ = 2.459 min, calcd *m*/*z* = 596.1, *m*/*z* [M]^+^ = 596.0.

*(E)-N-(2-((7-chloroquinolin-4-yl)amino)ethyl)-4-(3-(4-methoxyphenyl)acryloyl)benzenesulfonamide* (**17**); yellow solid (0.1144 g, 89%); m.p. 222–225 °C; Rf = 0.38 (MeOH-CH_2_Cl_2_, 1:9); ^1^H NMR (600 MHz, DMSO-*d*_6_) δ 8.36 (d, *J* = 5.3 Hz, 1H), 8.19–8.14 (m, 2H), 8.10 (d, *J* = 9.0 Hz, 1H), 8.04 (t, *J* = 5.9 Hz, 1H), 7.94–7.89 (m, 2H), 7.89–7.83 (m, 2H), 7.78–7.72 (m, 2H), 7.70 (d, *J* = 15.6 Hz, 1H), 7.43 (dd, *J* = 9.0, 2.2 Hz, 1H), 7.23 (t, *J* = 5.7 Hz, 1H), 7.06–7.01 (m, 2H), 6.41 (d, *J* = 5.5 Hz, 1H), 3.83 (s, 3H), 3.38 (q, *J* = 6.3 Hz, 2H), 3.11 (q, *J* = 6.3 Hz, 2H). ^13^C NMR (151 MHz, DMSO-*d*_6_) δ 188.64, 162.11, 152.19, 150.19, 149.36, 145.45, 144.25, 141.05, 133.96, 131.48, 129.56, 127.90, 127.63, 127.20, 124.62, 124.35, 119.78, 117.84, 114.95, 99.02, 55.91, 42.55, 41.29; HPLC-MS (APCI/ESI): Purity = 100%, t_R_ = 2.440 min, calcd. *m*/*z* = 522.12, *m*/*z* [M]^+^ = 522.0.

*(E)-N-(3-((7-chloroquinolin-4-yl)amino)propyl)-4-(3-(2,4-difluorophenyl)acryloyl)benzenesulfonamide* (**18**); light yellow solid (0.01118 g, 65%); m.p. 216–218 °C; Rf = 0.39 MeOH-CH_2_Cl_2_, 1:9); ^1^H NMR (600 MHz, DMSO-*d*_6_) δ 8.35 (dd, *J* = 5.4, 1.9 Hz, 1H), 8.25–8.16 (m, 3H), 8.07–8.03 (m, 1H), 7.94 (d, *J* = 8.3 Hz, 1H), 7.94–7.86 (m, 2H), 7.82–7.72 (m, 2H), 7.46–7.37 (m, 2H), 7.29–7.19 (m, 2H), 6.38 (dd, *J* = 5.5, 2.2 Hz, 1H), 3.24 (t, *J* = 6.4 Hz, 2H), 2.98–2.90 (m, 2H), 1.77 (h, *J* = 6.8 Hz, 2H). ^13^C NMR (151 MHz, DMSO-*d*_6_) δ 188.19, 162.54 (d, *J*C-F = 100.6 Hz), 161.32 (d, *J*C-F = 242.3 Hz), 151.64, 151.62, 149.97, 148.80, 144.18, 143.95, 133.42, 130.31, 129.10 (d, *J*C-F = 58.9 Hz), 128.91, 126.73, 124.06, 123.98, 123.60, 118.97 (d, *J*C-F = 3.7 Hz), 117.34, 112.65 (d, *J*C-F = 3.4 Hz), 112.50 (d, *J*C-F = 3.7 Hz), 98.64, 40.49, 39.90, 27.70; HPLC-MS (APCI/ESI): Purity = 96%, t_R_ = 2.464 min, calcd. *m*/*z* = 541.99, *m*/*z* [M]^+^ = 542.0.

*(E)-N-(2-((7-chloroquinolin-4-yl)amino)ethyl)-4-(3-(3,4-dimethoxyphenyl)acryloyl)benzenesulfonamide* (**19**); yellow solid (0.0609 g, 45%); m.p. 223–225 °C; Rf = 0.44 (MeOH-CH_2_Cl_2_, 1:9); ^1^H NMR (600 MHz, DMSO-*d*_6_) δ 8.36 (d, *J* = 5.4 Hz, 1H), 8.18 (d, *J* = 8.2 Hz, 2H), 8.09 (d, *J* = 9.0 Hz, 1H), 7.92 (d, *J* = 8.4 Hz, 2H), 7.77–7.71 (m, 3H), 7.54 (d, *J* = 2.0 Hz, 1H), 7.42 (ddd, *J* = 7.9, 5.9, 2.1 Hz, 2H), 7.22 (t, *J* = 5.8 Hz, 1H), 7.04 (d, *J* = 8.4 Hz, 1H), 6.41 (d, *J* = 5.4 Hz, 1H), 3.86 (s, 3H), 3.83 (s, 3H), 3.38 (q, *J* = 6.4 Hz, 2H), 3.17 (d, *J* = 4.7 Hz, 2H). ^13^C NMR (151 MHz, DMSO-*d*_6_) δ 188.24, 151.83, 151.79, 151.57, 149.65, 149.02, 145.49, 143.73, 140.64, 133.42, 129.08, 127.47, 127.31, 126.68, 124.33, 124.19, 123.83, 119.40, 117.35, 111.60, 110.98, 98.52, 55.75, 48.56, 42.04; HPLC-MS (APCI/ESI): Purity = 100%. t_R_ = 2.387 min, calcd. *m*/*z* = 552.04, *m*/*z* [M]^+^ = 552.1.

*(E)-N-(3-((7-chloroquinolin-4-yl)amino)propyl)-4-(3-(2,5-difluorophenyl)acryloyl)benzenesulfonamide* (**20**); light yellow solid (0.1134 g, 66%); m.p. 219–221 °C; Rf = 0.39 (MeOH-CH_2_Cl_2_, 1:9); ^1^H NMR (600 MHz, DMSO-*d*_6_) δ 8.35 (d, *J* = 5.5 Hz, 1H), 8.27–8.23 (m, 2H), 8.19 (d, *J* = 9.0 Hz, 1H), 8.11–8.02 (m, 1H), 7.99 (d, *J* = 15.7 Hz, 1H), 7.95–7.87 (m, 2H), 7.79 (dd, *J* = 15.8, 1.4 Hz, 1H), 7.77–7.71 (m, 1H), 7.46–7.37 (m, 3H), 7.24 (t, *J* = 5.5 Hz, 1H), 6.38 (d, *J* = 5.5 Hz, 1H), 3.24 (q, *J* = 6.6 Hz, 2H), 2.97 (q, *J* = 6.6 Hz, 2H), 1.78 (p, *J* = 7.0 Hz, 2H). ^13^C NMR (151 MHz, DMSO-*d*_6_) δ 188.07, 158.59 (d, *J*C-F = 180.3 Hz), 156.97 (d, *J*C-F = 188.9 Hz), 155.42, 151.50, 150.04, 148.63, 144.30, 139.72, 134.64, 133.47, 129.39, 127.22, 126.84, 125.00, 124.99 (d, *J*C-F = 3.5 Hz), 123.99, 119.44 (d, *J*C-F = 9.0 Hz), 117.30, 117.07 (d, *J*C-F = 8.9 Hz), 114.87 (d, *J*C-F = 2.6 Hz), 98.63, 40.46, 40.04, 27.73; HPLC-MS (APCI/ESI): Purity = 100%. t_R_ = 2.490 min, calcd. *m*/*z* = 542.11, *m*/*z* [M]^+^ = 542.0.

*(E)-N-(3-((7-chloroquinolin-4-yl)amino)propyl)-4-(3-(furan-2-yl)acryloyl)benzenesulfonamide* (**21**); light yellow solid (0.0798 g, 50%); m.p. 222–224 °C; Rf = 0.41 (MeOH-CH_2_Cl_2_, 1:9); ^1^H NMR (600 MHz, DMSO-*d*_6_) δ 8.36 (d, *J* = 5.5 Hz, 1H), 8.21 (d, *J* = 9.0 Hz, 1H), 8.18–8.14 (m, 2H), 7.97–7.91 (m, 2H), 7.93–7.85 (m, 2H), 7.76 (d, *J* = 2.2 Hz, 1H), 7.60 (d, *J* = 15.4 Hz, 1H), 7.44 (dd, *J* = 9.4, 7.1 Hz, 1H), 7.15 (d, *J* = 3.4 Hz, 1H), 6.72 (dd, *J* = 3.5, 1.8 Hz, 1H), 6.40 (d, *J* = 5.6 Hz, 1H), 3.27–3.21 (m, 2H), 2.95 (q, *J* = 6.6 Hz, 2H), 1.78 (p, *J* = 6.9 Hz, 2H). ^13^C NMR (151 MHz, DMSO-*d*_6_) δ 187.89, 151.21, 150.98, 150.24, 148.29, 146.57, 143.89, 140.21, 133.63, 131.27, 129.05, 126.91, 126.88, 124.19, 124.05, 118.39, 117.83, 117.25, 113.27, 98.63, 48.56, 40.48, 27.72. HPLC-MS (APCI/ESI); Purity = 100%, t_R_ = 2.368 min, calcd. *m*/*z* = 495.97, *m*/*z* [M]^+^ = 496.0.

*(E)-N-(2-((7-chloroquinolin-4-yl)amino)ethyl)-4-(3-(p-tolyl)acryloyl)benzenesulfonamide* (**22**); yellow solid (0.0704 g, 56%); m.p. 184–187 °C; Rf = 0.39 (MeOH-CH_2_Cl_2_, 1:9); ^1^H NMR (600 MHz, DMSO-*d*_6_) δ 8.36 (d, *J* = 5.4 Hz, 1H), 8.20–8.14 (m, 2H), 8.10 (d, *J* = 9.1 Hz, 1H), 8.05 (t, *J* = 5.9 Hz, 1H), 7.94–7.89 (m, 2H), 7.82–7.71 (m, 5H), 7.43 (dd, *J* = 9.0, 2.2 Hz, 1H), 7.33–7.23 (m, 3H), 6.41 (d, *J* = 5.5 Hz, 1H), 3.38 (q, *J* = 6.3 Hz, 2H), 3.11 (q, *J* = 6.3 Hz, 2H), 2.36 (s, 3H). ^13^C NMR (151 MHz, DMSO-*d*_6_) δ 188.77, 152.10, 150.25, 149.25, 145.49, 144.38, 141.55, 140.81, 134.01, 132.27, 130.06, 129.63, 129.55, 127.81, 127.21, 124.64, 124.37, 121.24, 117.82, 99.02, 42.54, 41.29, 21.60; HPLC-MS (APCI/ESI): Purity = 100%, t_R_ = 2.481 min, calcd. *m*/*z* = 506.02, *m*/*z* [M]^+^ = 506.0.

*(E)-N-(2-((7-chloroquinolin-4-yl)amino)ethyl)-4-(3-ferrocenyl)acryloyl)benzenesulfonamide* **23**; purple solid (0.0601 g, 41%); m.p. 222–224 °C; Rf = 0.39 (MeOH-CH_2_Cl_2_, 1:9); ^1^H NMR (600 MHz, DMSO-*d*_6_) δ 8.41 (d, *J* = 7.5 Hz, 1H), 8.17–7.19 (m, 14H), 6.56–6.47 (m, 6H), 3.15 (q, *J* = 6.6 Hz, 2H), 2.95 (q, *J* = 6.6 Hz, 2H), 1.76 (p, *J* = 7.1 Hz, 2H). ^13^C NMR (151 MHz, DMSO-*d*_6_) δ 187.29, 151.60, 149.75, 147.70, 143.47, 140.72, 133.16, 132.01, 128.84, 127.47, 126.63, 123.90, 118.48, 78.66, 71.51, 69.54, 69.35, 54.73, 48.44, 27.65; HPLC-MS (APCI/ESI): Purity = 100%, t_R_ = 2.498 min, calcd. *m*/*z* = 613.9, *m*/*z* [M]^+^ = 614.0.

*(E)-N-(2-((7-chloroquinolin-4-yl)amino)ethyl)-4-(3-(4-fluorophenyl)acryloyl)benzenesulfonamide* (**24**); light yellow solid (0.0893 g, 71%); m.p. 222–224 °C Rf = 0.45 (MeOH-CH_2_Cl_2_, 1:9); ^1^H NMR (600 MHz, DMSO-*d*_6_) δ 8.37 (d, *J* = 5.5 Hz, 1H), 8.20–8.14 (m, 2H), 8.10 (d, *J* = 9.1 Hz, 1H), 8.05 (t, *J* = 5.9 Hz, 1H), 8.03–7.95 (m, 2H), 7.94–7.89 (m, 2H), 7.84–7.71 (m, 3H), 7.43 (dd, *J* = 9.0, 2.3 Hz, 1H), 7.36–7.28 (m, 3H), 6.42 (d, *J* = 5.5 Hz, 1H), 3.39 (q, *J* = 6.3 Hz, 2H), 3.12 (q, *J* = 6.3 Hz, 2H). ^13^C NMR (151 MHz, DMSO-*d*_6_) δ 188.71, 163.22 (d, *J*C-F = 249.6 Hz), 151.83, 150.41, 148.92, 144.48, 144.14, 134.14, 131.93 (d, *J*C-F = 8.5 Hz), 131.88, 131.68, 129.68, 127.21, 124.72, 124.42, 122.17, 117.75, 116.54 (d, *J*C-F = 21.8 Hz), 116.40, 99.02, 42.55, 41.30; HPLC-MS (APCI/ESI): Purity = 100%, t_R_ = 2.427 min, calcd. *m*/*z* = 509.98, *m*/*z* [M]^+^ = 510.0.

*(E)-4-(3-(2-chlorophenyl)acryloyl)-N-(2-((7-chloroquinolin-4-yl)amino)ethyl)benzenesulfonamide* (**25**); light yellow solid (0.0508 g, 39%); m.p. 209–211 °C Rf = 0.42 (MeOH-CH_2_Cl_2_, 1:9); ^1^H NMR (600 MHz, DMSO-*d*_6_) δ 8.43 (td, *J* = 8.7, 5.9 Hz, 1H), 8.30–8.25 (m, 2H), 8.25–6.90 (m, 12H), 6.75 (d, *J* = 9.7 Hz, 1H), 6.55 (d, *J* = 9.1 Hz, 1H), 3.45 (dd, *J* = 12.1, 5.8 Hz, 2H), 3.07 (q, *J* = 6.3 Hz, 2H). ^13^C NMR (151 MHz, DMSO-*d*_6_) δ 192.18, 144.60, 139.91, 138.38, 134.46, 132.86, 132.51, 131.08, 130.64, 130.00, 129.74, 129.60, 128.22, 127.39, 127.19, 126.90, 125.90, 125.55, 124.93, 124.13, 121.62, 99.04, 42.75, 41.23; HPLC-MS (APCI/ESI): Purity = 82%, t_R_ = 2.451 min, calcd. *m*/*z* = 526.4, *m*/*z* [M]^+^ = 526.0.

*(E)-4-(3-(benzo[d]*[1,3]*dioxol-5-yl)acryloyl)-N-(3-((7-chloroquinolin-4-yl)amino)propyl)benzenesulfonamide* (**26**); yellow solid (0.058 g, 66%); m.p. 222–224 °C; Rf = 0.46 (MeOH-CH_2_Cl_2_, 1:9); ^1^H NMR (600 MHz, DMSO-*d*_6_) δ 8.35 (d, *J* = 5.4 Hz, 1H), 8.25 (d, *J* = 8.2 Hz, 2H), 8.19 (d, *J* = 9.0 Hz, 1H), 7.94–7.64 (m, 7H), 7.42 (dd, *J* = 9.0, 2.3 Hz, 1H), 7.36 (dd, *J* = 8.1, 1.7 Hz, 1H), 7.19 (t, *J* = 5.6 Hz, 1H), 7.01 (d, *J* = 8.0 Hz, 1H), 6.38 (d, *J* = 5.5 Hz, 1H), 6.12 (s, 2H), 3.24 (q, *J* = 6.5 Hz, 2H), 2.96 (q, *J* = 6.6 Hz, 2H), 1.78 (p, *J* = 7.1 Hz, 2H). ^13^C NMR (151 MHz, DMSO-*d*_6_) δ 188.16, 151.79, 149.89, 149.85, 148.97, 148.12, 145.05, 143.86, 140.50, 133.34, 129.17, 129.00, 127.43, 126.75, 126.30, 124.01, 123.94, 119.71, 117.38, 108.55, 107.02, 101.72, 98.64, 40.53, 40.48, 27.76. HPLC-MS (APCI/ESI); Purity = 100%, t_R_ = 2.423 min, calcd. *m*/*z* = 550.0, *m*/*z* [M]^+^ = 550.0.

*(E)-N-(3-((7-chloroquinolin-4-yl)amino)propyl)-4-(3-(4-(trifluoromethoxy)phenyl)acryloyl)benzenesulfonamide* (**27**); light yellow solid (0.0901 g, 96% yield); m.p. 223–225 °C; Rf = 0.43 (10% MeOH-CH_2_Cl_2_); ^1^H NMR (600 MHz, DMSO-*d*_6_) δ 8.36 (d, *J* = 5.5 Hz, 1H), 8.27–8.23 (m, 2H), 8.20 (d, *J* = 8.9 Hz, 1H), 8.10–8.02 (m, 2H), 7.96–7.86 (m, 4H), 7.79 (d, *J* = 15.7 Hz, 1H), 7.74 (d, *J* = 2.3 Hz, 1H), 7.49–7.41 (m, 2H), 7.30 (t, J = 5.3 Hz, 1H), 6.40 (d, *J* = 5.5 Hz, 1H), 3.25 (q, J = 6.6 Hz, 2H), 2.96 (q, *J* = 6.5 Hz, 2H), 1.78 (p, *J* = 7.1 Hz, 2H). ^13^C NMR (151 MHz, DMSO-*d*_6_) δ 188.41, 151.30, 150.20, 149.74, 148.38, 144.12, 143.13, 140.08, 134.20, 133.61, 130.99, 129.33(d, *J*C-F = 25.3 Hz), 126.97, 126.74, 124.17, 124.04, 123.00, 121.26, 117.42, 117.27, 98.64, 40.49, 40.04, 27.72; HPLC-MS (APCI/ESI): Purity = 100%. t_R_ = 2.559 min, MW = 590.1, *m*/*z* [M]^+^ = 590.0.

## 5. Conclusions

In conclusion, 22 molecular hybrids were successfully synthesized in good to excellent yields. They were all fully characterized using spectroscopic techniques such as ^1^H- and ^13^C-NMR spectroscopy and HPLC-MS. These compounds were evaluated for in vitro antiplasmodial activity against the chloroquine sensitive (NF54) strain of *P. falciparum* and displayed promising activity with IC_50_ values ranging at 0.10–4.45 μM. The frontrunner compound **12** showed improved resistivity index compared to CQ, as well as good safety profile (SI = 393.6).

## Figures and Tables

**Table 1:** Hybrid compounds with isolated yields.

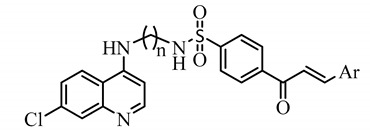			
Compound	n	Ar	Yield (%)
4	2	-	29
5	3	-	41
6	3	Ph	72
7	3	4-Me-Ph	59
8	3	2-Br-Ph	78
9	3	4-F-Ph	61
10	3	2,6-F_2_-Ph	70
11	3	4-Cl-Ph	35
12	3	4-OMe-Ph	20
13	3	3,4-F_2_-Ph	65
14	3	3,4-(OMe)_2_-Ph	11
15	3	2-F-Ph	66
16	3	3,4,5-(OMe)_3_-Ph	76
17	2	4-OMe-Ph	89
18	3	2,4-F_2_-Ph	38
19	2	3,4-(OMe)_2_-Ph	45
20	3	2,5-F_2_-Ph	66
21	3	2-furanyl	50
22	2	4-Me-Ph	56
23	3	ferrocenyl	31
24	2	4-F-Ph	71
25	2	2-Cl-Ph	39
26	3	3,4-OCH_2_O-Ph	66
27	3	4-OCF_3_-Ph	96

**Table 2:** In vitro antiplasmodial activity, solubility, and cytotoxicity of the hybrid compounds.

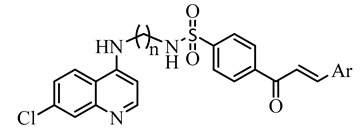						
Compound	n	Ar	*Pf*NF54 IC_50_ (μM) ^a^	Solubility ^b^ (μM) pH 6.5	CHO IC_50_ (μM) ^c^	SI ^d^
Chloroquine	-	-	0.011		-	-
Emetine					0.0034	
4	2	-	0.45	200	26.32	58.4
5	3	-	0.48	<5	>50	>104
6	3	Ph	1.67	ND	>50	>29
7	3	4-Me-Ph	0.37	130	25.08	67.8
8	3	2-Br-Ph	0.10	ND	43.25	435
9	3	4-F-Ph	0.28	<5	14.65	52.5
10	3	2,6-F_2_-Ph	0.54	<5	27.62	51.1
11	3	4-Cl-Ph	0.10	ND	41.2	416.2
12	3	4-OMe-Ph	0.11	ND	43.3	393.6
13	3	3,4-F_2_-Ph	2.58	<5	>300	>116
14	3	3,4-(OMe)_2_-Ph	0.32	ND	43.4	135.6
15	3	2-F-Ph	0.86	5	45.53	52.9
16	3	2,3,4-(OMe)_3_-Ph	0.58	15	22.62	39.0
17	2	4-OMe-Ph	0.49	185	17.53	35.7
18	3	2,4-F_2_-Ph	0.50	130	22.44	45.2
19	2	3,4-(OMe)_2_-Ph	0.33	ND	45.1	136.7
20	3	2,5-F_2_-Ph	0.79		43.7	55.3
21	3	2-furanyl	4.45	200	>50	>11
22	2	4-Me-Ph	0.39	190	24.89	63.8
23	3	ferrocenyl	1.53	ND		ND
24	2	4-F-Ph	0.57	<5	14.54	25.5
25	2	2-Cl-Ph	0.37	19	35.41	95.7
26	3	3,4-OCH_2_O-Ph	0.69	200	34.13	49.5
27	3	4-OCF_3_-Ph	0.51	ND	44.04	86.3

^a^ Mean from n values of ≥2 independent experiments with sensitive (NF54) strains of *P. falciparum*; ^b^ Aqueous solubility using high-performance liquid chromatography (HPLC)-diode array detector (DAD)-mass spectrometry (MS); ^c^ CHO = Chinese hamster ovarian cell line; ^d^ selectivity index (SI) = (IC50 (CHO)/IC50 (*Pf*NF54)). ND = not determined.

## Data Availability

Not applicable.

## Supplementary Materials

The following are available online. The NMR spectra and HPLC-MS spectra of compounds described in this study are available online.

## Sample Availability

Samples of the compounds are available from the authors.
