# Supplementary material for: Synthesis and Evaluation of Chalcone-Quinoline Based Molecular Hybrids as Potential Anti-Malarial Agents
Source: Molecules. 2021 Jul 5;26(13):4093. doi: 10.3390/molecules26134093 (PMC8272121; doi:10.3390/molecules26134093)
Supplement: Supplementary file 1 [file molecules-26-04093-s001.zip › molecules-1260629-supplementary.pdf]

SUPPLEMENTARY INFORMATION

Synthesis and Evaluation of Chalcone-Quinoline based Molecular Hybrids as potential Anti-Malarial Agents

Bonani Vinindwa[1], Godwin Akpeko Dziwornu[2], Wayiza Masamba[1*],.

[1]Department of Chemical and Physical Sciences, Faculty of Natural Sciences, Walter Sisulu University, Nelson Mandela Drive, Mthatha 5117, South Africa

[2]Department of Chemistry, University of Cape Town, Rondebosch 7700, South Africa.

Correspondence: wmasamba@wsu.ac.za

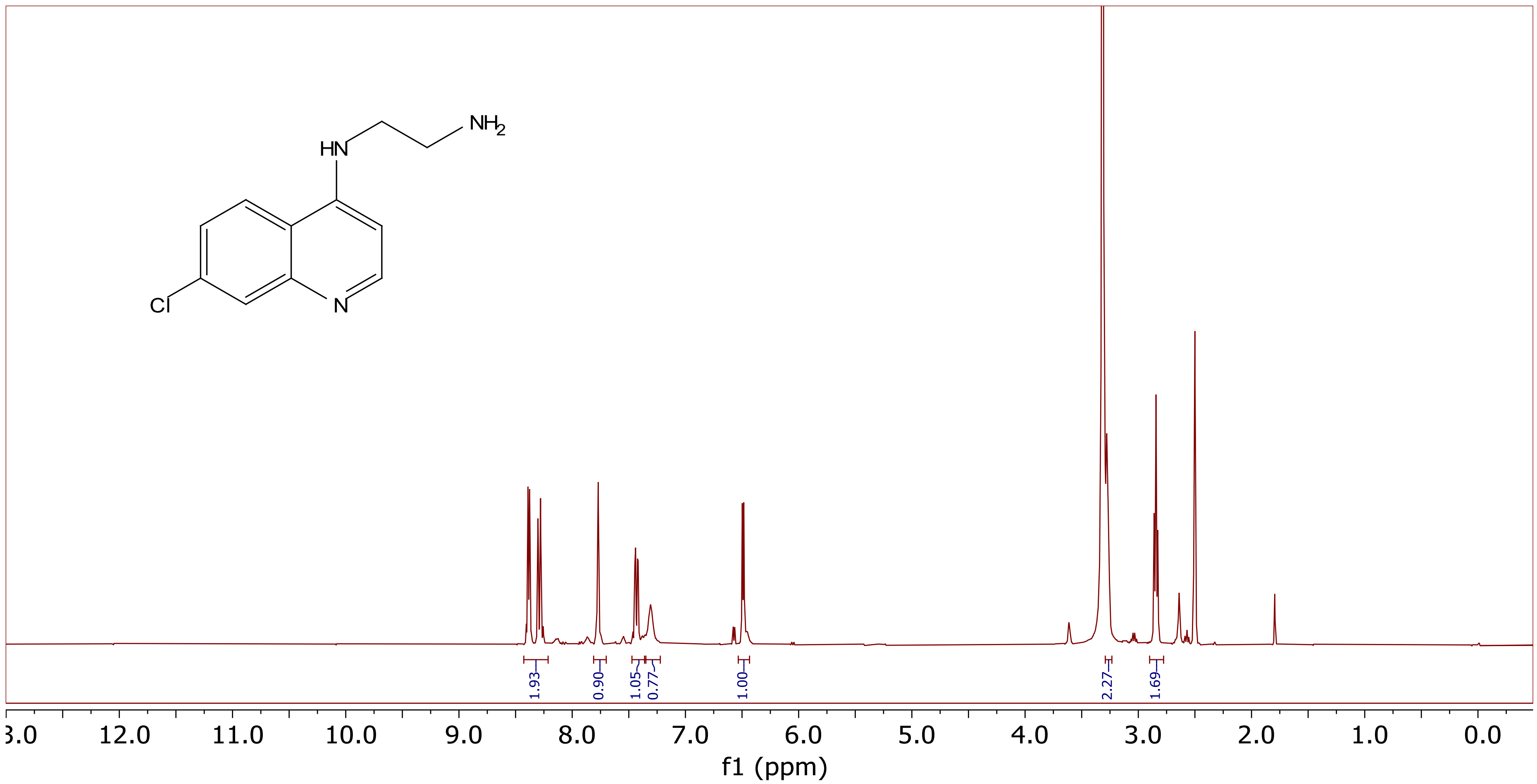

S1: ^{1}H NMR spectrum of *N*-(7-chloroquinolin-4-yl)ethane-1,2-diamine **2**

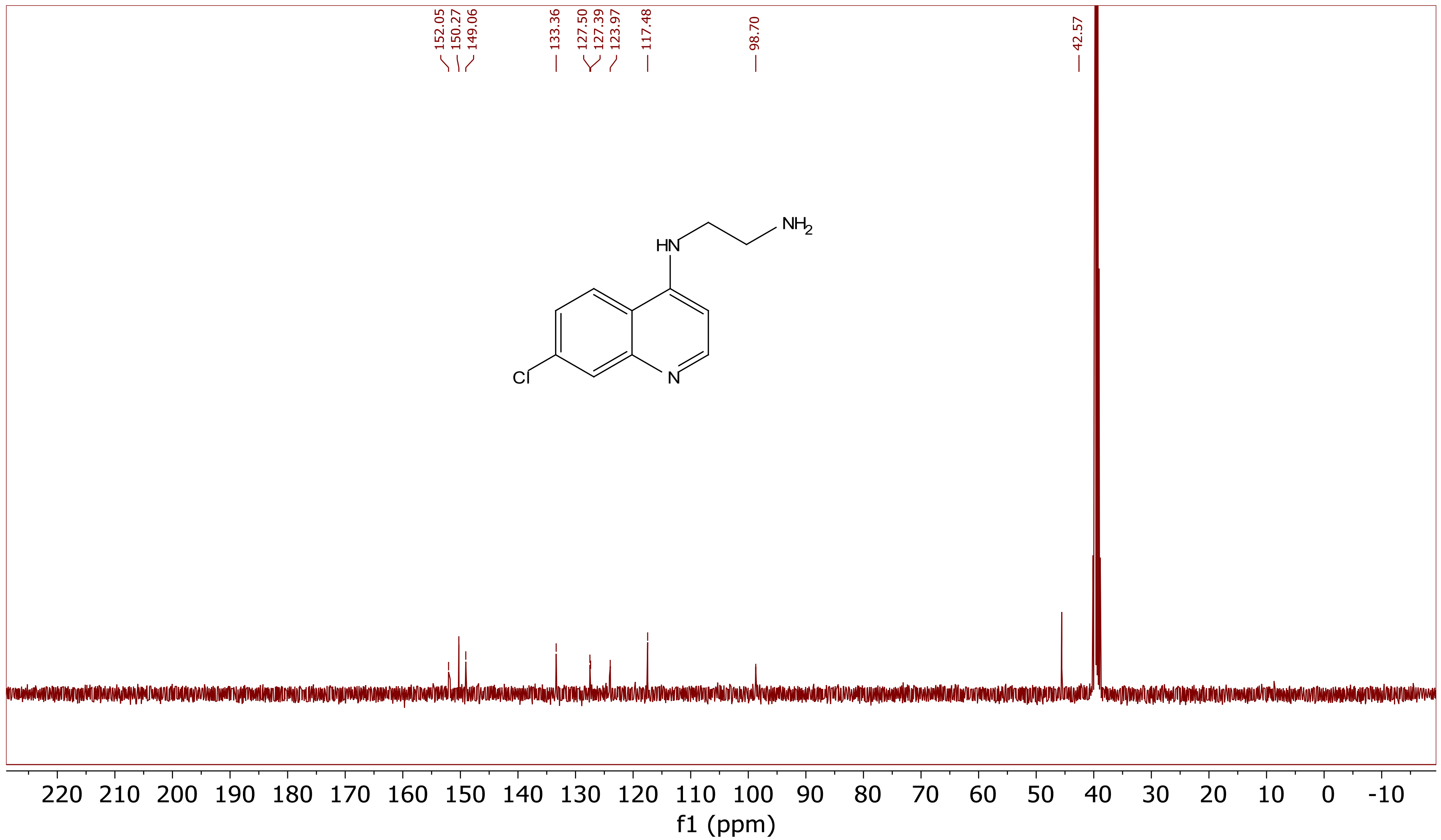

S2: ^{13}C NMR spectrum of N-(7-chloroquinolin-4-yl)ethane-1,2-diamine **2**

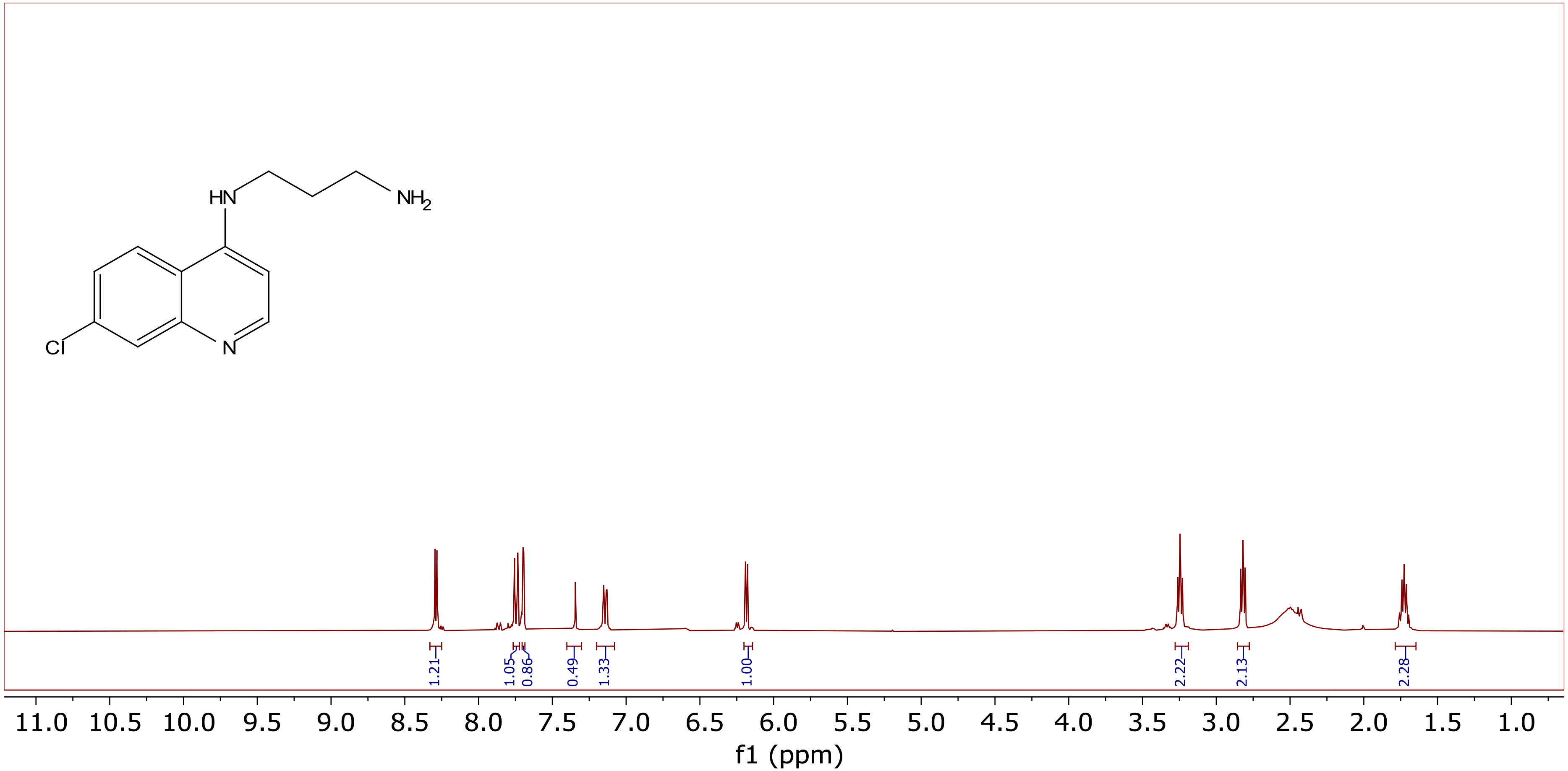

S3: ^{1}H NMR spectrum of N-(7-chloroquinolin-4-yl)propane-1,3-diamine **3**

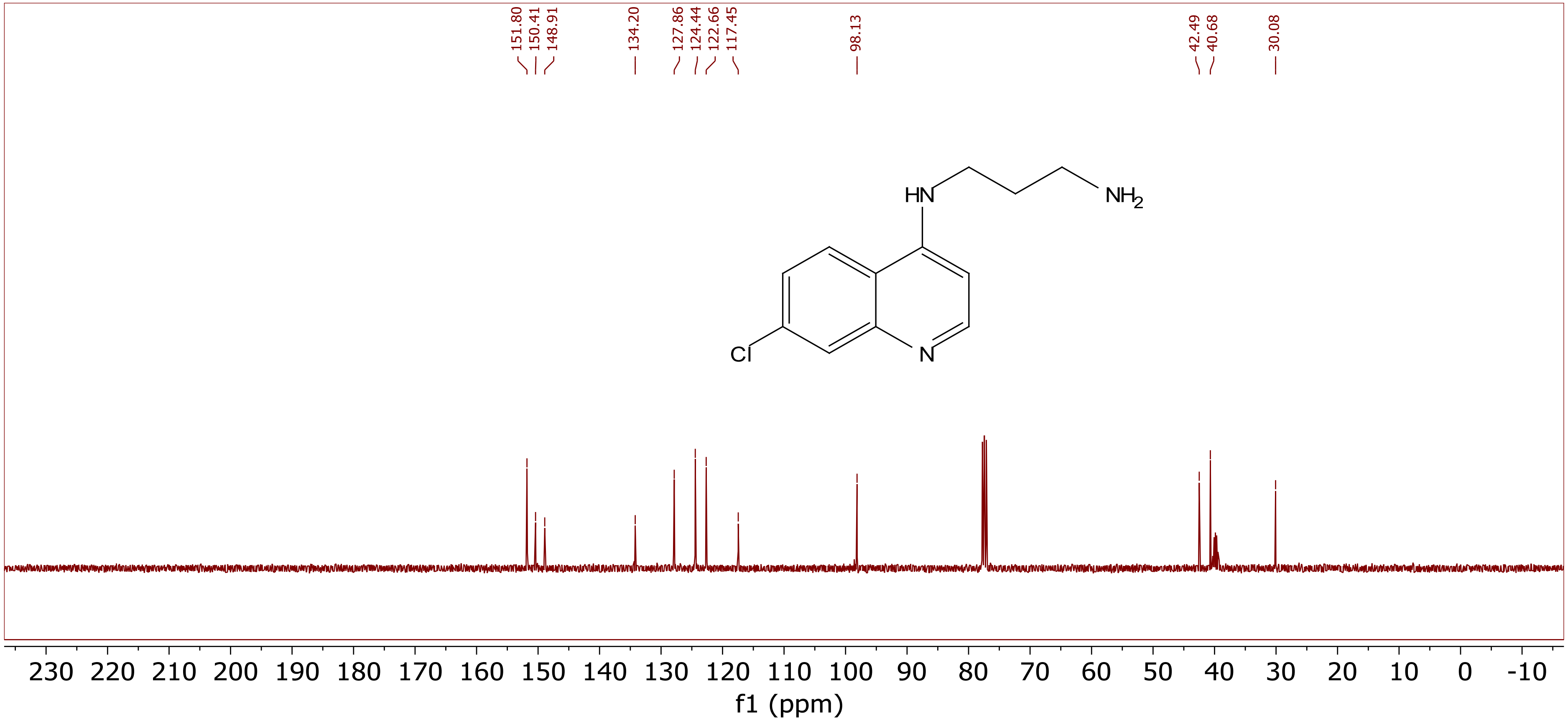

S4: ^{13}C NMR spectrum of N-(7-chloroquinolin-4-yl)propane-1,3-diamine **3**

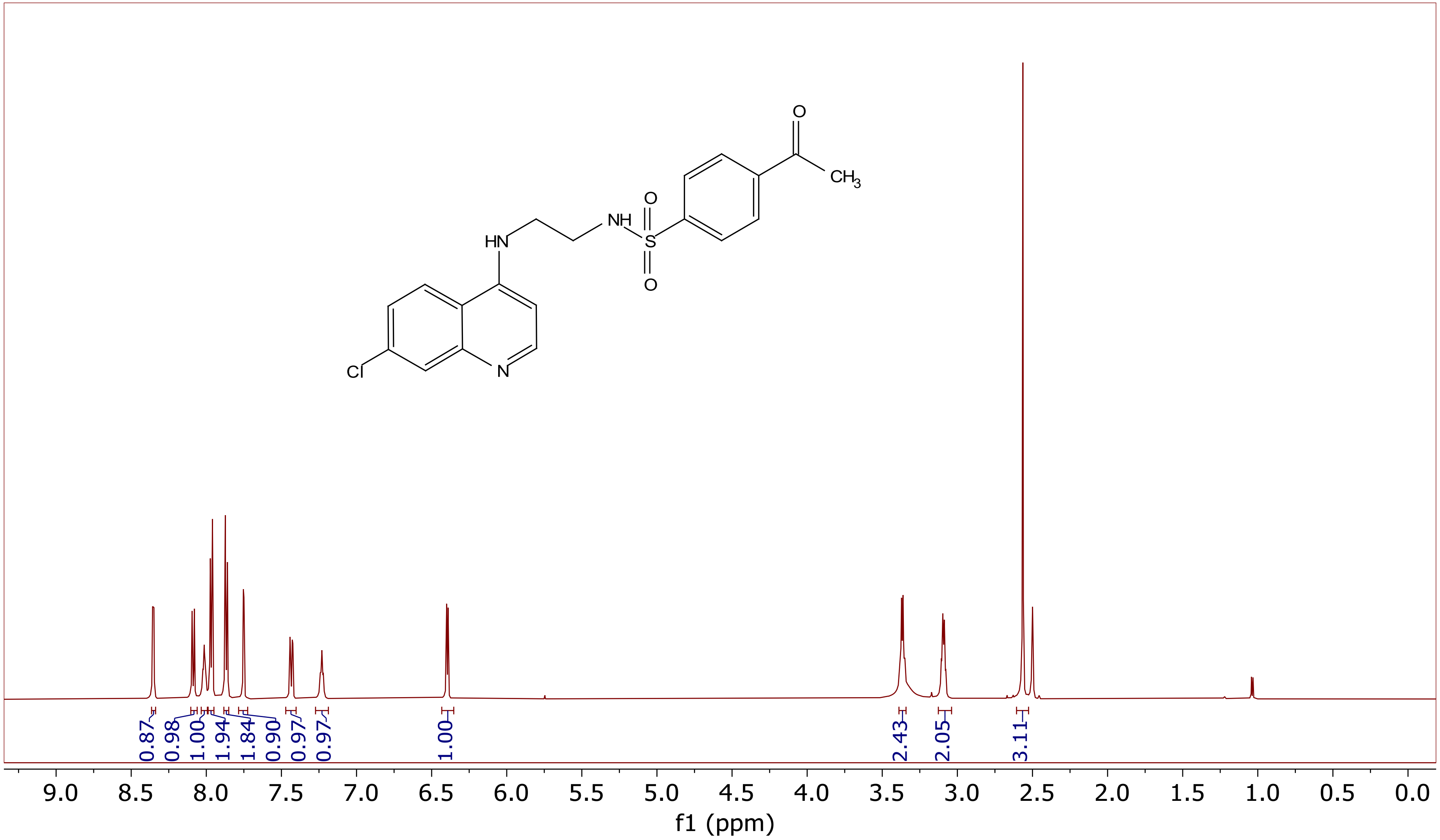

S5: ^{1}H NMR spectrum of 4-acetyl-*N*-{2-[(7-chloroquinolin-4-yl)amino]ethyl}benzenesulfonamide **4**

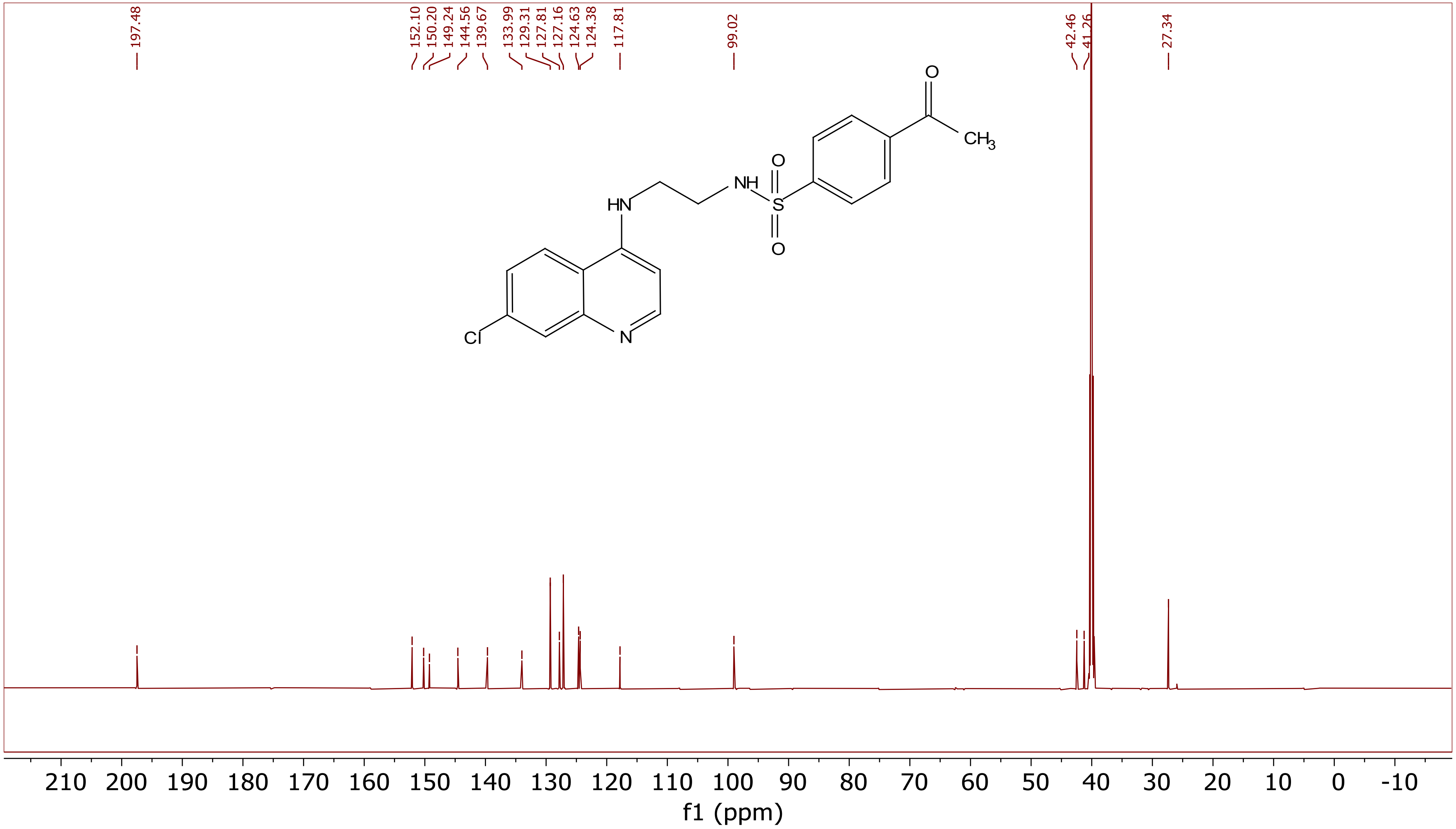

S6: ^{13}C NMR spectrum of 4-acetyl-*N*-{2-[(7-chloroquinolin-4-yl)amino]ethyl}benzenesulfonamide **4**

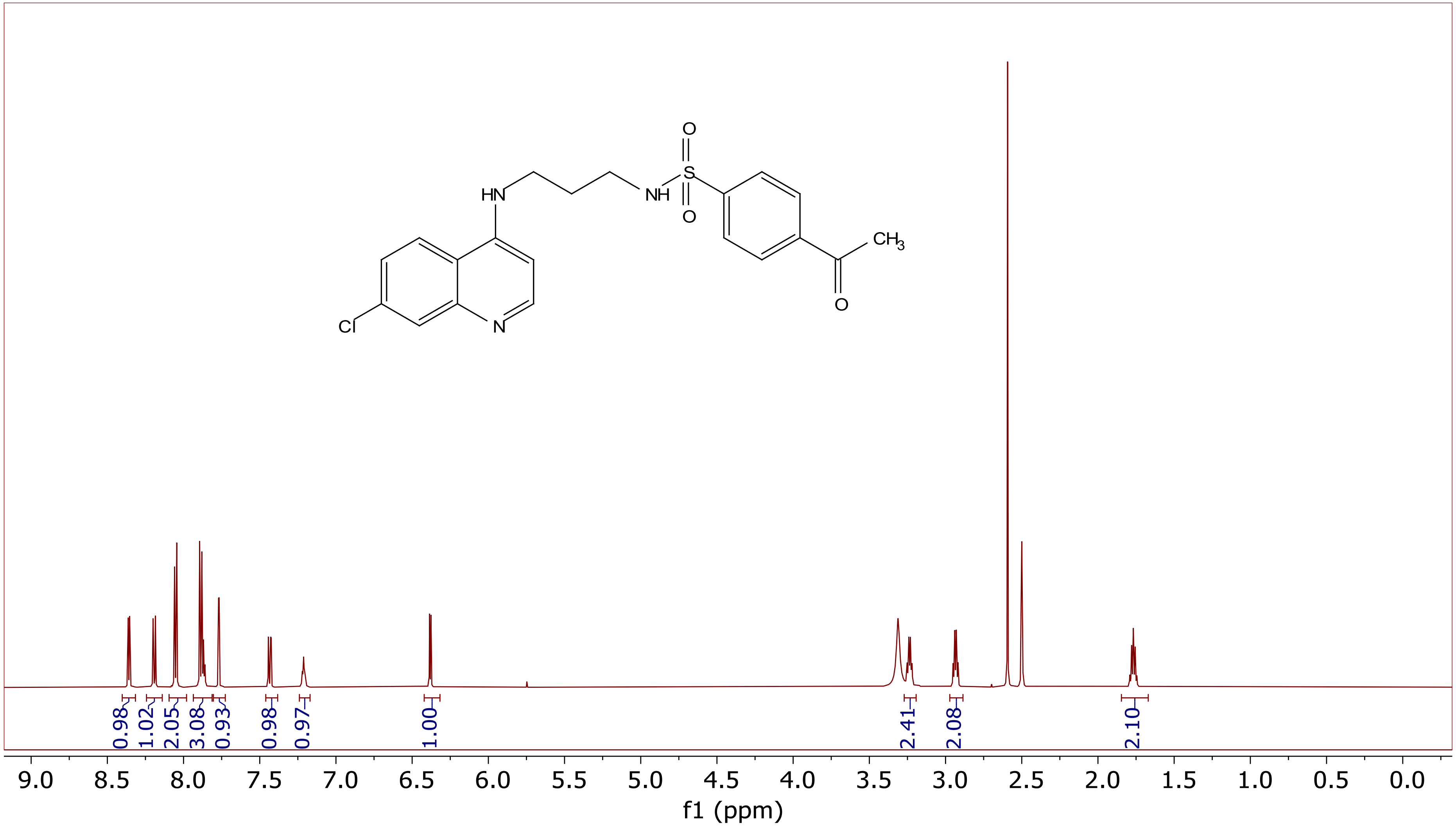

S7: ^{1}H NMR spectrum of 4-acetyl-*N*-{3-[(7-chloroquinolin-4-yl)amino]propyl}benzenesulfonamide **5**

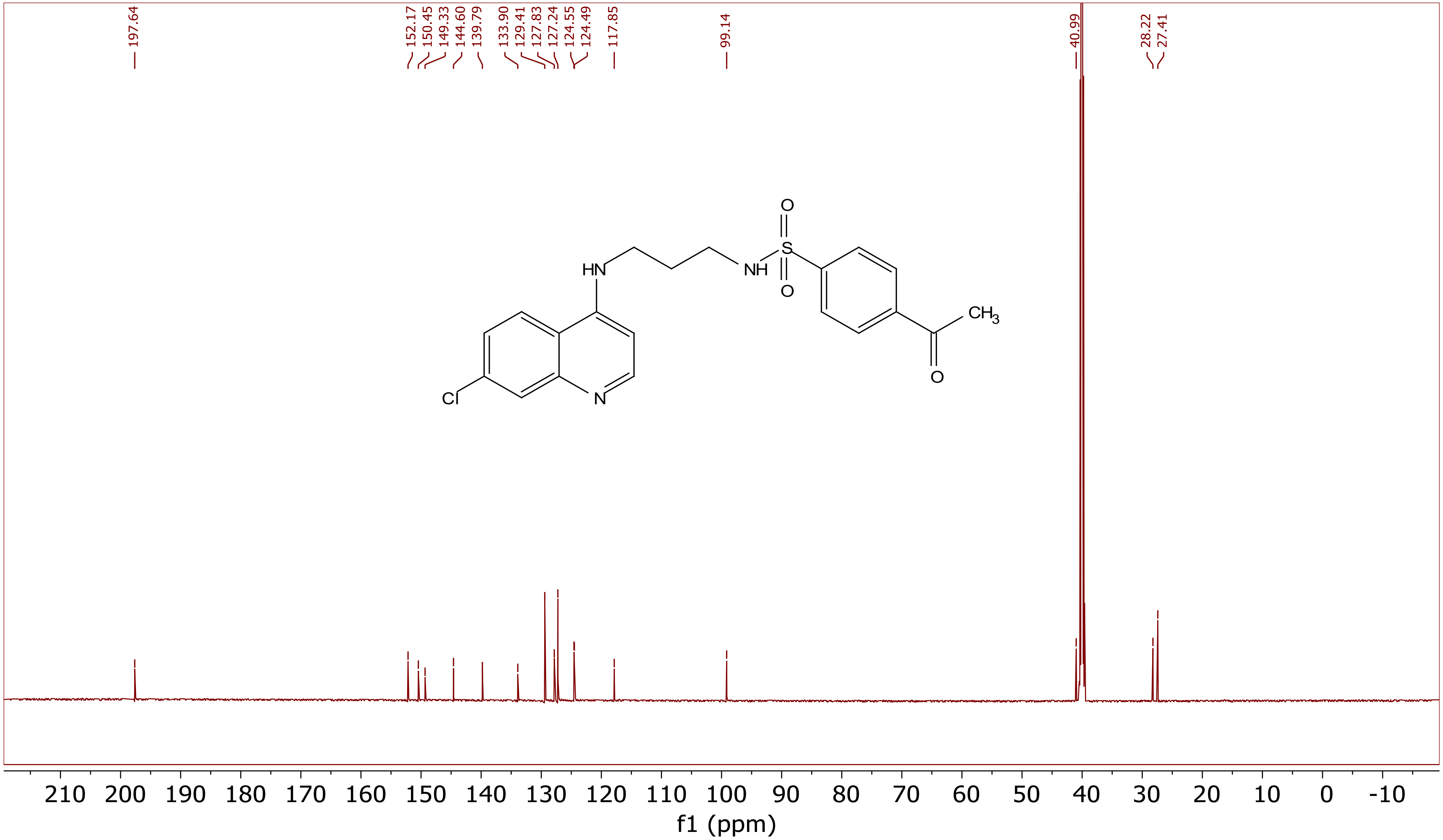

S8: ^{13}C NMR spectrum of 4-acetyl-N-{3-[(7-chloroquinolin-4-yl)amino]propyl}benzenesulfonamide **5**

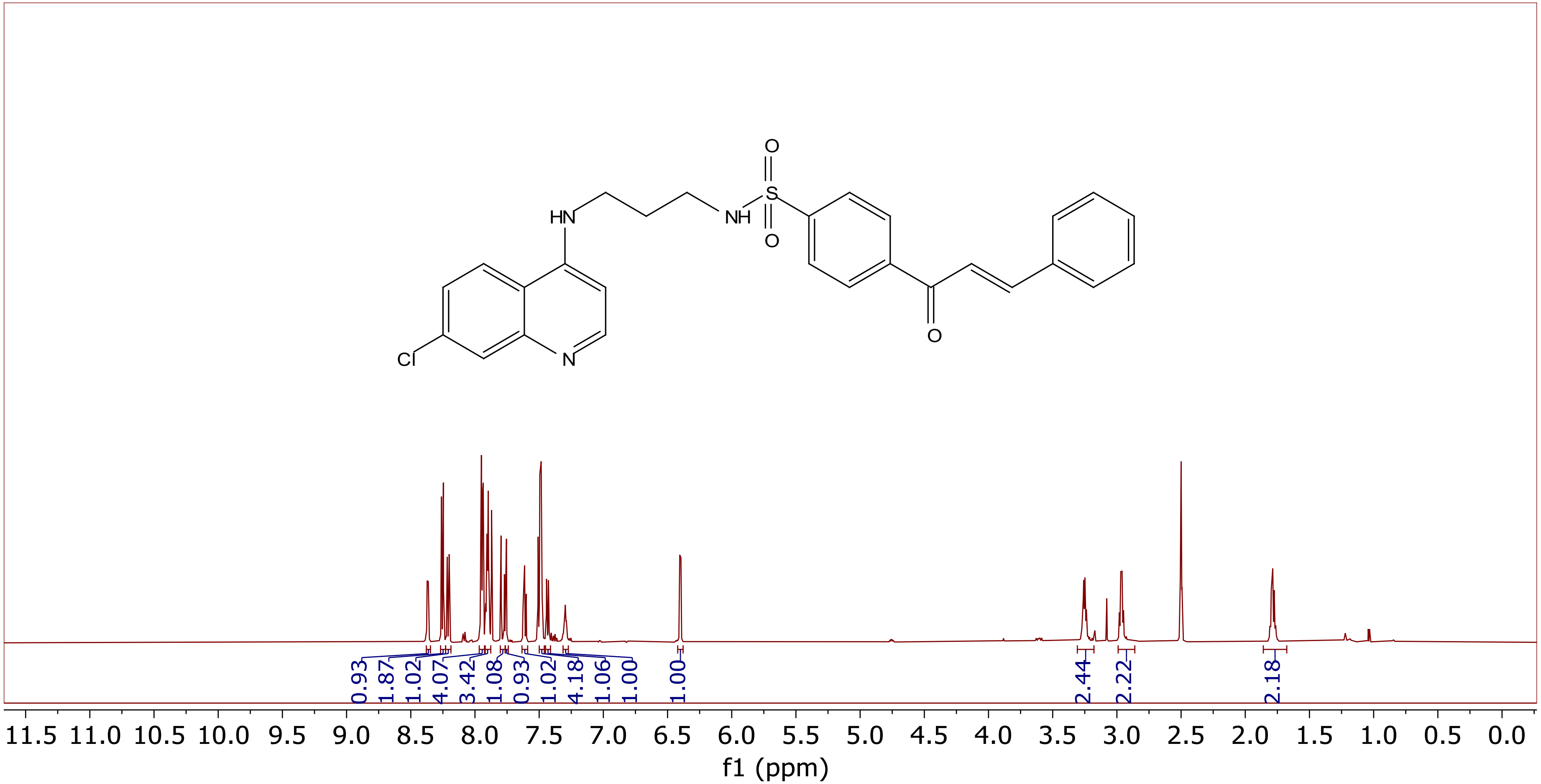

S9: ^{1}H NMR spectrum of *N*-(3-((7-Chloroquinolin-4-yl)amino)propyl)-4-cinnamoylbenzenesulfonamide **6**

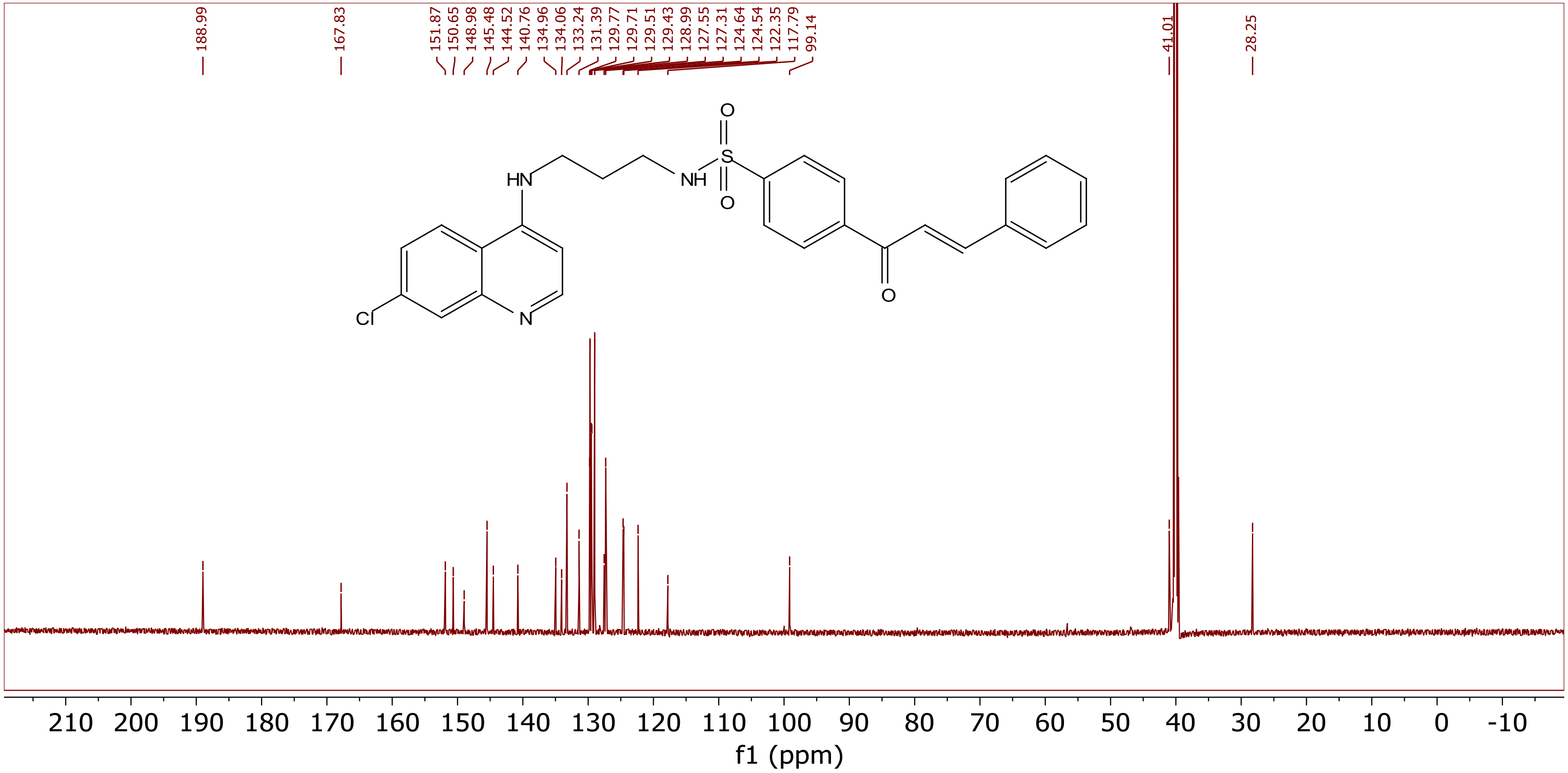

S10: ^{13}C NMR spectrum of N-(3-((7-Chloroquinolin-4-yl)amino)propyl)-4-cinnamoylbenzenesulfonamide **6**

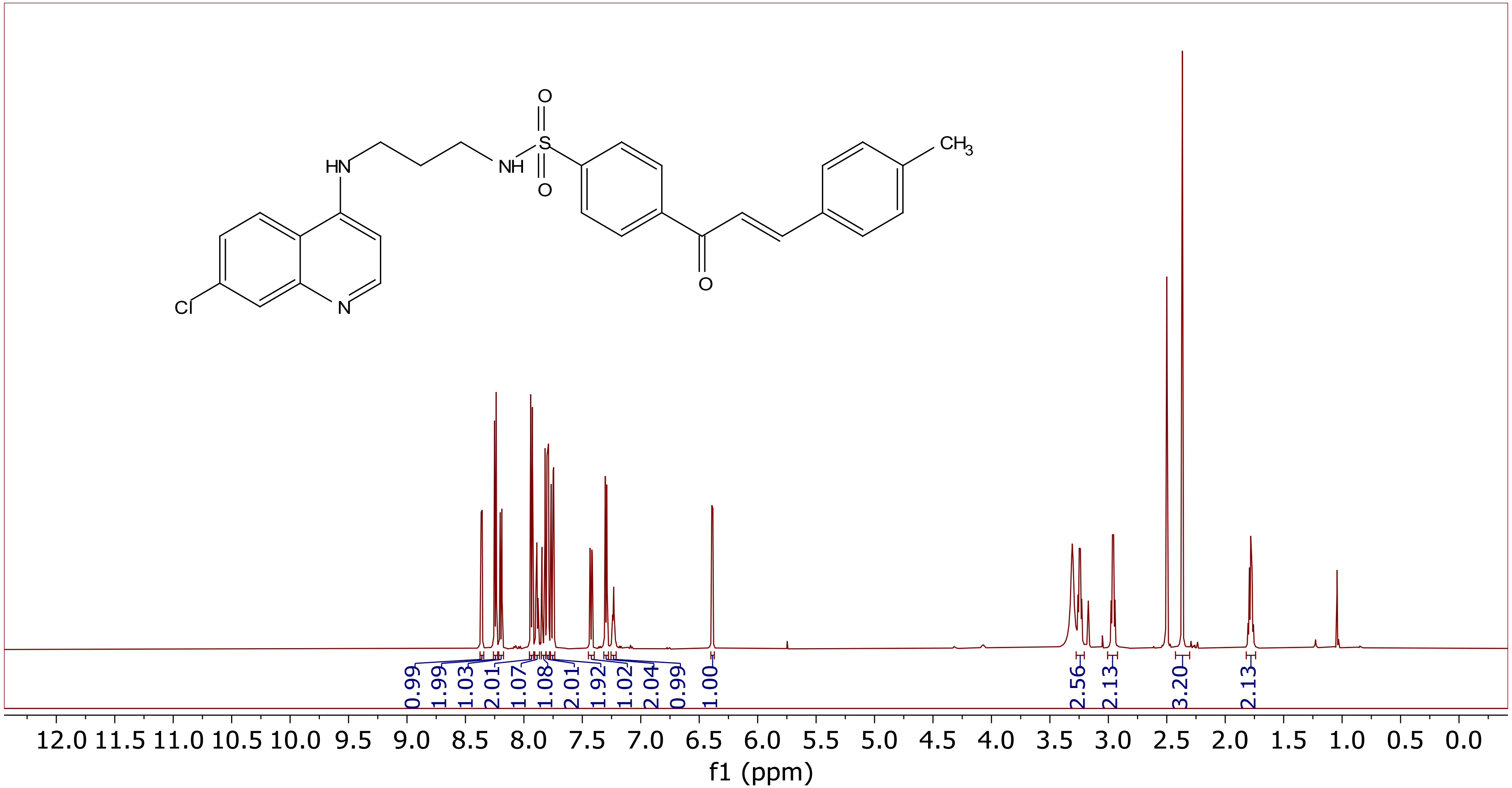

S11: ^{1}H NMR spectrum of (*E*)-*N*-(3-((7-Chloroquinolin-4-yl)amino)propyl)-4-(3-(p-tolyl)acryloyl)benzenesulfonamide **7**

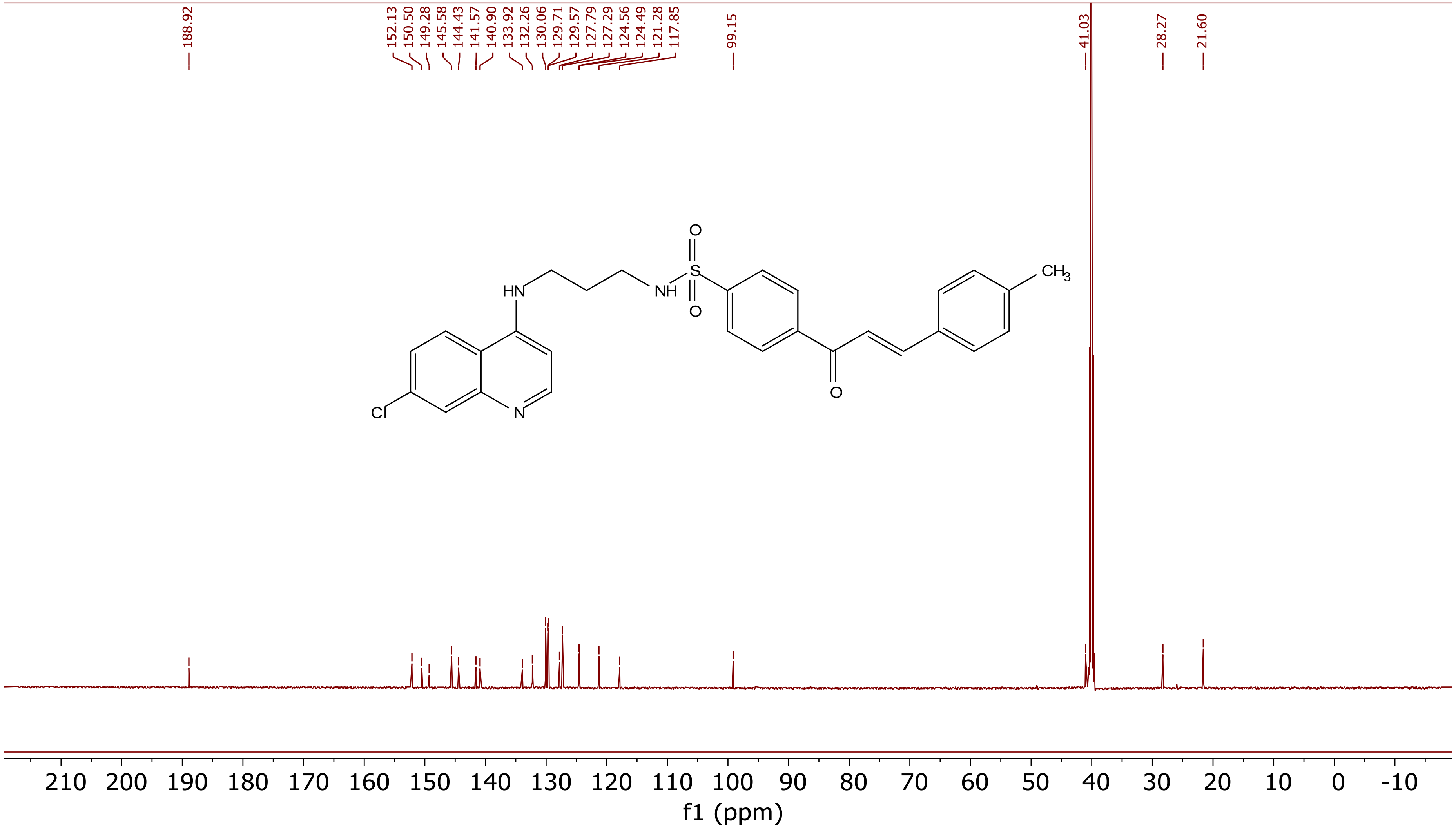

S12: ^{13}C NMR spectrum of (E)-N-(3-((7-Chloroquinolin-4-yl)amino)propyl)-4-(3-(p-tolyl)acryloyl)benzenesulfonamide **7**

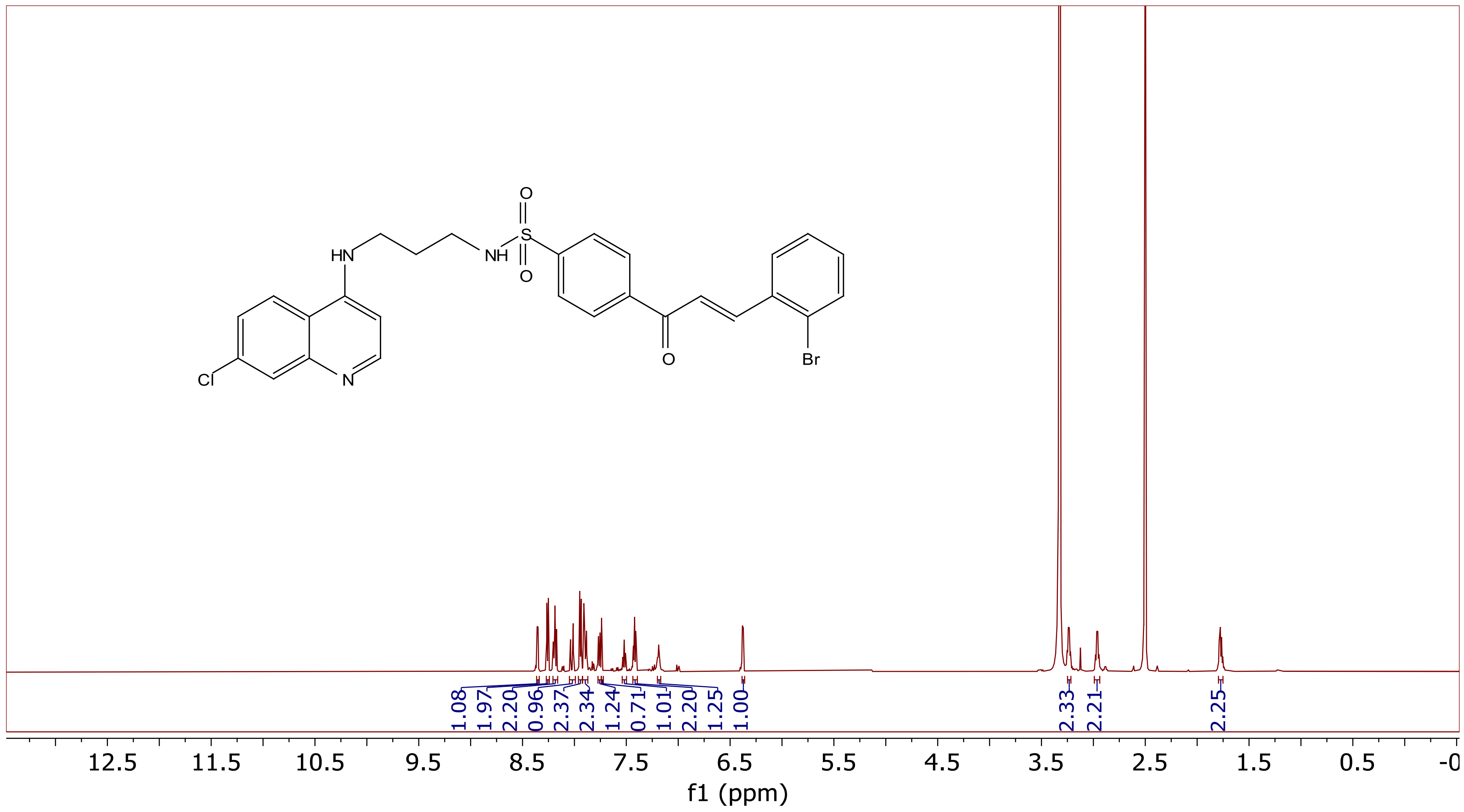

S13: ^{1}H NMR (*E*)-4-(3-(2-Bromophenyl)acryloyl)-*N*-(3-((7-chloroquinolin-4-yl)amino)propyl)benzenesulfonamide **8**

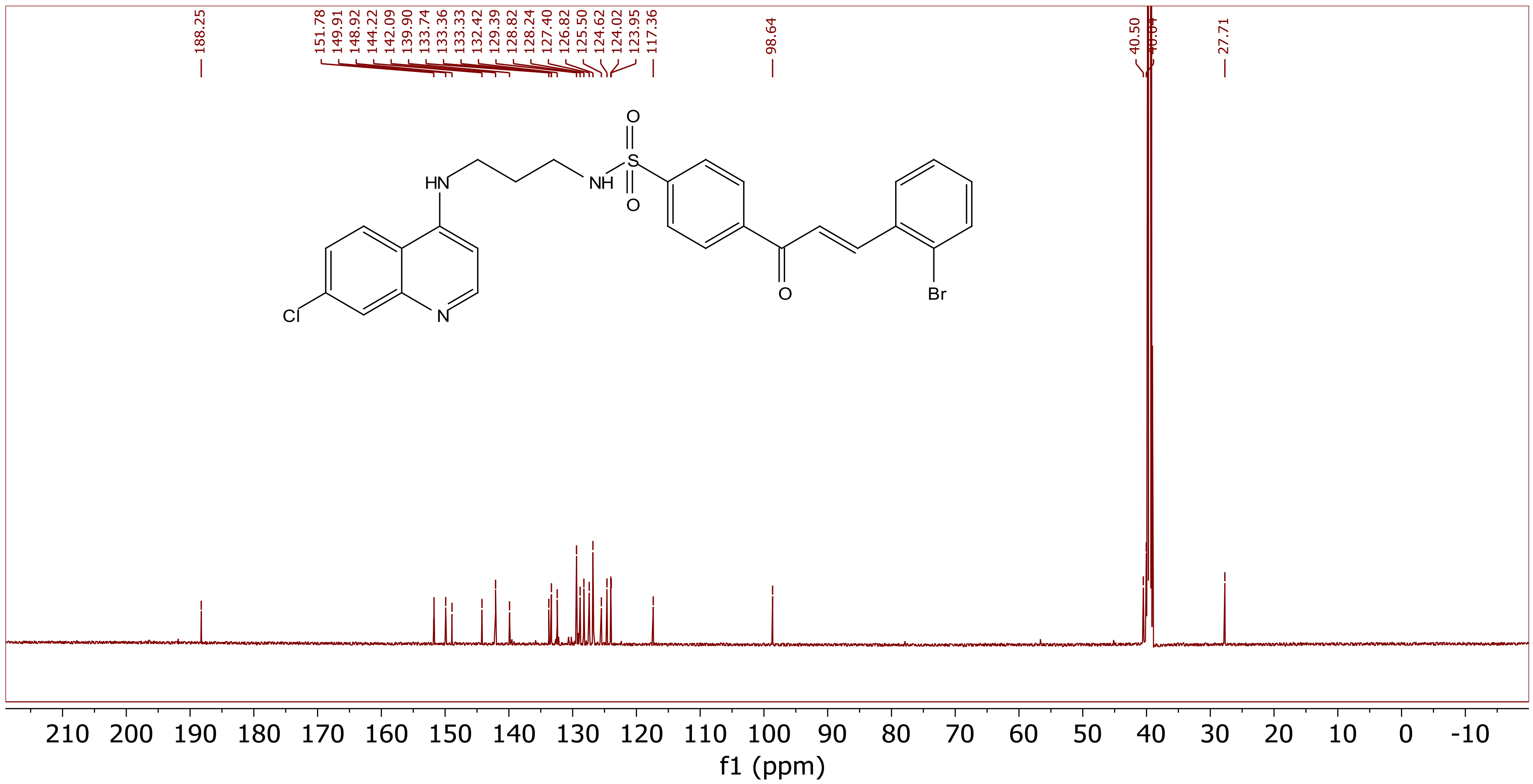

S14: ^{13}C NMR (*E*)-4-(3-(2-Bromophenyl)acryloyl)-*N*-(3-((7-chloroquinolin-4-yl)amino)propyl)benzenesulfonamide **8**

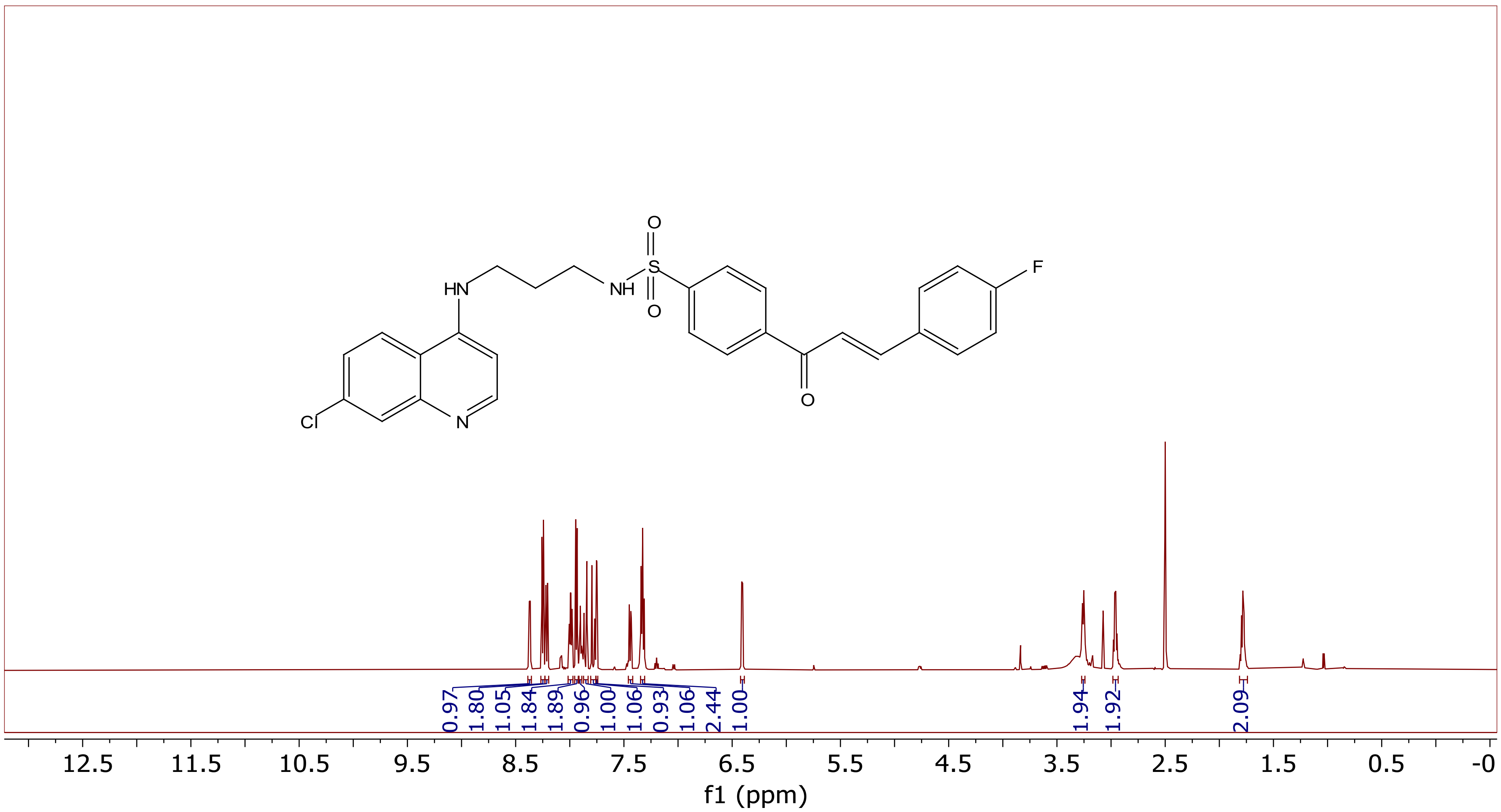

S15: ^{1}H NMR spectrum of (*E*)-*N*-(3-((7-Chloroquinolin-4-yl)amino)propyl)-4-(3-(4-fluorophenyl)acryloyl)benzenesulfonamide **9**

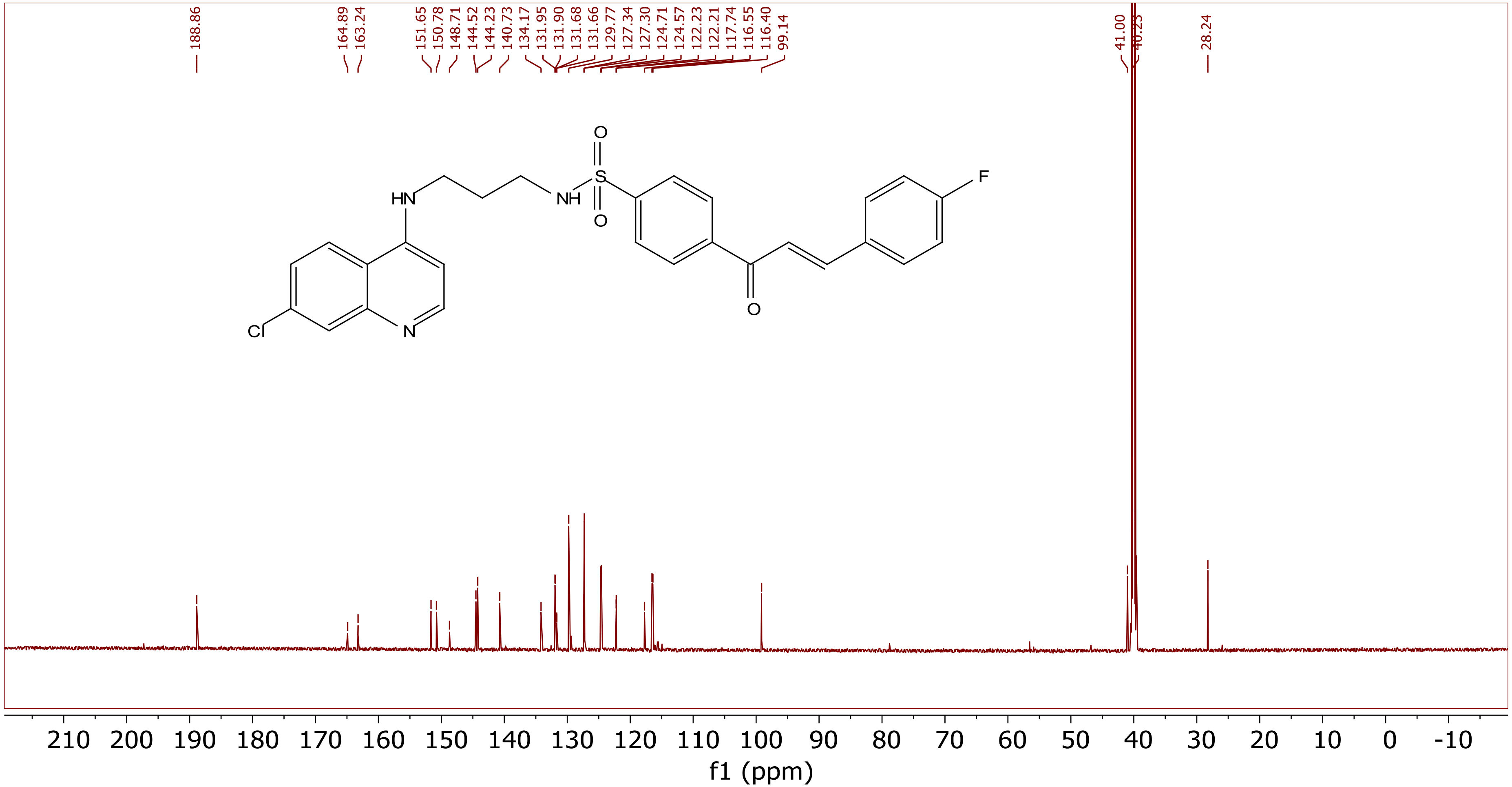

S16: ^{13}C NMR spectrum of (*E*)-*N*-(3-((7-Chloroquinolin-4-yl)amino)propyl)-4-(3-(4-fluorophenyl)acryloyl)benzenesulfonamide **9**

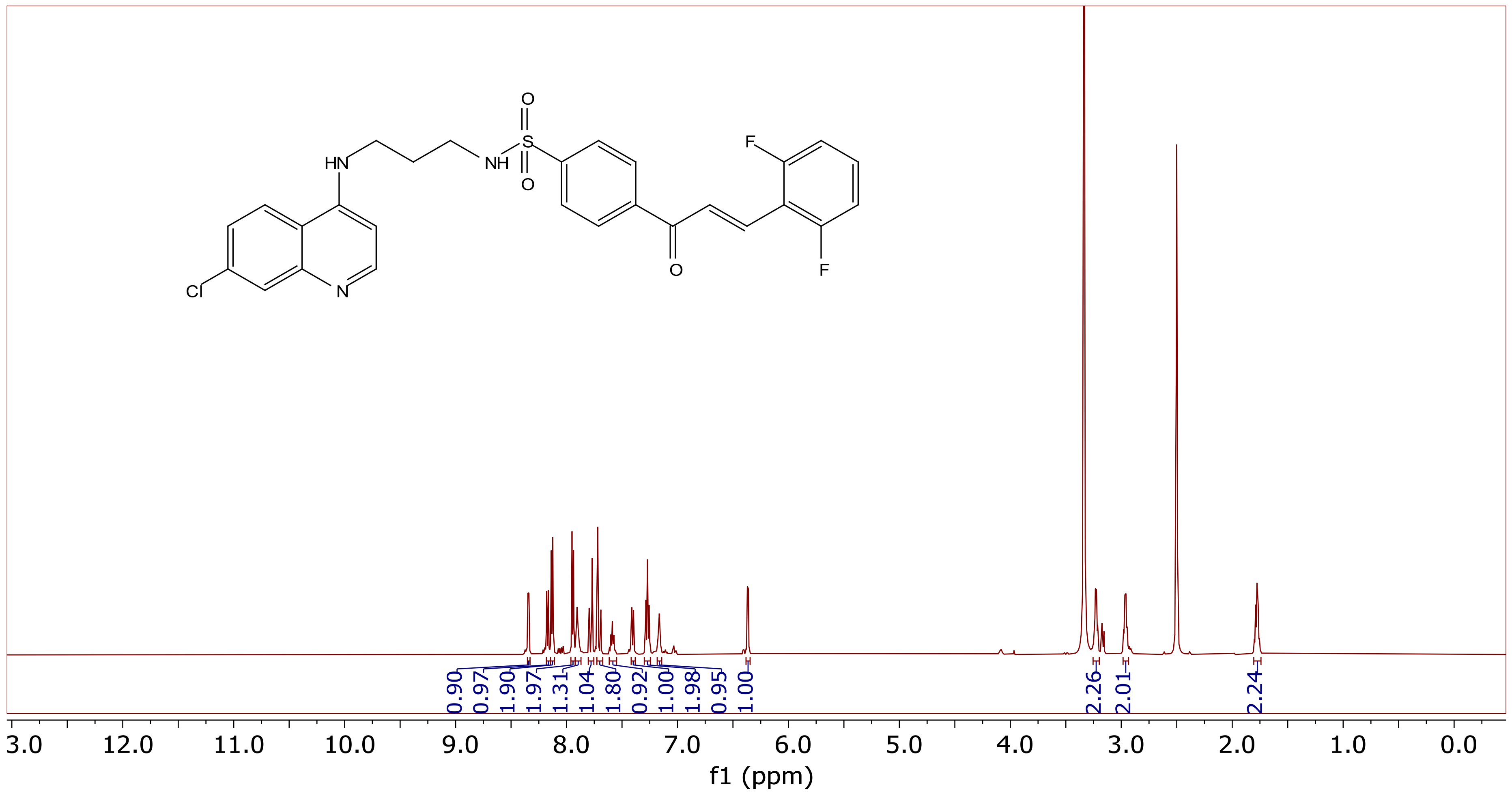

S17: ^{1}H NMR spectrum of (E)-N-(3-((7-Chloroquinolin-4-yl)amino)propyl)-4-(3-(2,6-difluorophenyl)acryloyl)benzenesulfonamide **10**

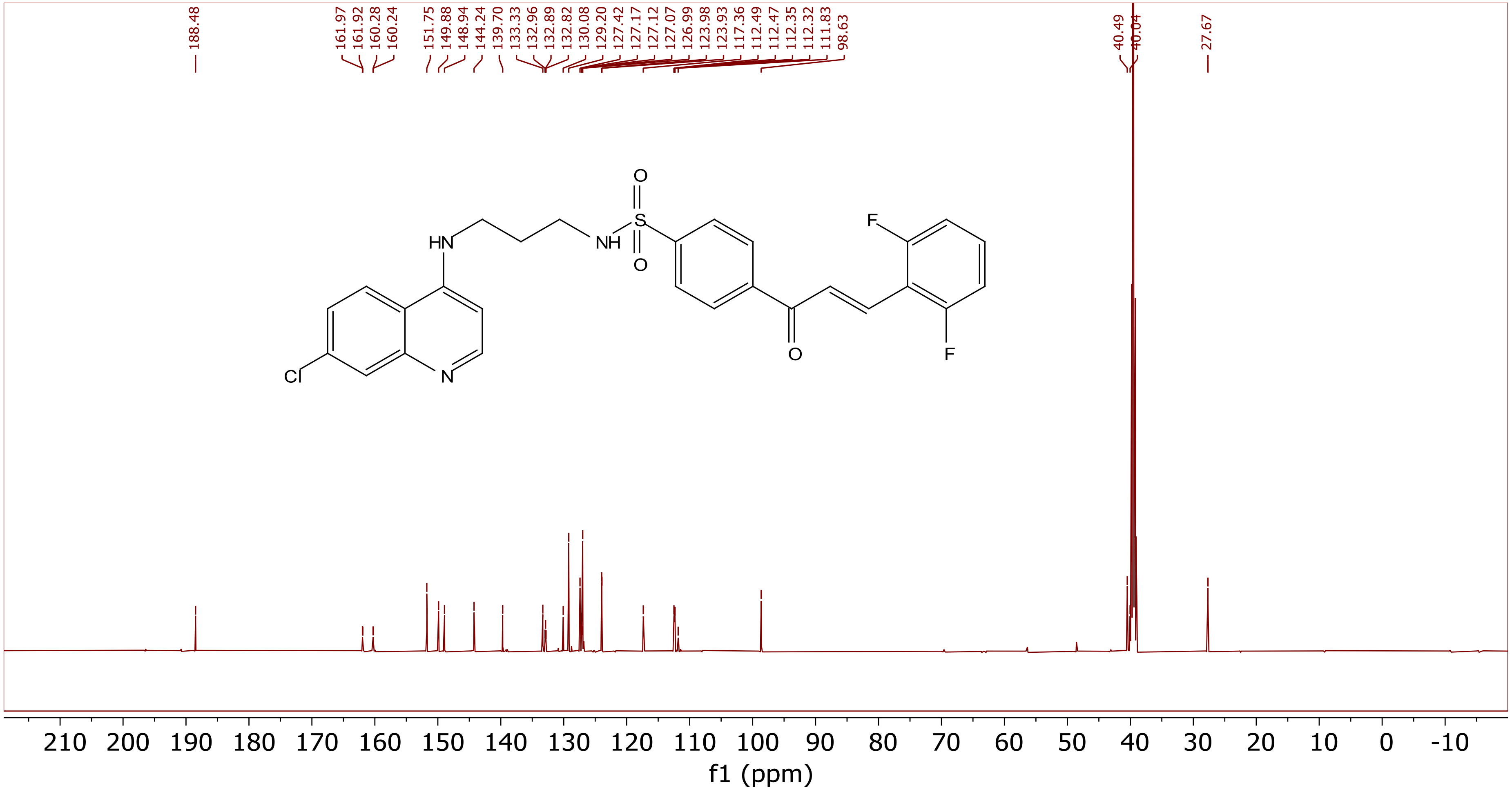

S18: ^{13}C NMR spectrum of (E)-N-(3-((7-Chloroquinolin-4-yl)amino)propyl)-4-(3-(2,6-difluorophenyl)acryloyl)benzenesulfonamide **10**

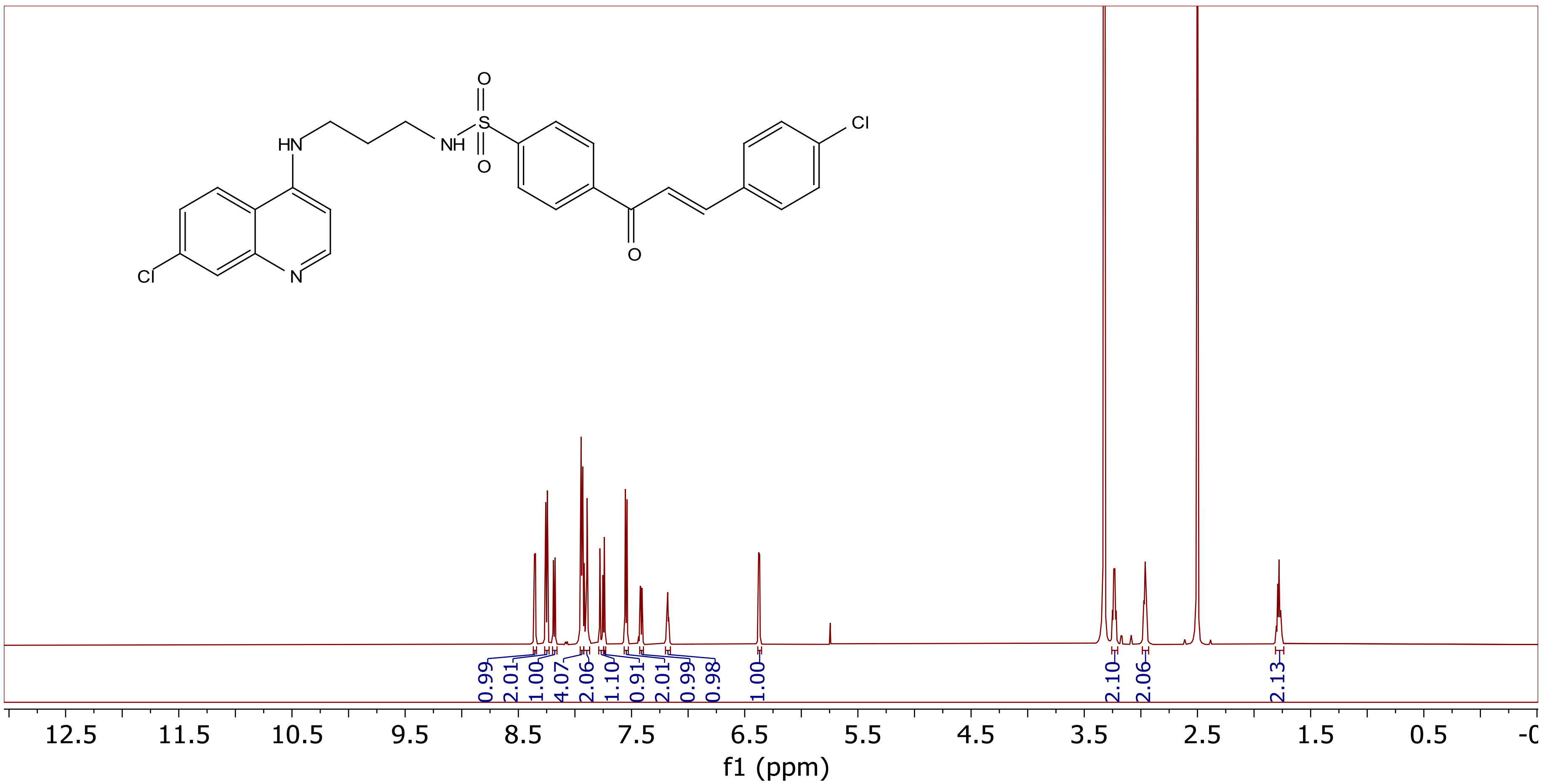

S19: ^{1}H NMR spectrum of (*E*)-4-(3-(4-Chlorophenyl)acryloyl)-*N*-(3-((7-chloroquinolin-4-yl)amino)propyl)benzenesulfonamide **11**

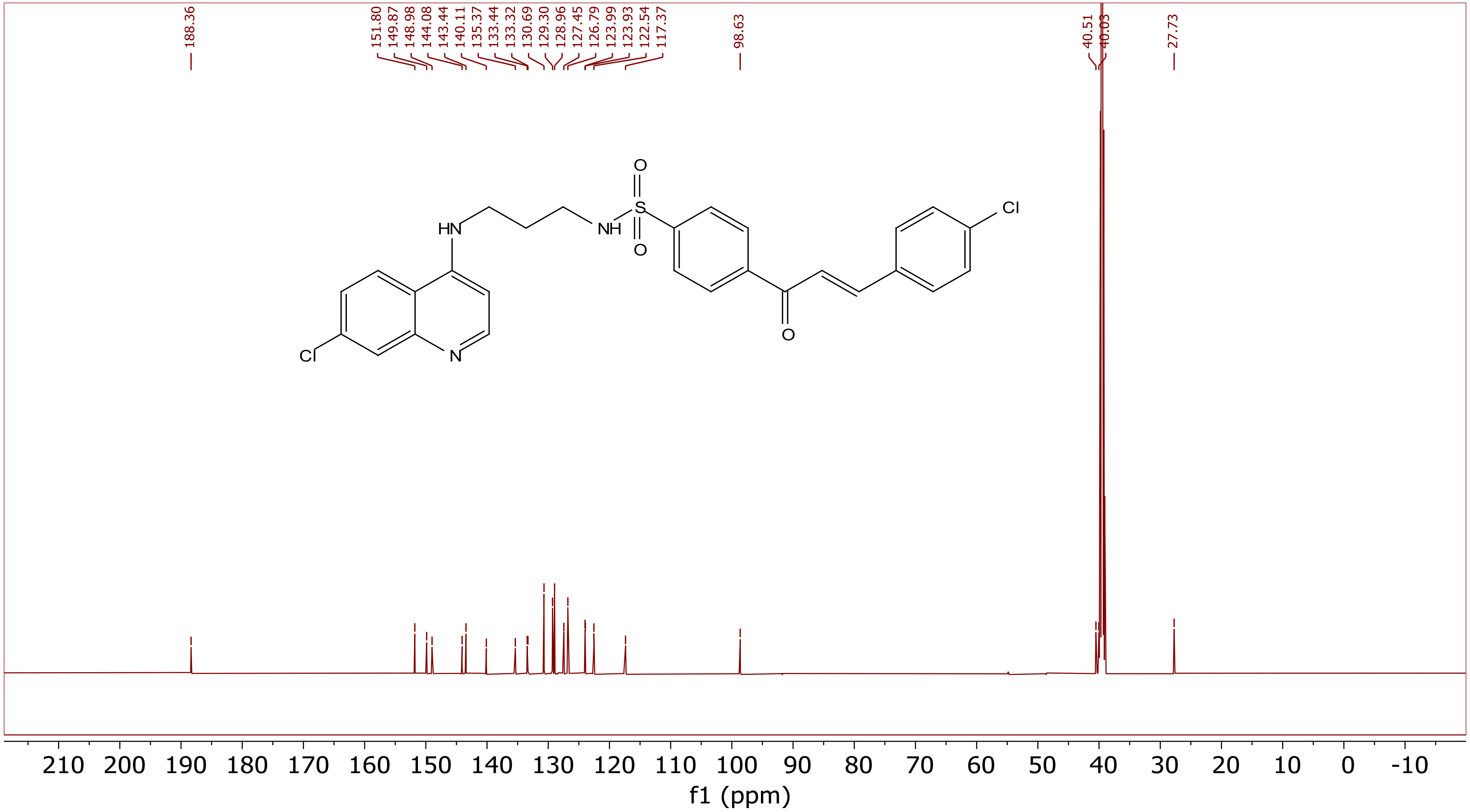

S20: ^{13}C NMR spectrum of (E)-4-(3-(4-Chlorophenyl)acryloyl)-N-(3-((7-chloroquinolin-4-yl)amino)propyl)benzenesulfonamide **11**

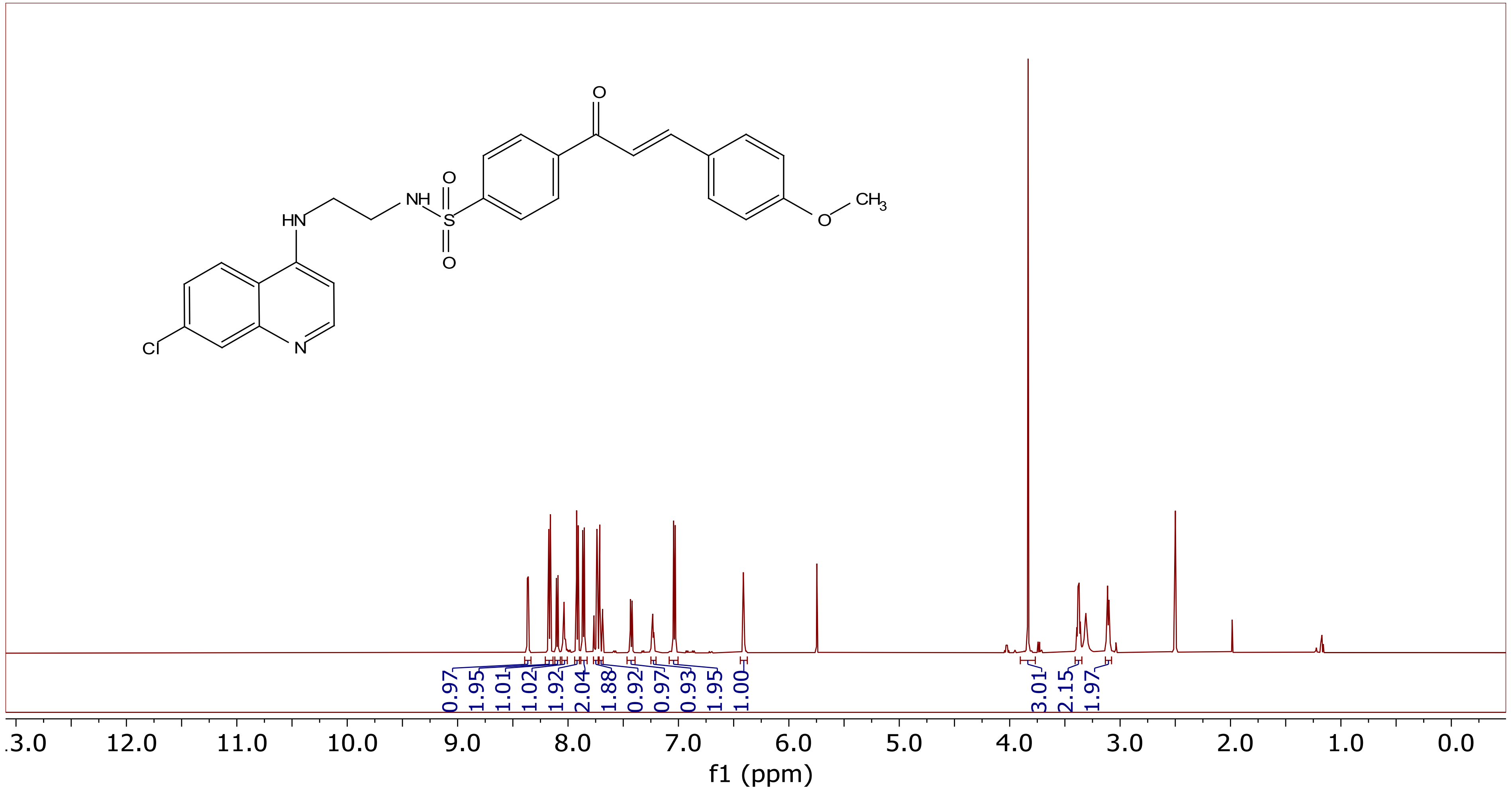

S21: ^{1}H NMR spectrum of (*E*)-*N*-(3-((7-Chloroquinolin-4-yl)amino)propyl)-4-(3-(4-methoxyphenyl)acryloyl)benzenesulfonamide **12**

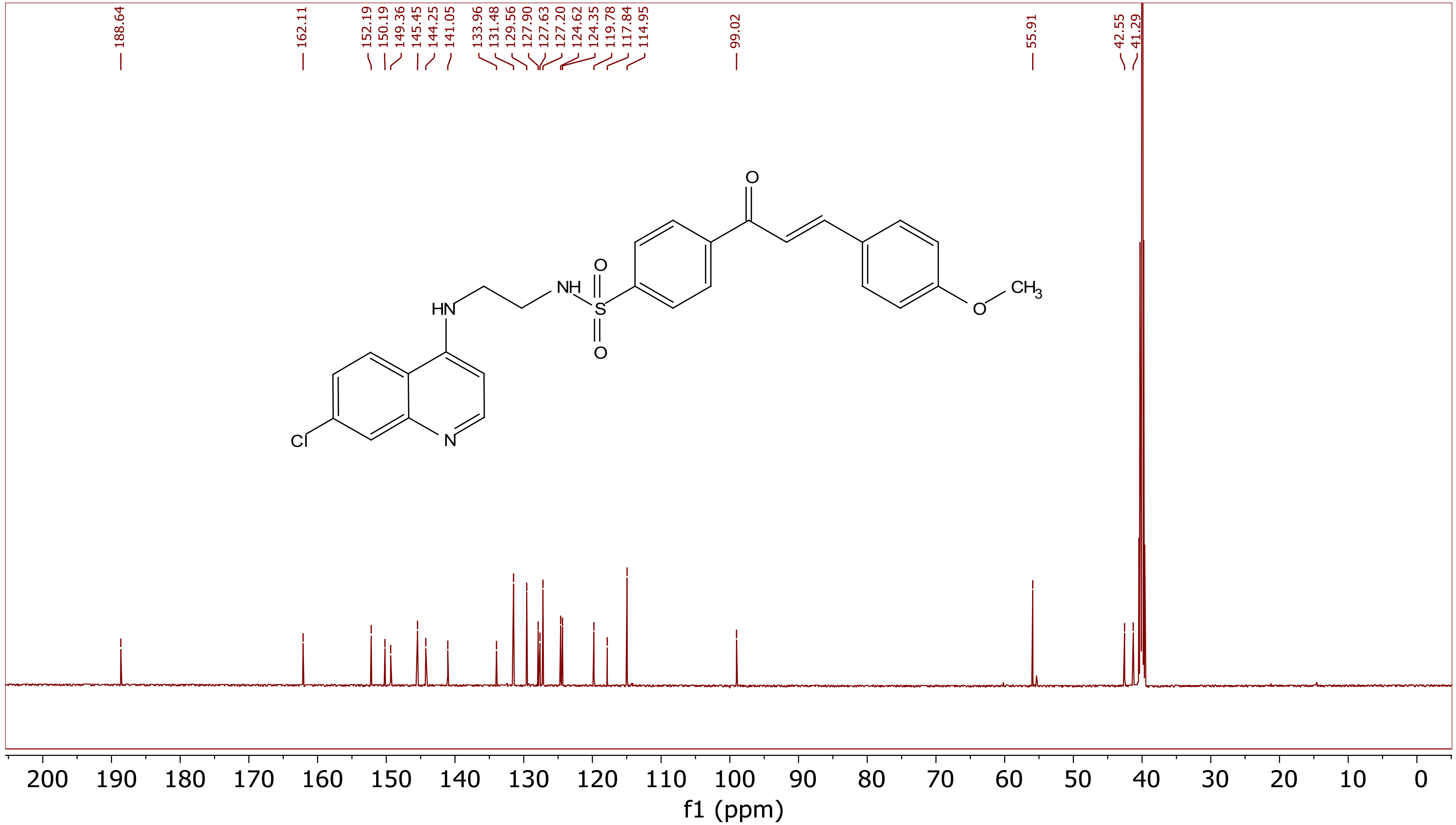

S22: ^{13}C NMR spectrum of (*E*)-*N*-(3-((7-Chloroquinolin-4-yl)amino)propyl)-4-(3-(4-methoxyphenyl)acryloyl)benzenesulfonamide **12**

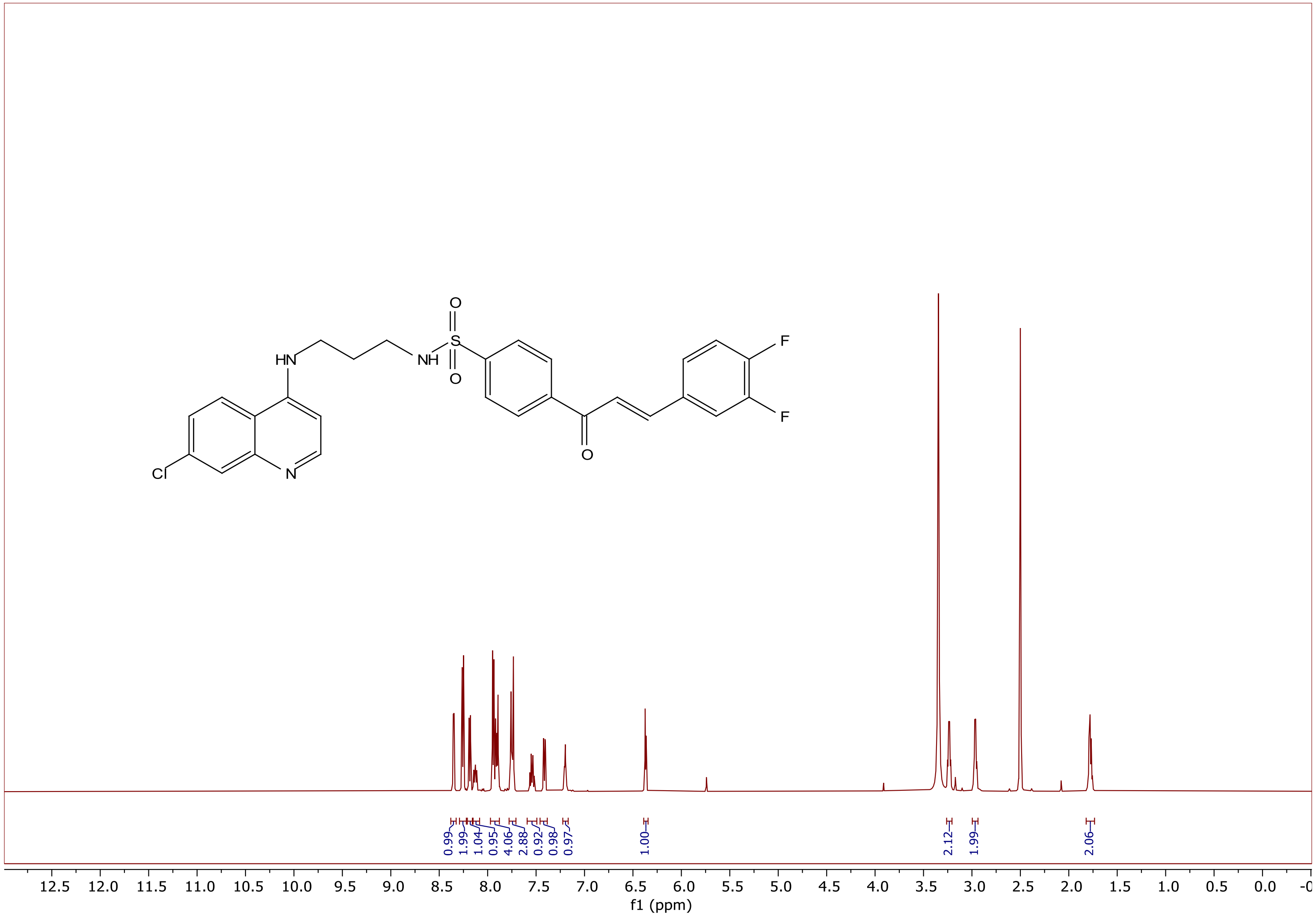

S23: ^{1}H NMR spectrum of (*E*)-*N*-(3-((7-Chloroquinolin-4-yl)amino)propyl)-4-(3-(3,4-difluorophenyl)acryloyl)benzenesulfonamide **13**

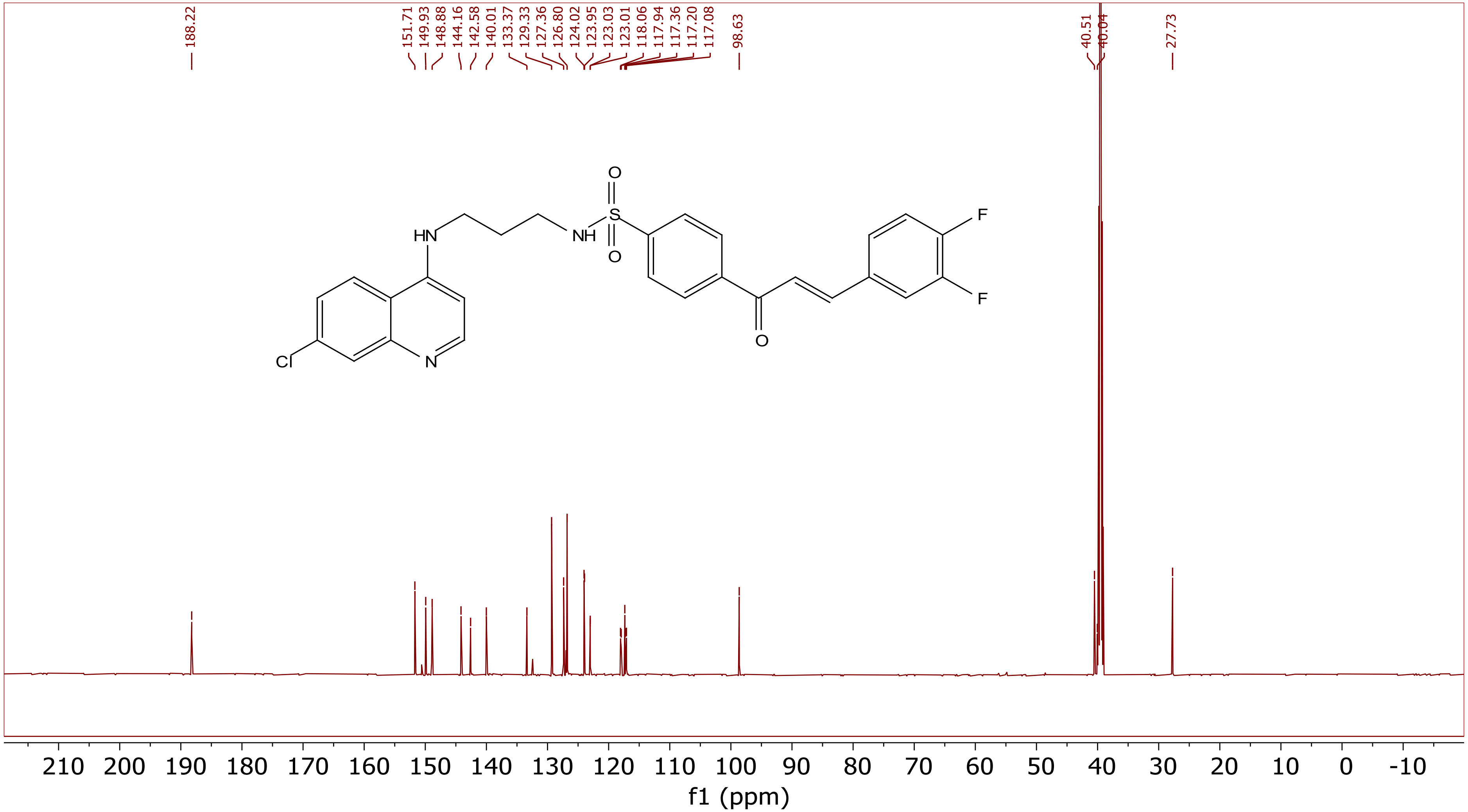

S24: ^{13}C NMR spectrum of (*E*)-*N*-(3-((7-Chloroquinolin-4-yl)amino)propyl)-4-(3-(3,4-difluorophenyl)acryloyl)benzenesulfonamide **13**

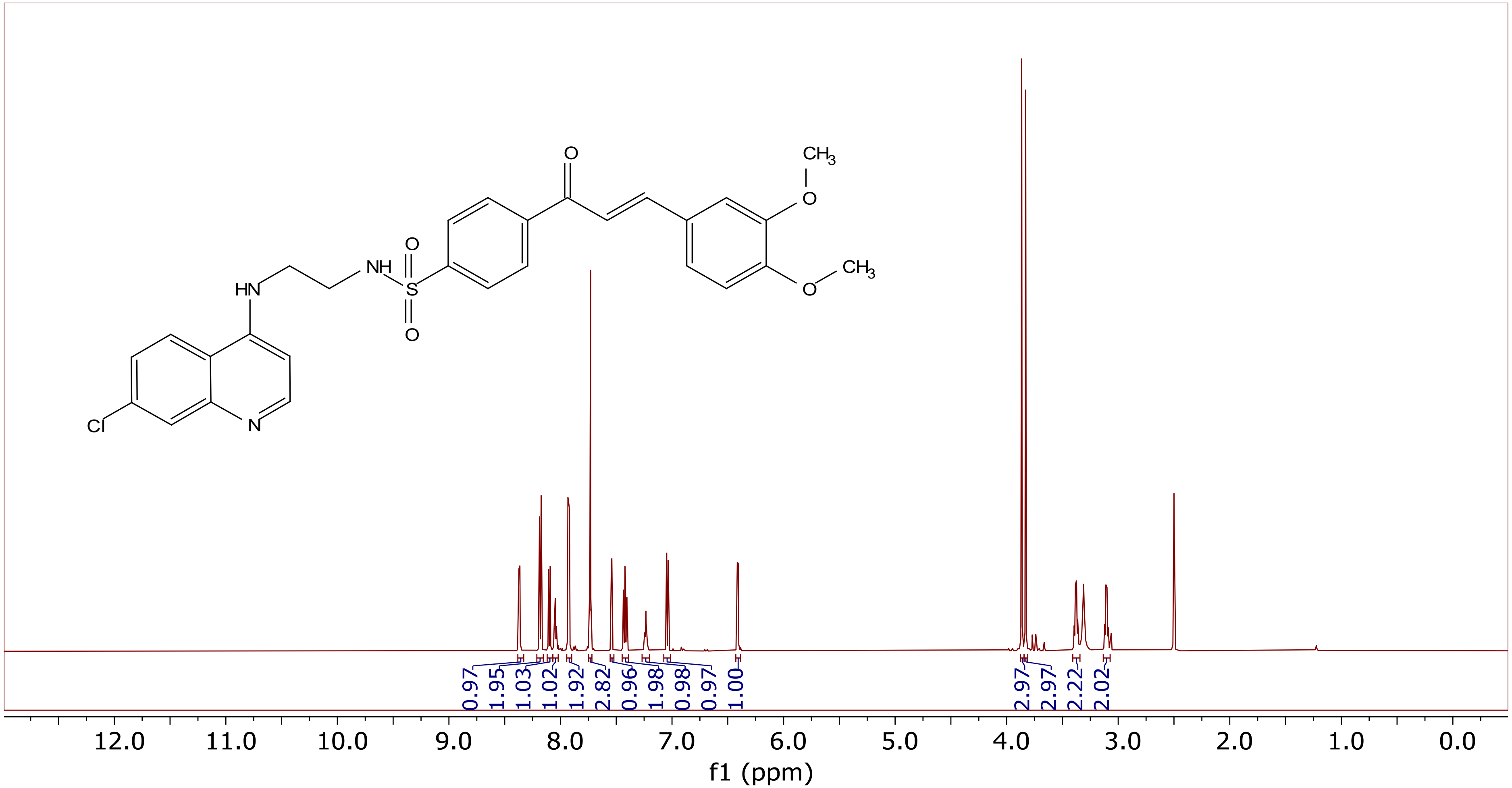

S25: ^{1}H NMR spectrum of (E)-N-(3-((7-Chloroquinolin-4-yl)amino)propyl)-4-(3-(3,4-dimethoxyphenyl)acryloyl)benzenesulfonamide **14**

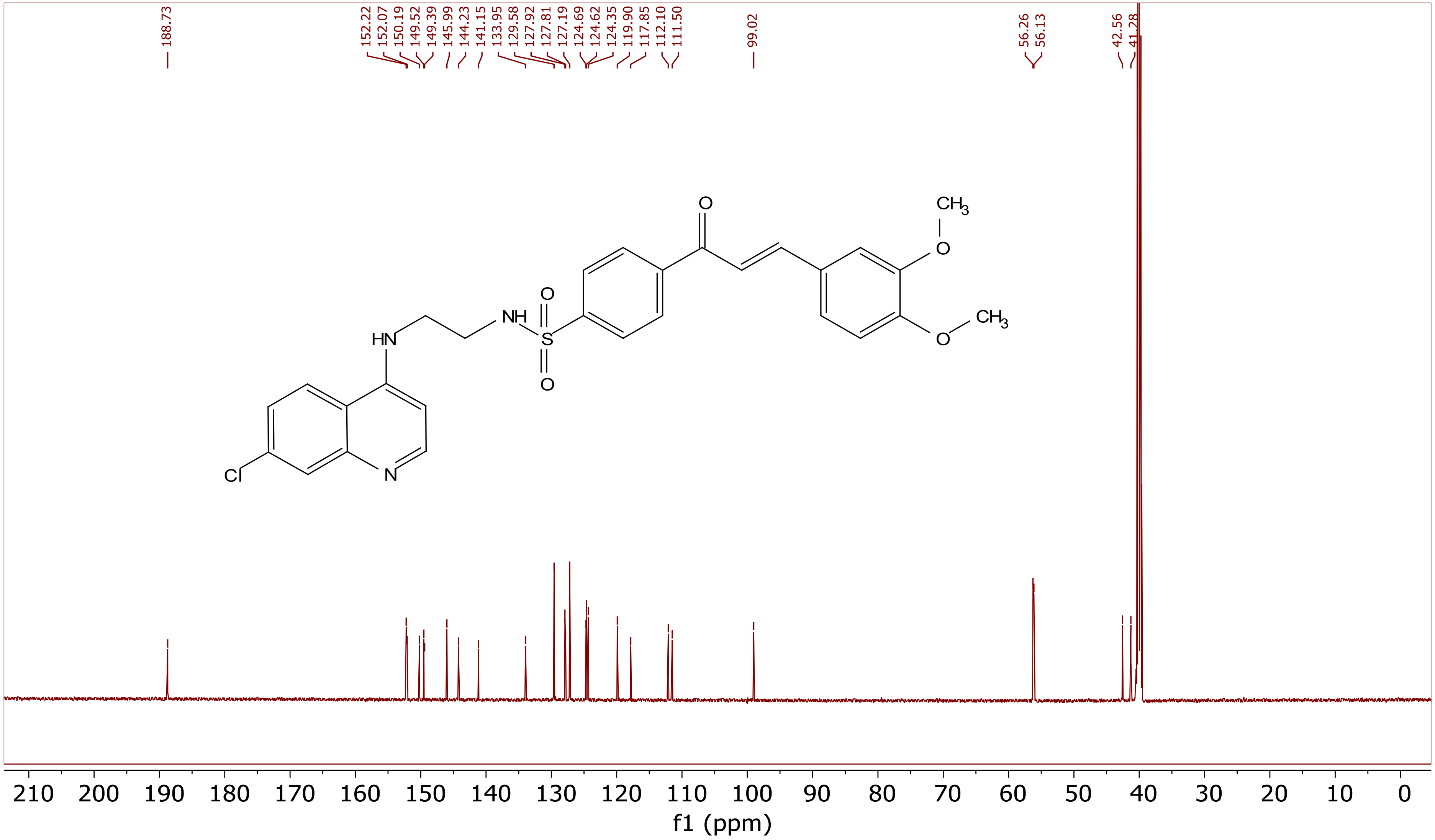

S26: ^{13}C NMR spectrum of (E)-N-(3-((7-Chloroquinolin-4-yl)amino)propyl)-4-(3-(3,4-dimethoxyphenyl)acryloyl)benzenesulfonamide **14**

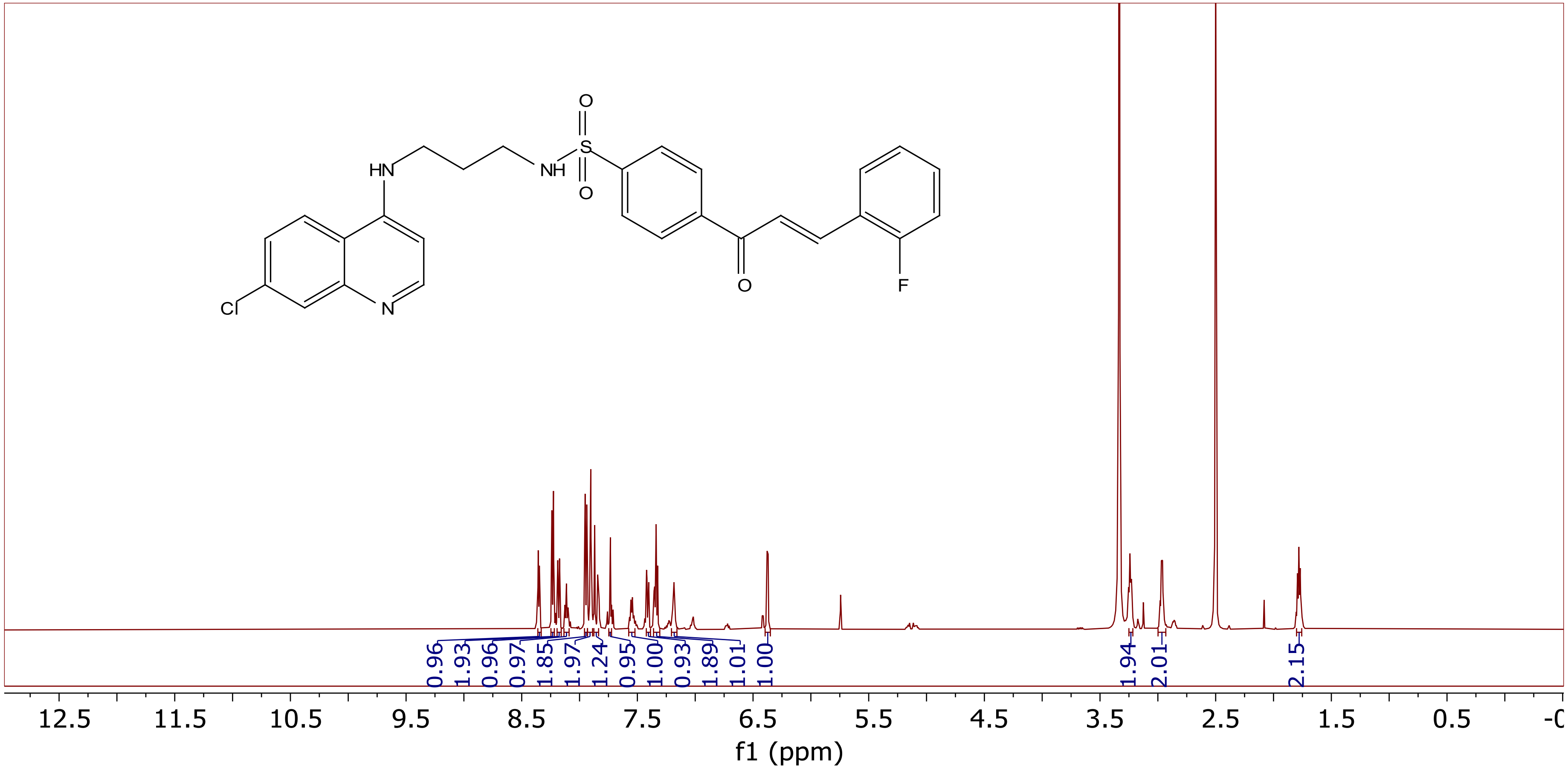

S27: ^{1}H NMR of (E)-N-(3-((7-Chloroquinolin-4-yl)amino)propyl)-4-(3-(2-fluorophenyl)acryloyl)benzenesulfonamide **15**

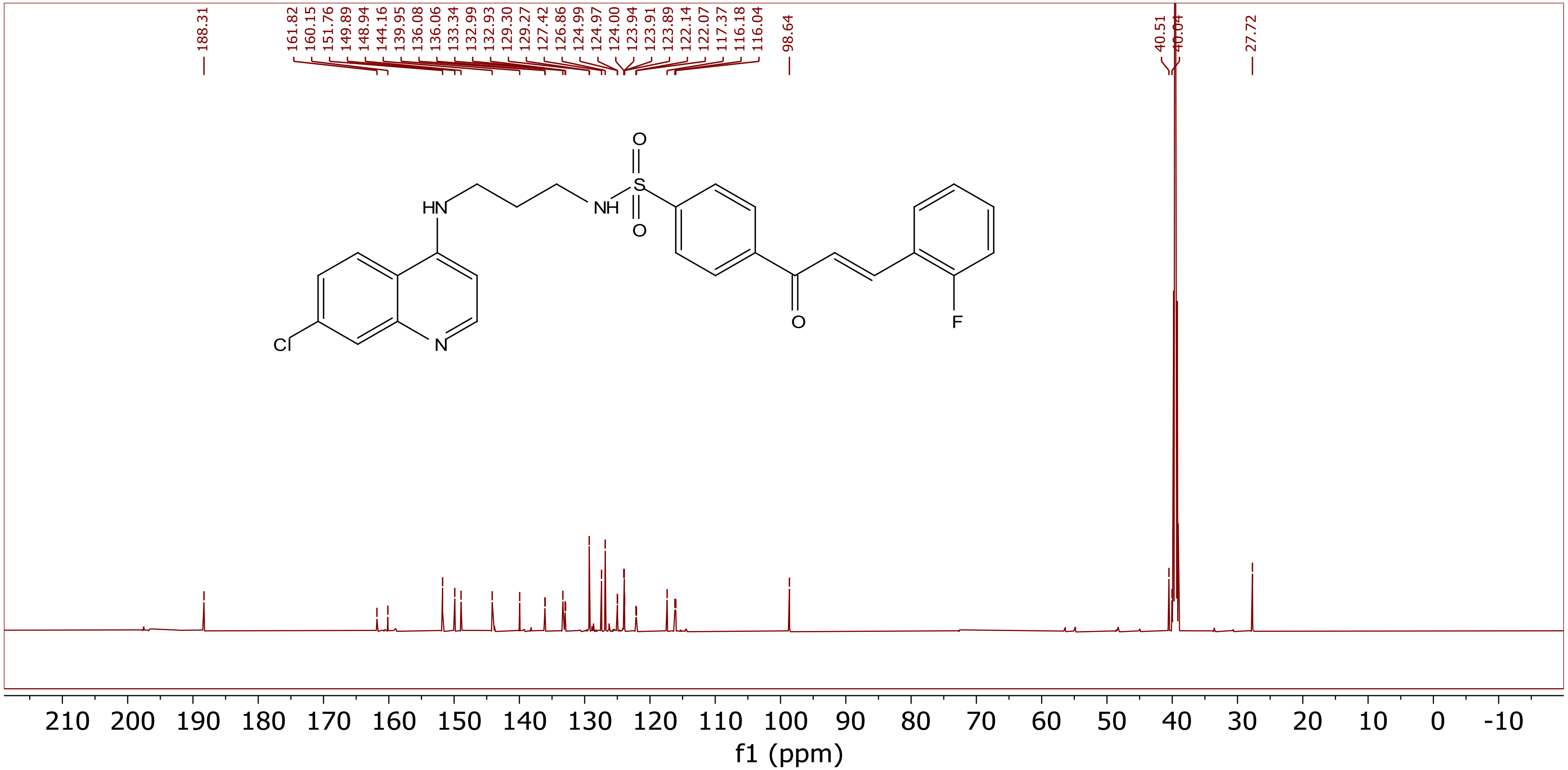

S28: ^{13}C NMR of (E)-N-(3-((7-Chloroquinolin-4-yl)amino)propyl)-4-(3-(2-fluorophenyl)acryloyl)benzenesulfonamide **15**

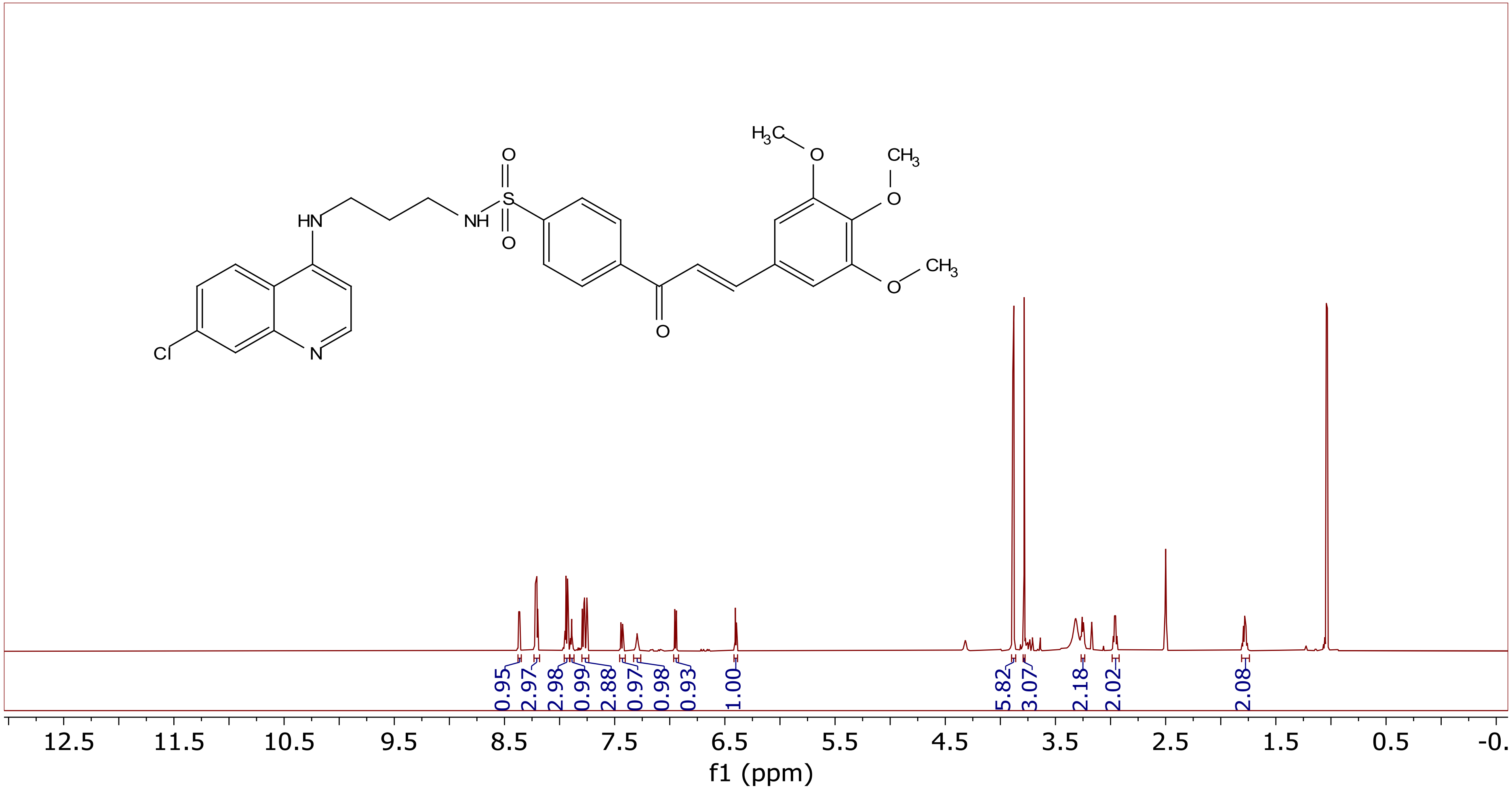

S29: ^{1}H NMR spectrum of (E)-N-(3-((7-Chloroquinolin-4-yl)amino)propyl)-4-(3-(3,4,5-trimethoxyphenyl)acryloyl)benzenesulfonamide **16**

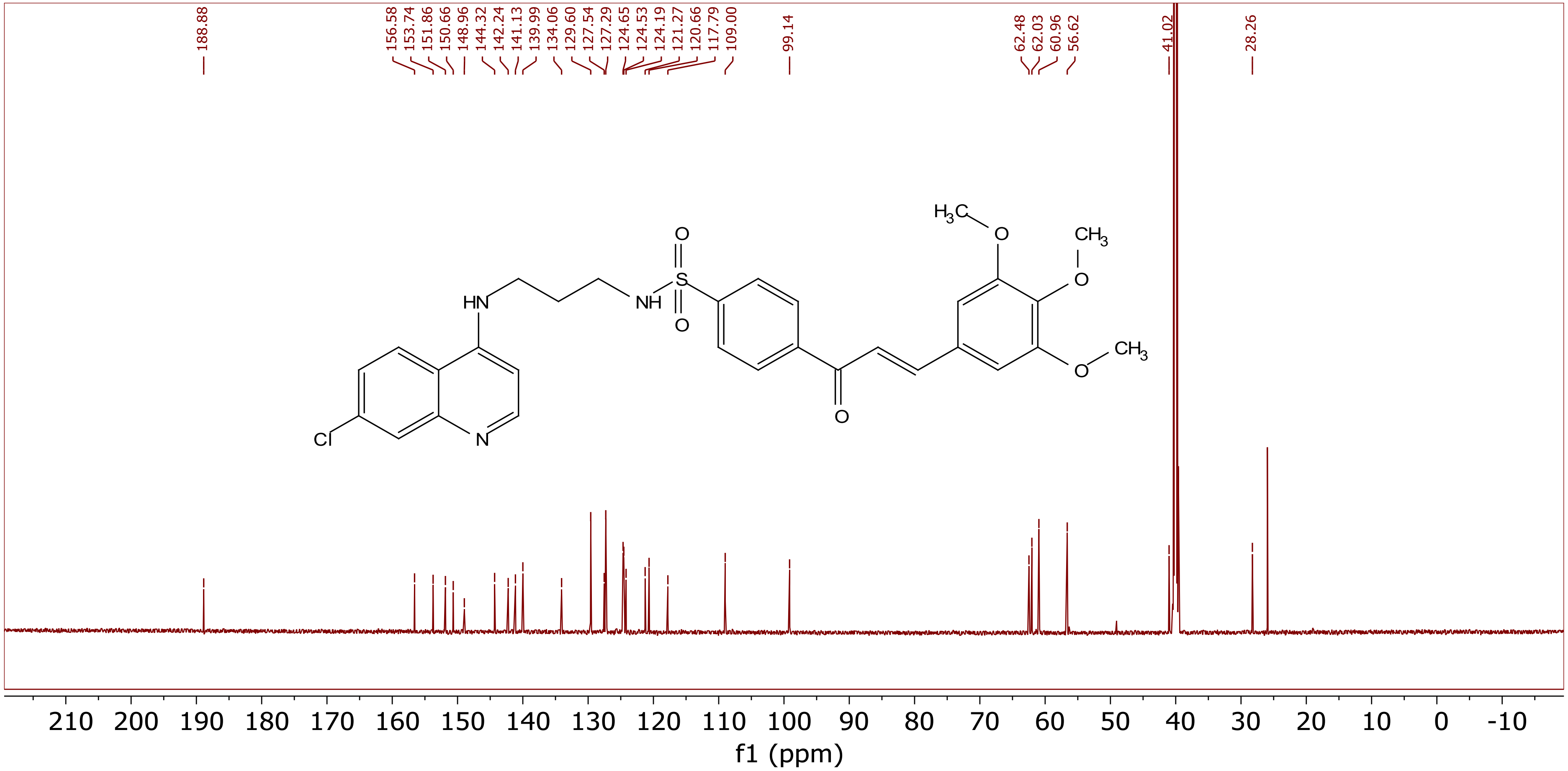

S30: ^{13}C NMR spectrum of (E)-N-(3-((7-Chloroquinolin-4-yl)amino)propyl)-4-(3-(3,4,5-trimethoxyphenyl)acryloyl)benzenesulfonamide **16**

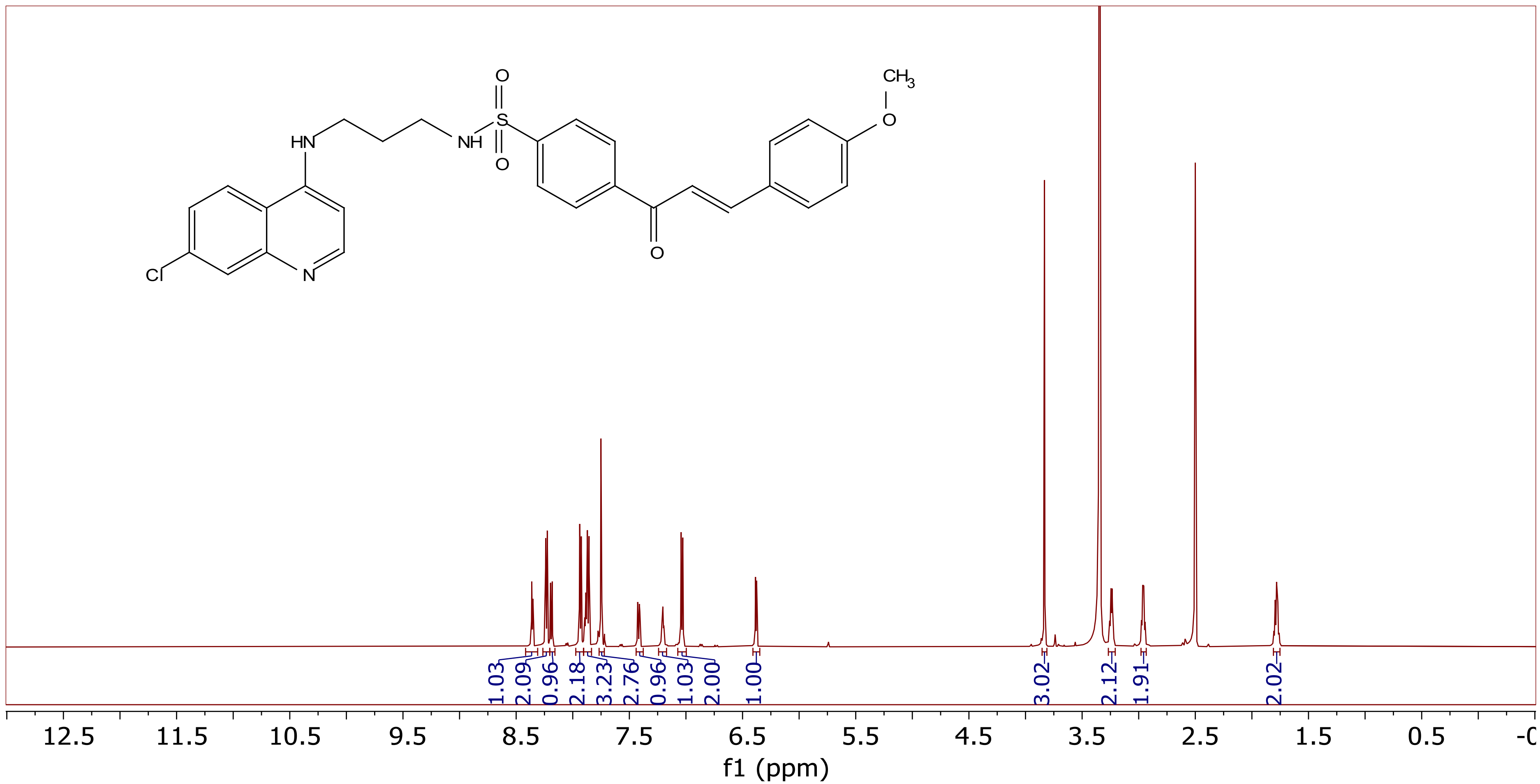

S31: ^{1}H NMR spectrum of (*E*)-*N*-(2-((7-Chloroquinolin-4-yl)amino)ethyl)-4-(3-(4-methoxyphenyl)acryloyl)benzenesulfonamide **17**

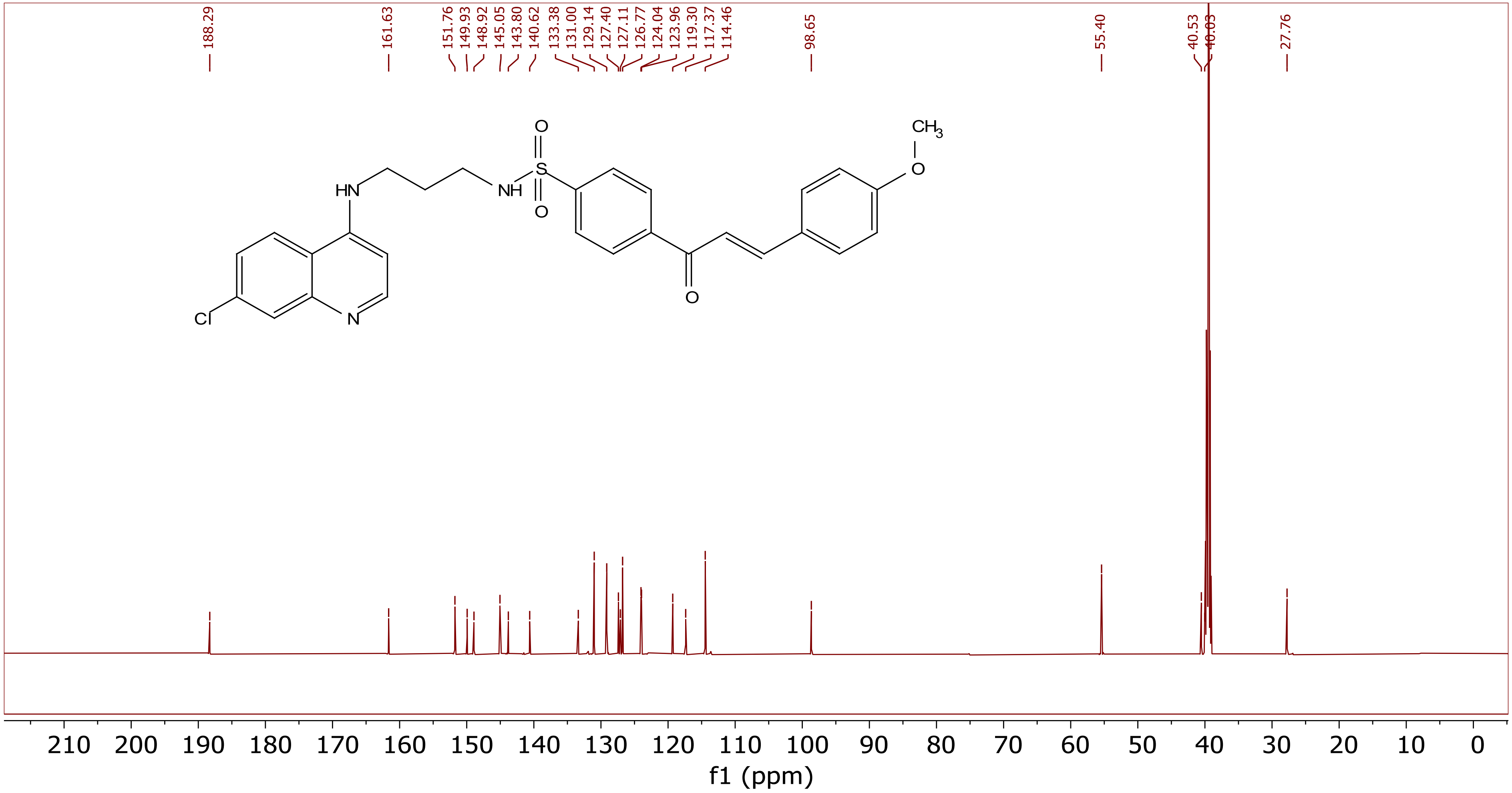

S32: ^{13}C NMR spectrum of (E)-N-(2-((7-Chloroquinolin-4-yl)amino)ethyl)-4-(3-(4-methoxyphenyl)acryloyl)benzenesulfonamide **17**

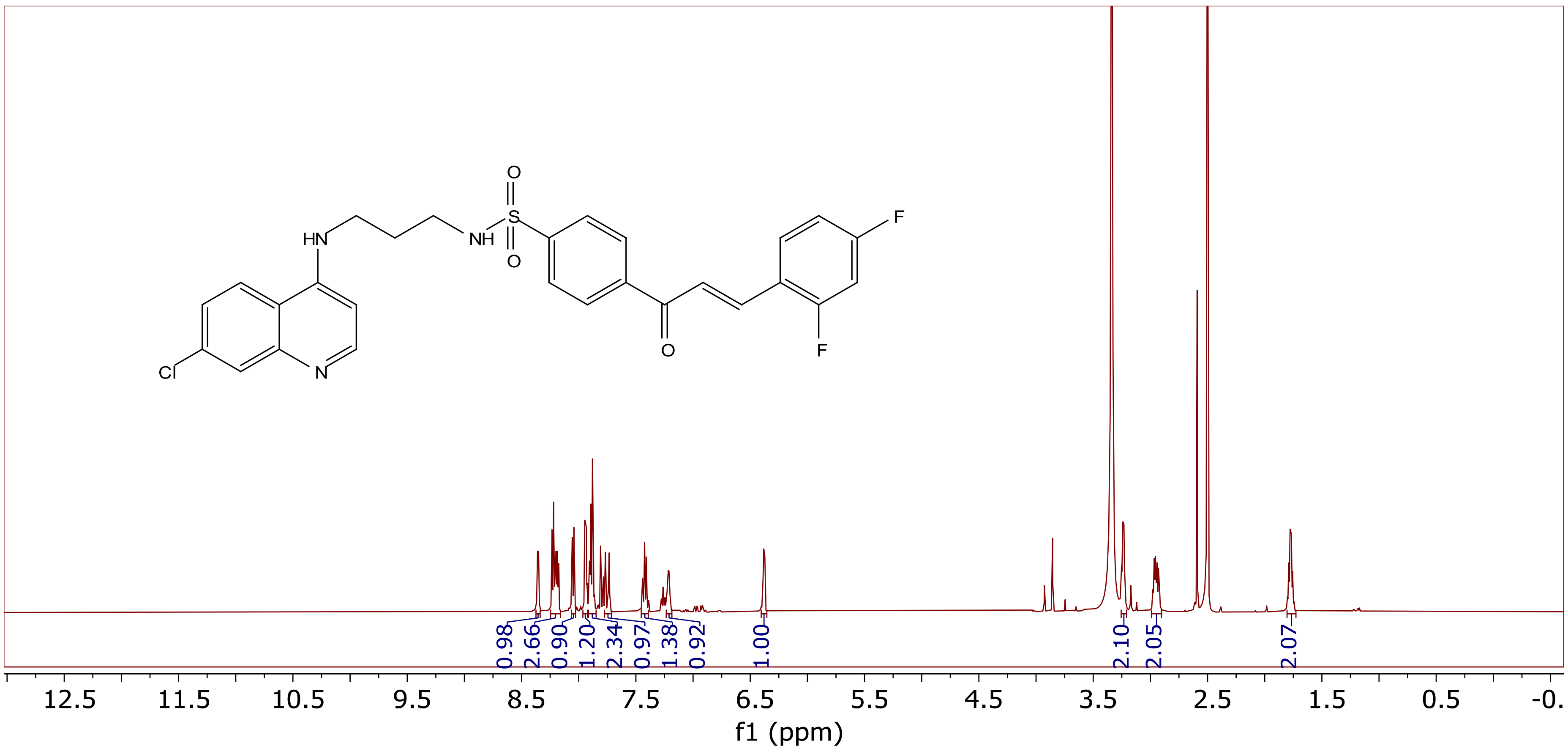

S33: ^{1}H NMR spectrum of (*E*)-*N*-(3-((7-Chloroquinolin-4-yl)amino)propyl)-4-(3-(2,4-difluorophenyl)acryloyl)benzenesulfonamide **18**

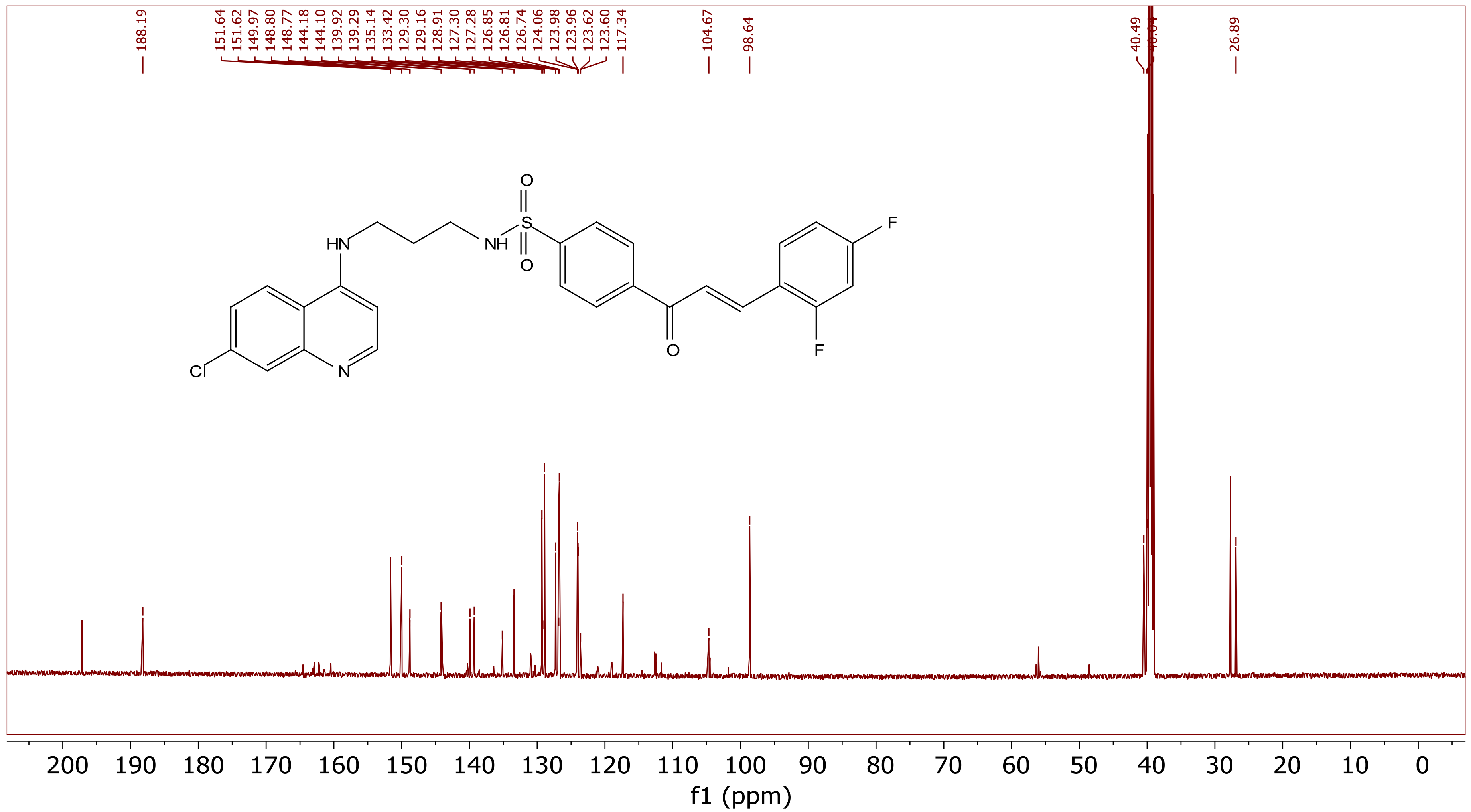

S34: ^{13}C NMR spectrum of (*E*)-*N*-(3-((7-Chloroquinolin-4-yl)amino)propyl)-4-(3-(2,4-difluorophenyl)acryloyl)benzenesulfonamide **18**

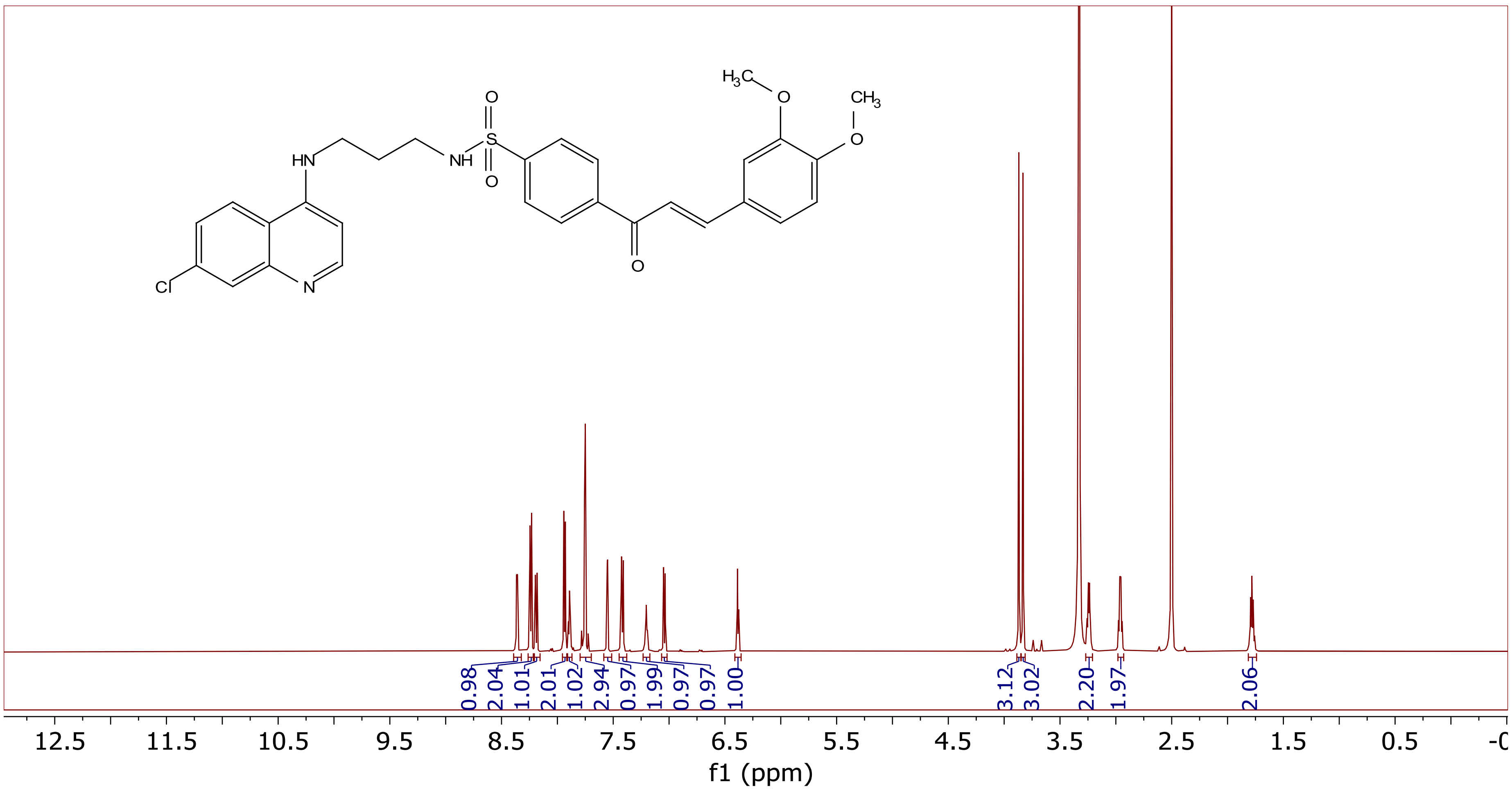

S35: ^{1}H NMR spectrum of (*E*)-*N*-(2-((7-Chloroquinolin-4-yl)amino)ethyl)-4-(3-(3,4-dimethoxyphenyl)acryloyl)benzenesulfonamide **19**

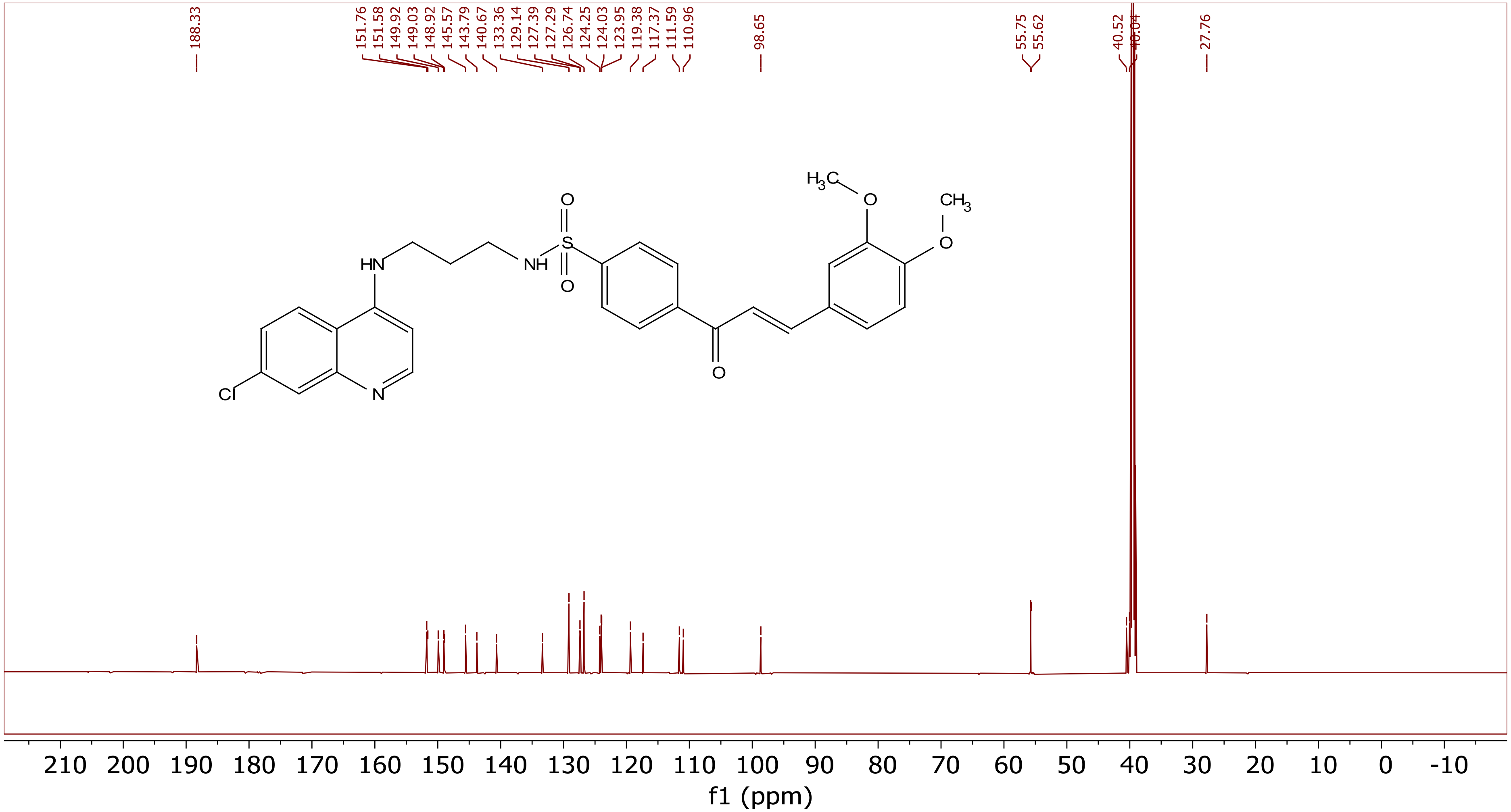

S36: ^{13}C NMR spectrum of (*E*)-*N*-(2-((7-Chloroquinolin-4-yl)amino)ethyl)-4-(3-(3,4-dimethoxyphenyl)acryloyl)benzenesulfonamide **19**

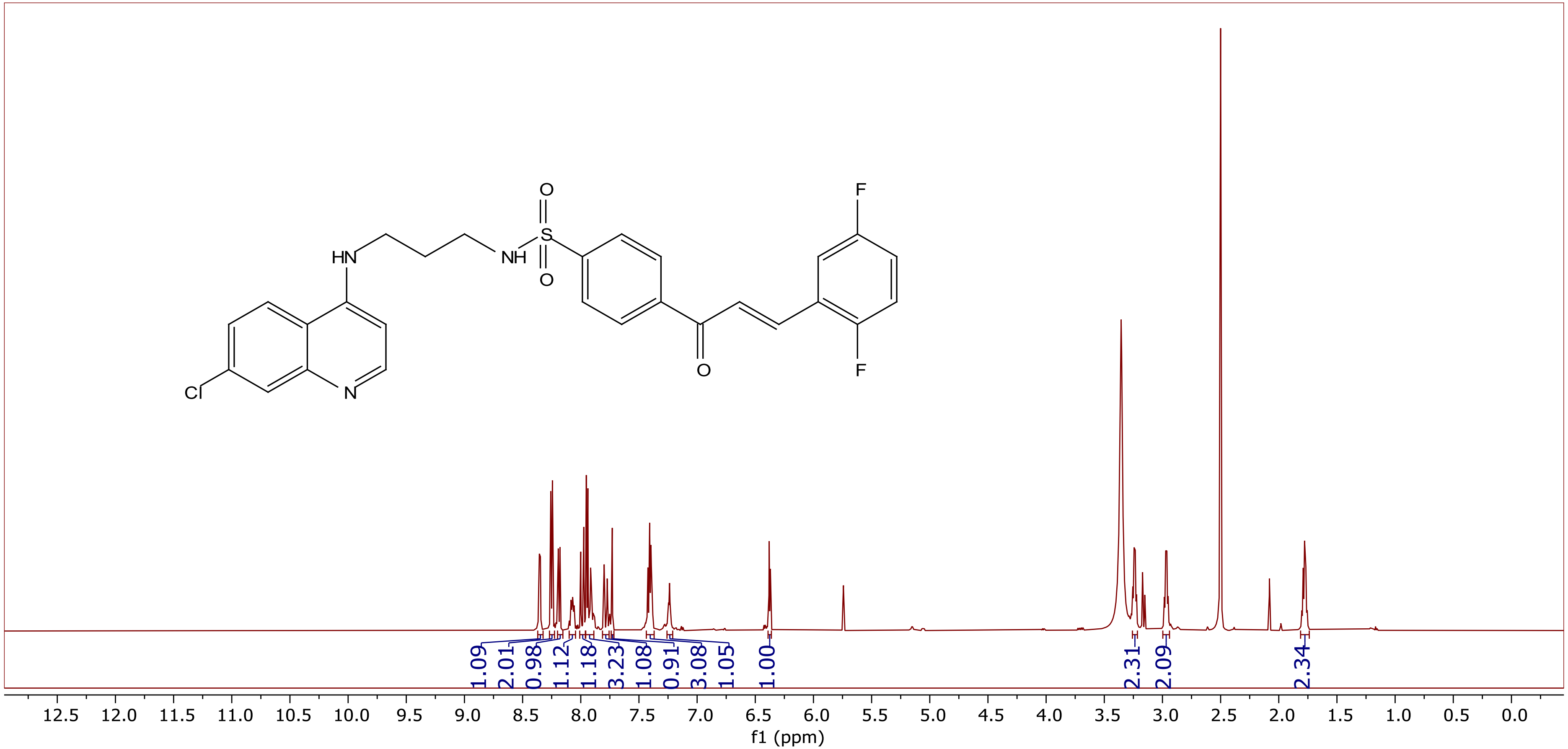

S37: ^{1}H NMR spectrum of (E)-N-(3-((7-Chloroquinolin-4-yl)amino)propyl)-4-(3-(2,5-difluorophenyl)acryloyl)benzenesulfonamide **20**

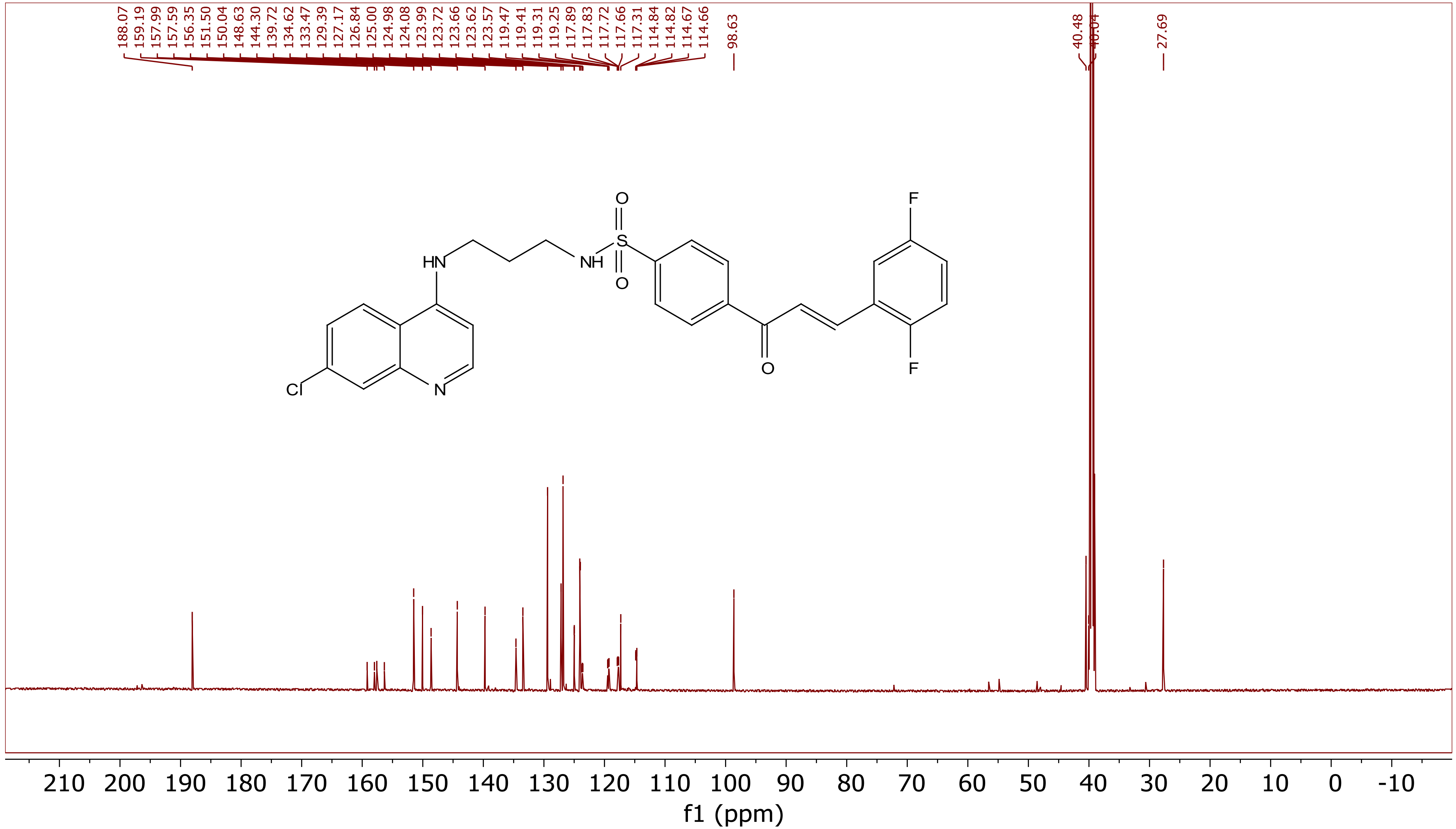

S38: ^{13}C NMR spectrum of (E)-N-(3-((7-Chloroquinolin-4-yl)amino)propyl)-4-(3-(2,5-difluorophenyl)acryloyl)benzenesulfonamide **20**

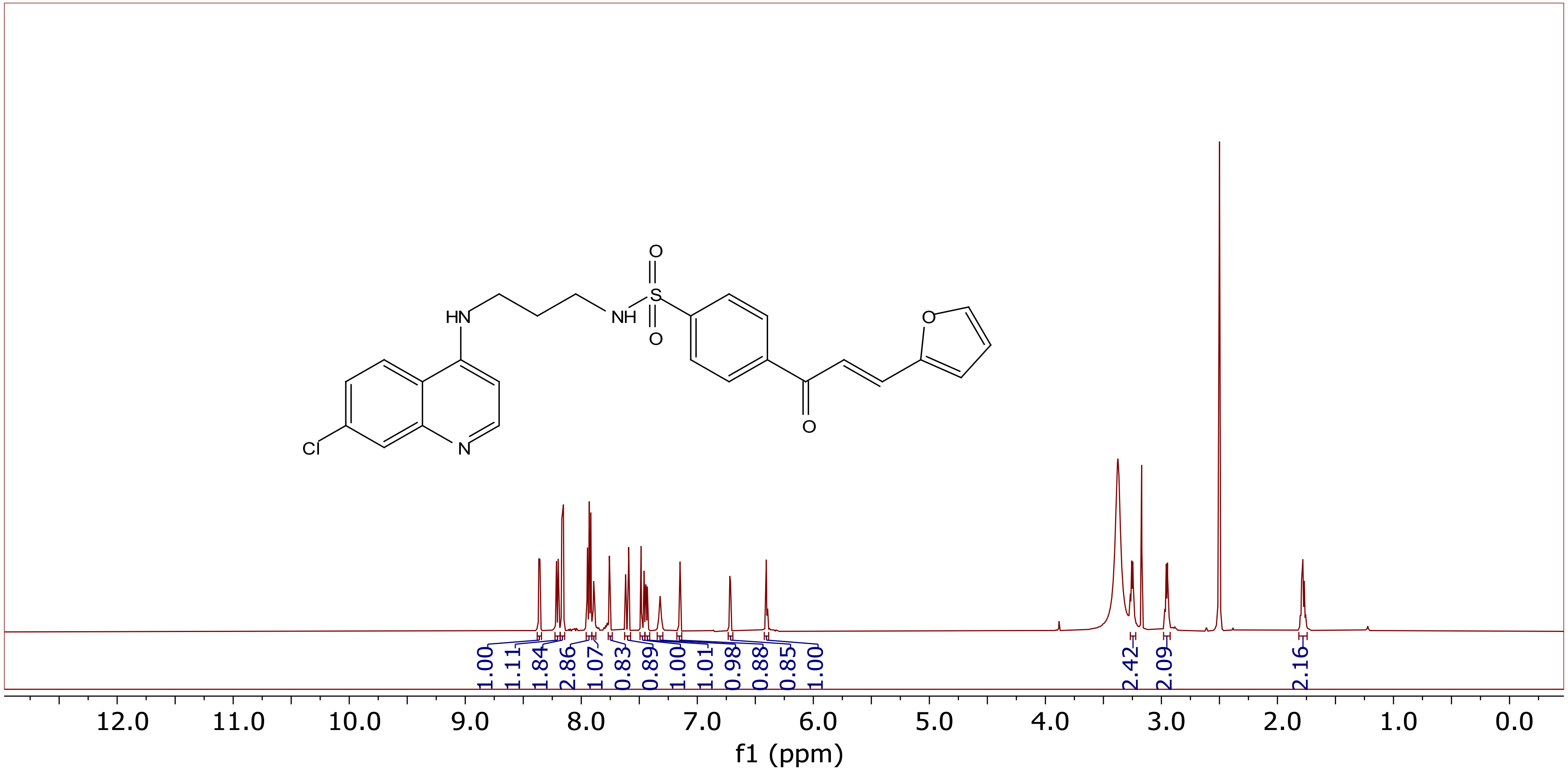

S39: ^{1}H NMR spectrum of (*E*)-*N*-(3-((7-Chloroquinolin-4-yl)amino)propyl)-4-(3-(furan-2-yl)acryloyl)benzenesulfonamide **21**

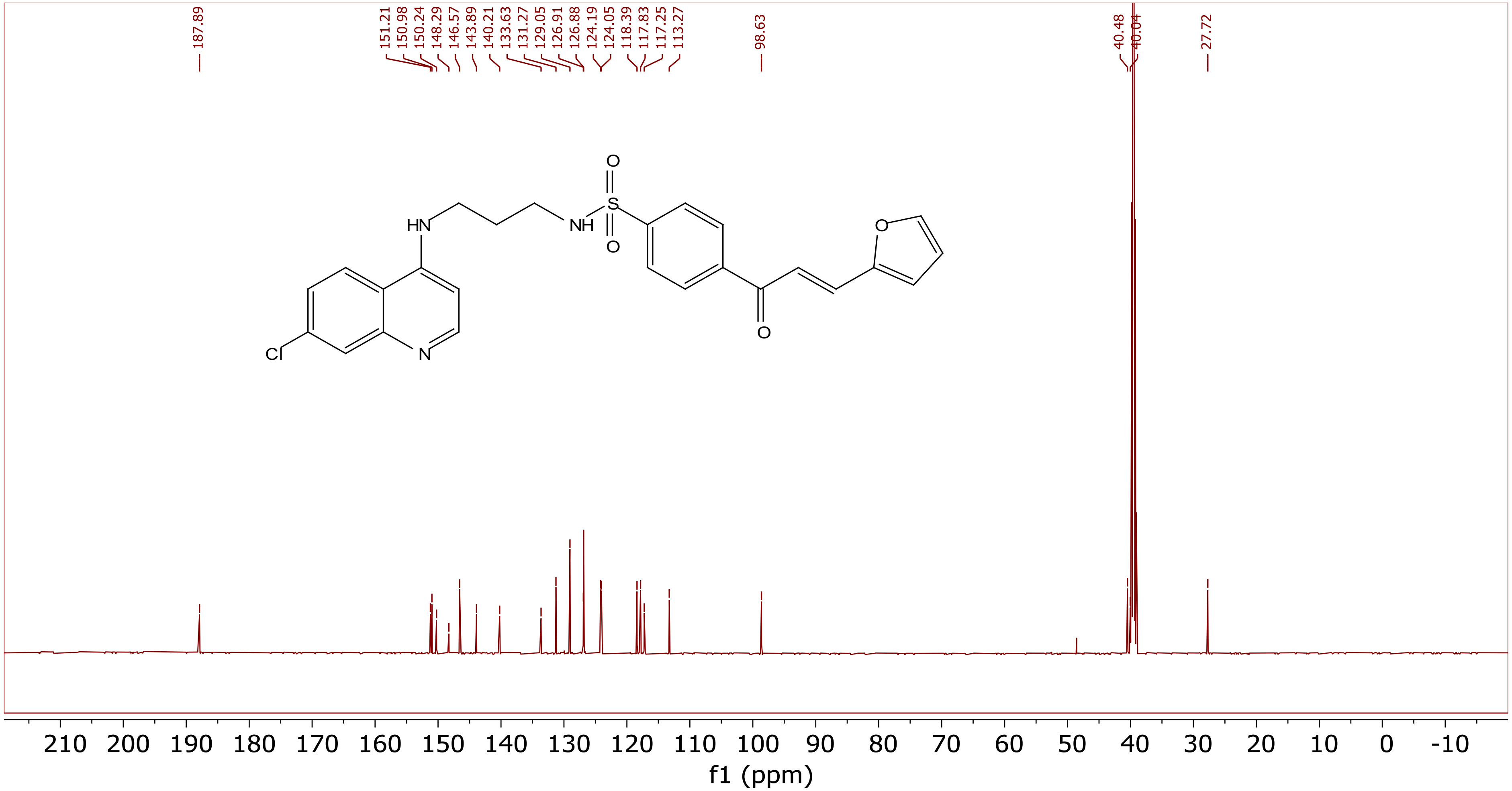

S40: ^{13}C NMR spectrum of (E)-N-(3-((7-Chloroquinolin-4-yl)amino)propyl)-4-(3-(furan-2-yl)acryloyl)benzenesulfonamide **21**

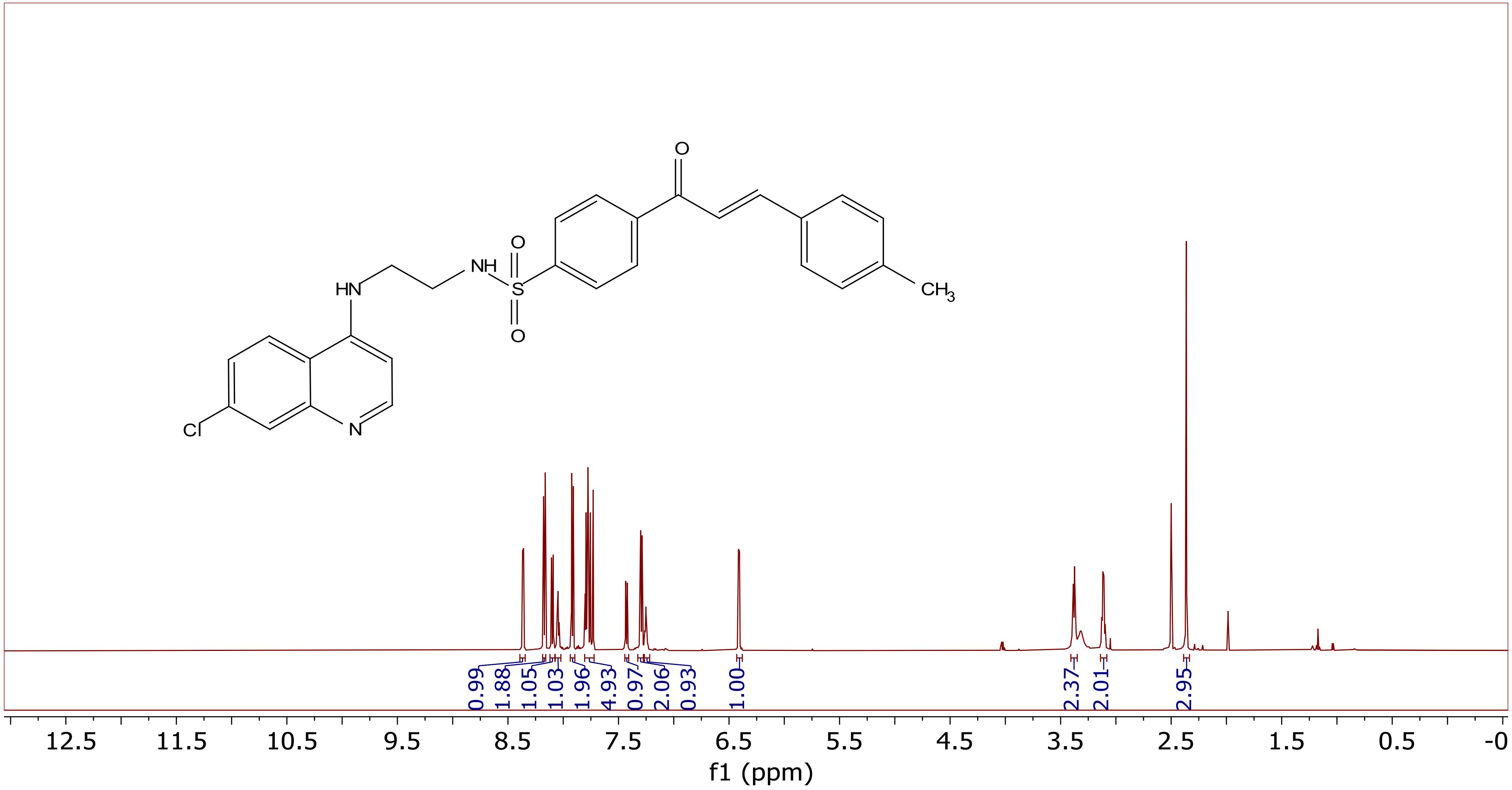

S41: ^{1}H NMR spectrum of (*E*)-*N*-(2-((7-Chloroquinolin-4-yl)amino)ethyl)-4-(3-(*p*-tolyl)acryloyl)benzenesulfonamide **22**

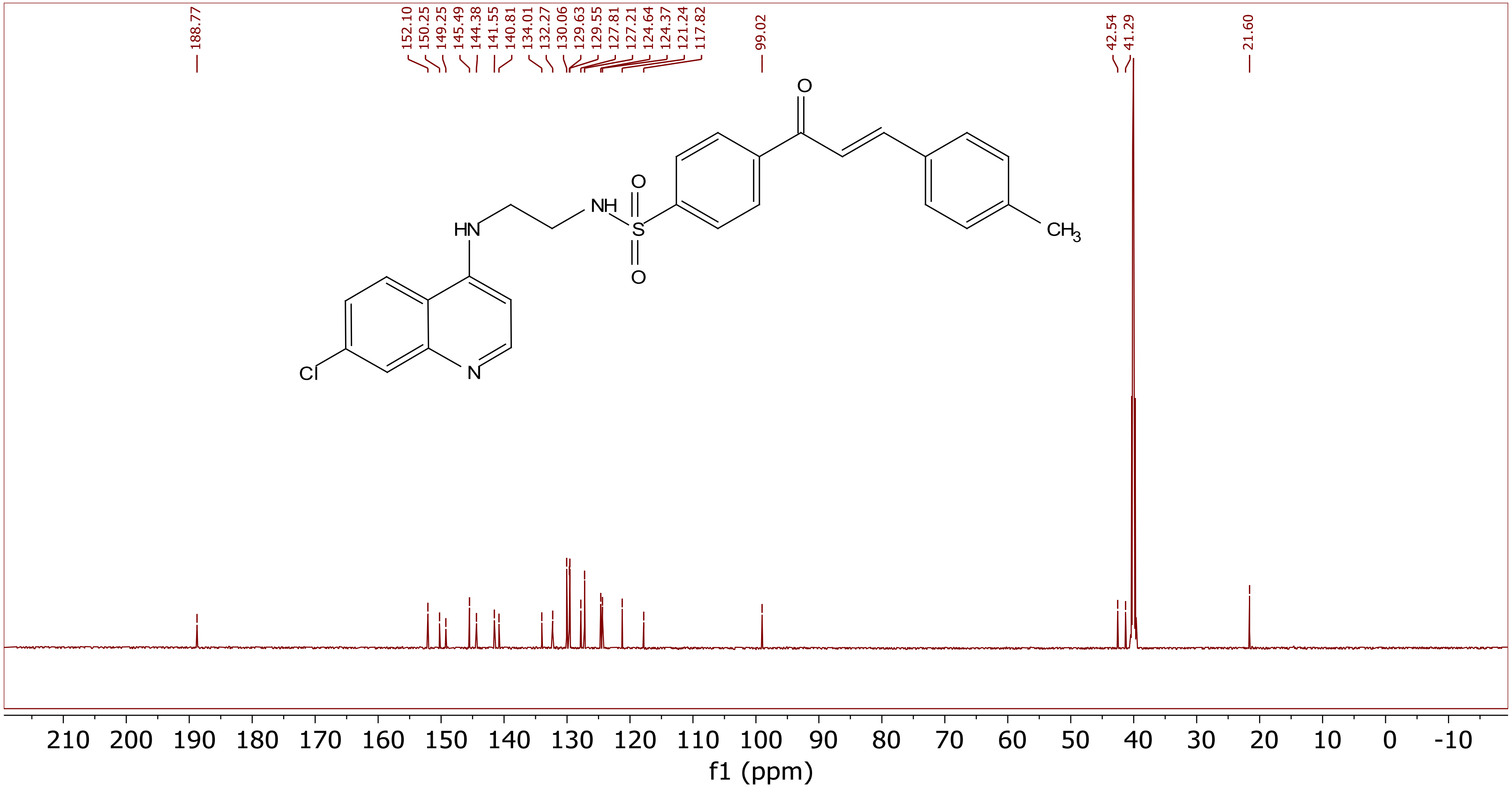

S42: ^{13}C NMR spectrum of (E)-N-(2-((7-Chloroquinolin-4-yl)amino)ethyl)-4-(3-(p-tolyl)acryloyl)benzenesulfonamide **22**

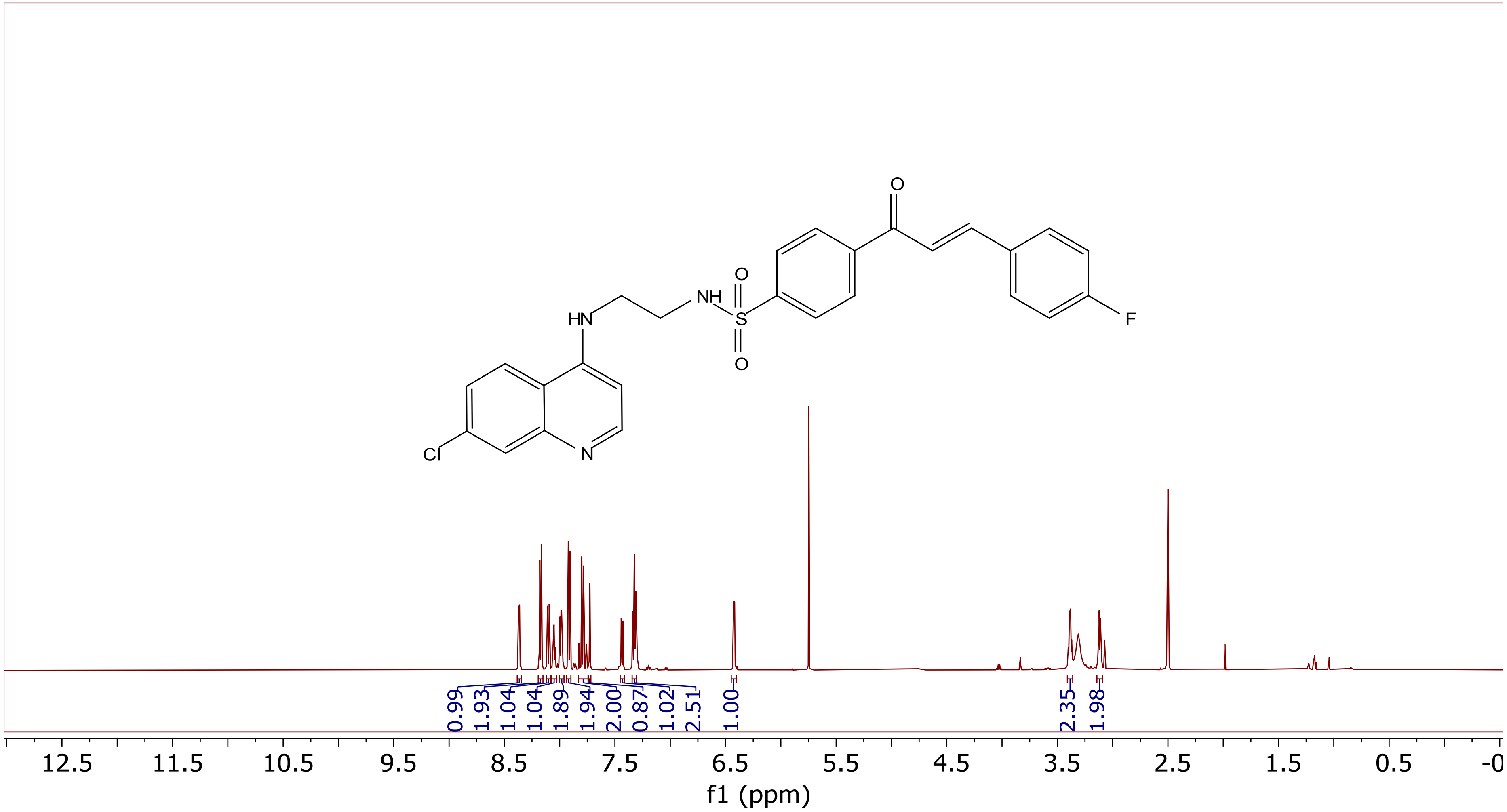

S43: ^{1}H NMR spectrum of (E)-N-(2-((7-Chloroquinolin-4-yl)amino)ethyl)-4-(3-(4-fluorophenyl)acryloyl)benzenesulfonamide **24**

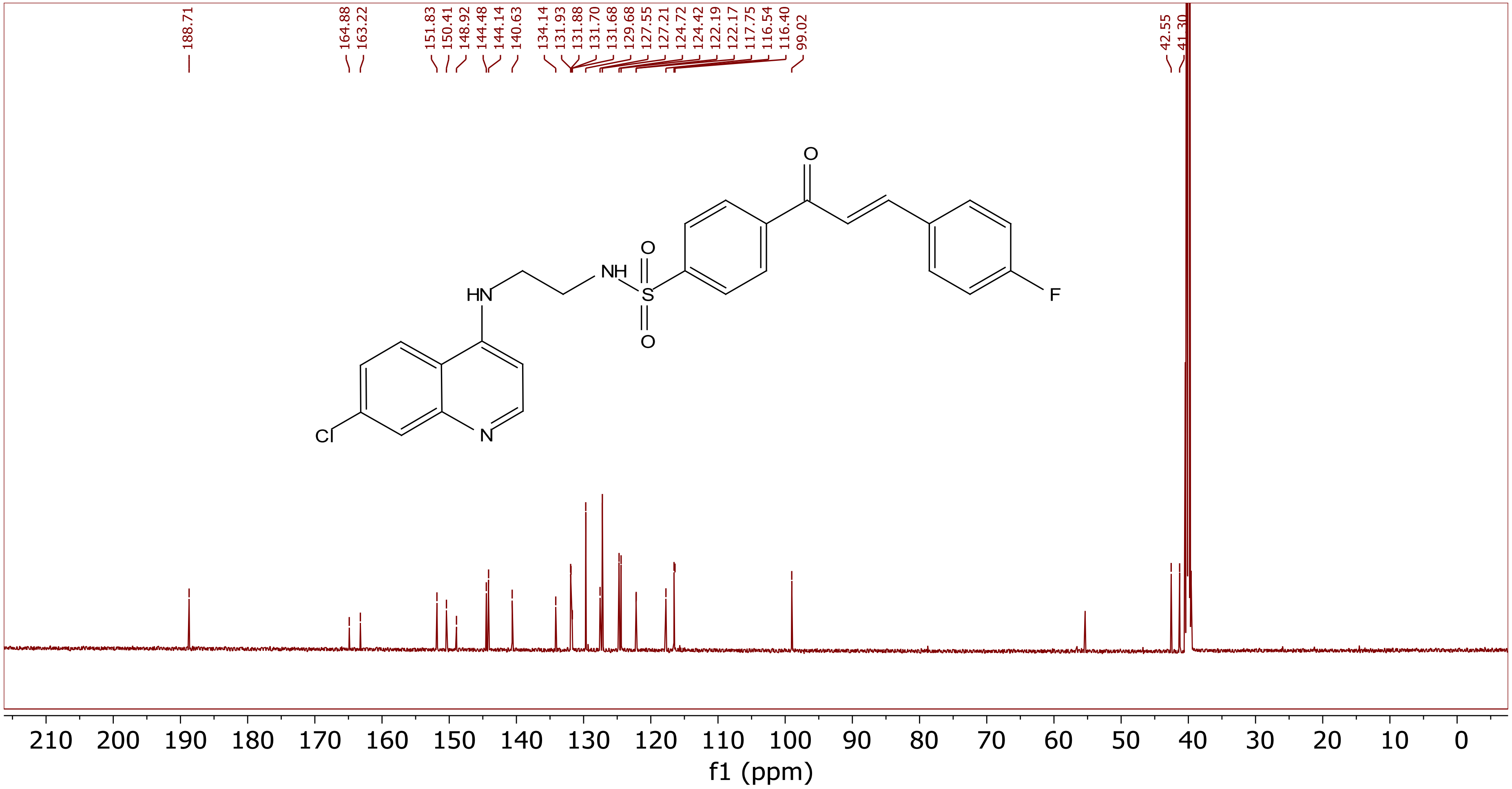

S44: ^{13}C NMR spectrum of (E)-N-(2-((7-Chloroquinolin-4-yl)amino)ethyl)-4-(3-(4-fluorophenyl)acryloyl)benzenesulfonamide **24**

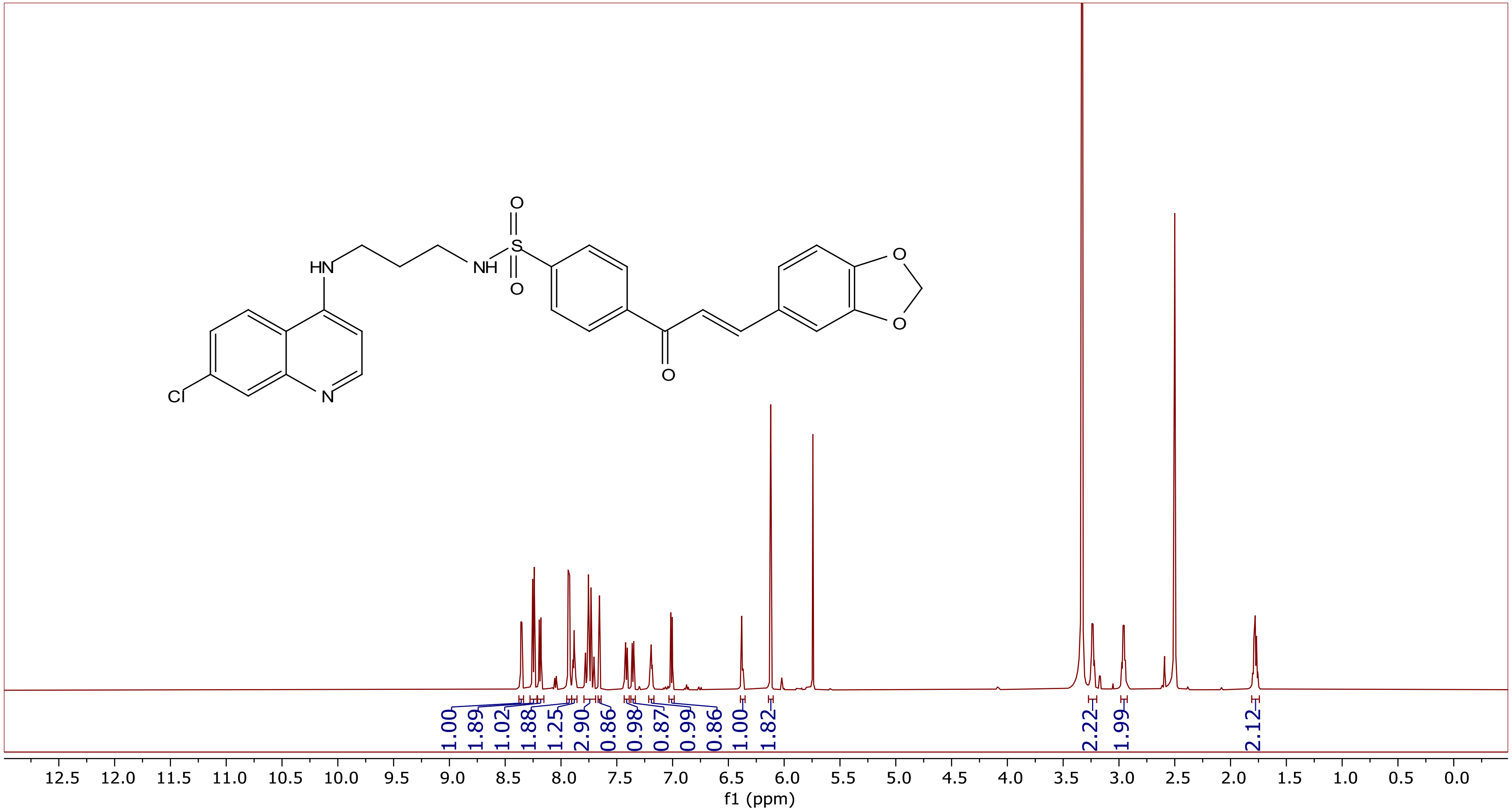

S45: ^{1}H NMR spectrum of (*E*)-4-(3-(Benzo[d][1,3]dioxol-5-yl)acryloyl)-*N*-(3-((7-chloroquinolin-4-yl)amino)propyl)benzenesulfonamide **26**

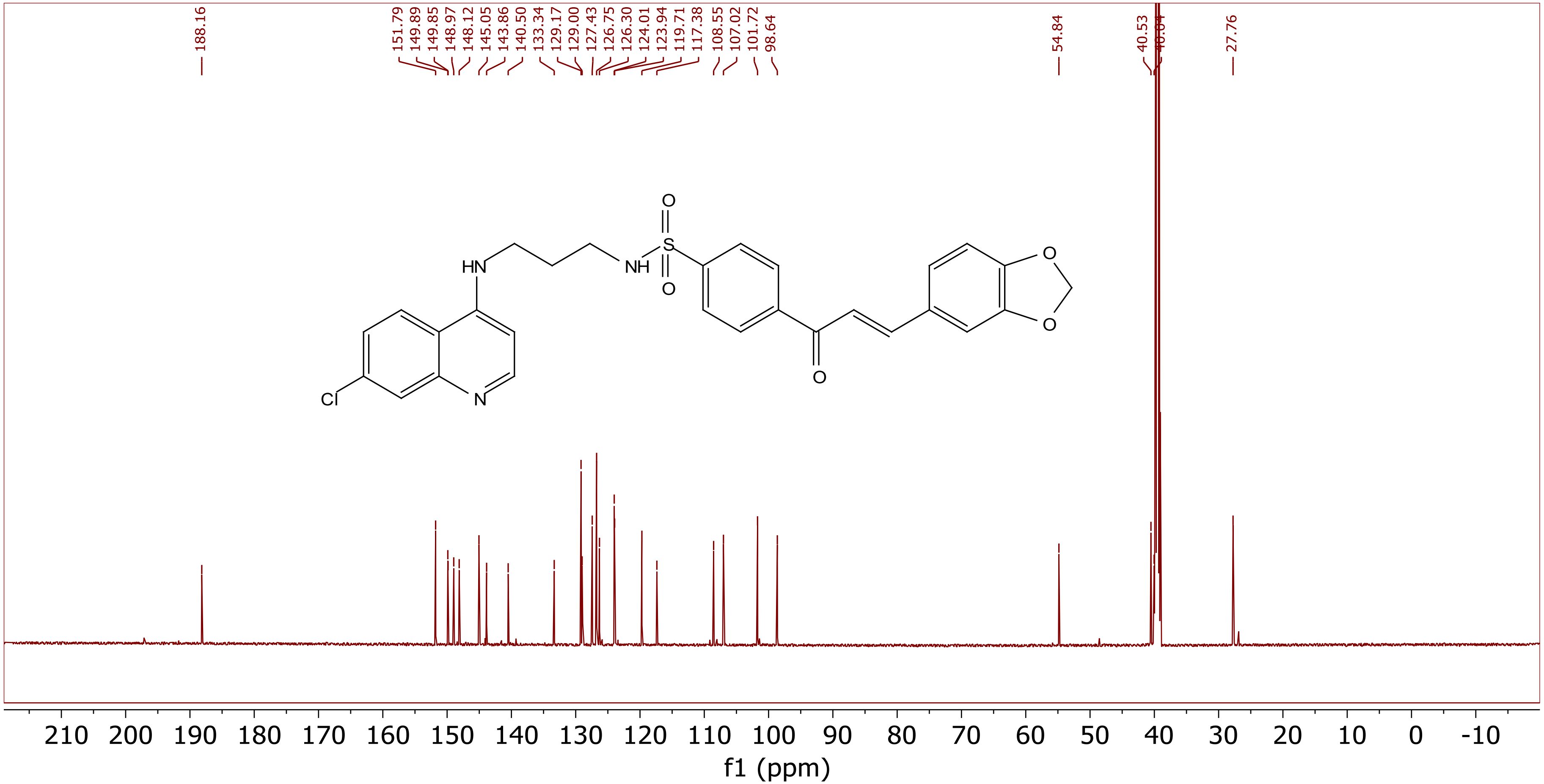

S46: ^{13}C NMR spectrum of (*E*)-4-(3-(Benzo[d][1,3]dioxol-5-yl)acryloyl)-*N*-(3-((7-chloroquinolin-4-yl)amino)propyl)benzenesulfonamide **26**

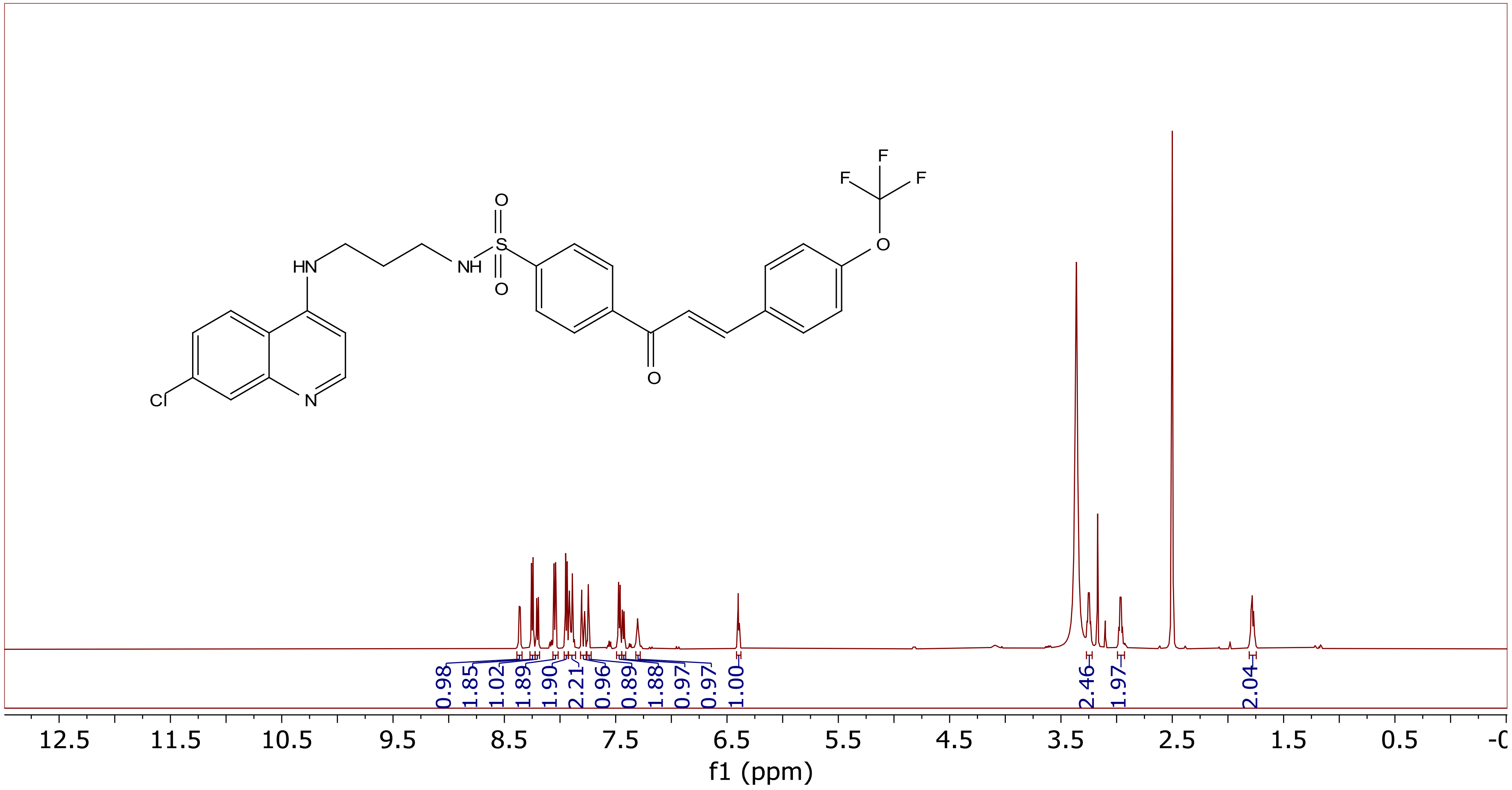

S47: ^{1}H NMR spectrum of (*E*)-*N*-(3-((7-Chloroquinolin-4-yl)amino)propyl)-4-(3-(4-(trifluoromethoxy)phenyl)acryloyl)benzenesulfonamide **27**

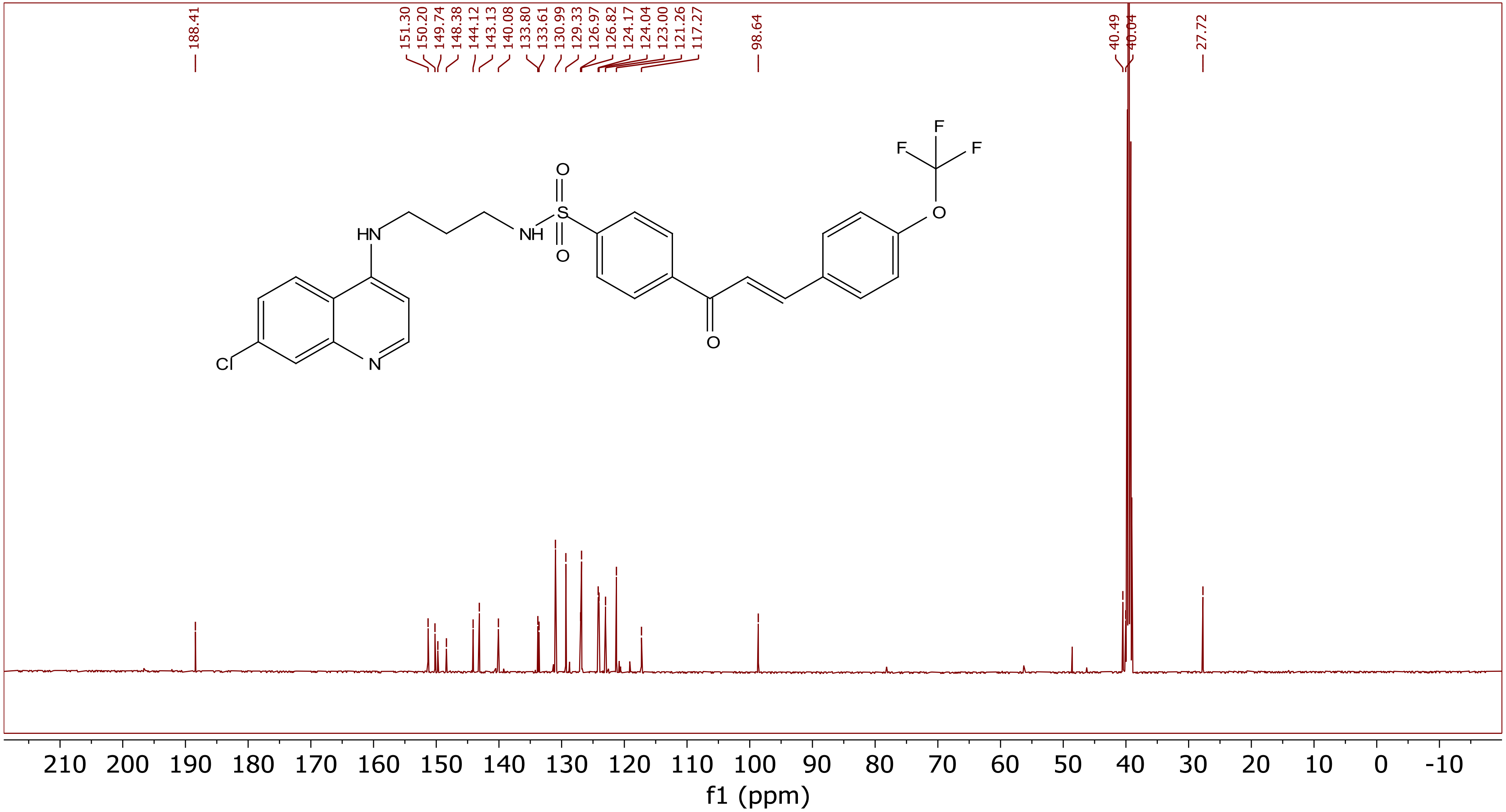

S48: ^{13}C NMR spectrum of (E)-N-(3-((7-Chloroquinolin-4-yl)amino)propyl)-4-(3-(4-(trifluoromethoxy)phenyl)acryloyl)benzenesulfonamide **27**

HPLC of representative compounds

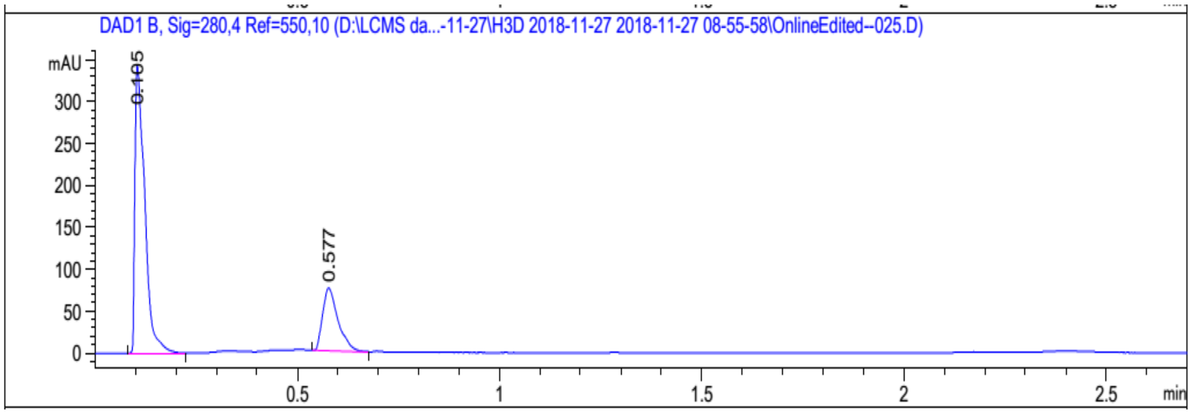

S49: HPLC chromatogram of **2**

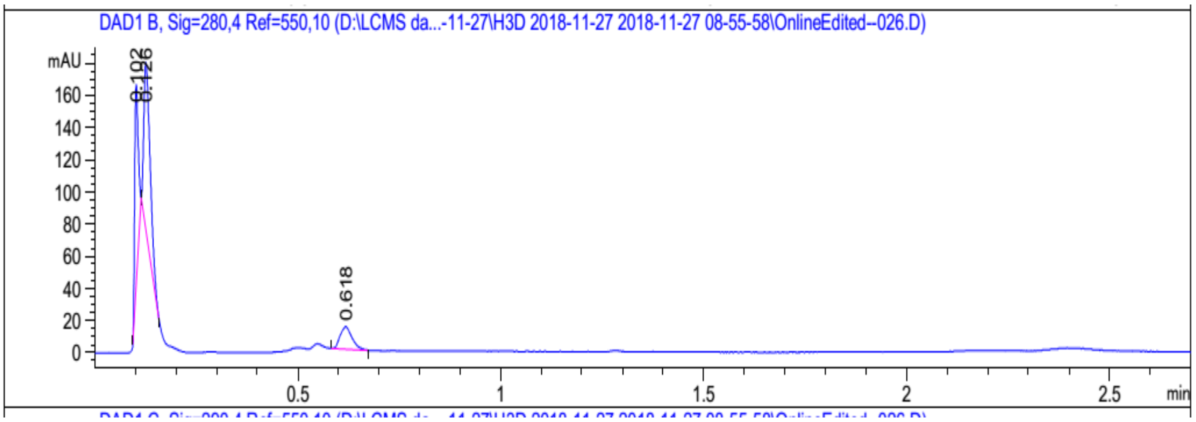

S50: HPLC chromatogram of **3**

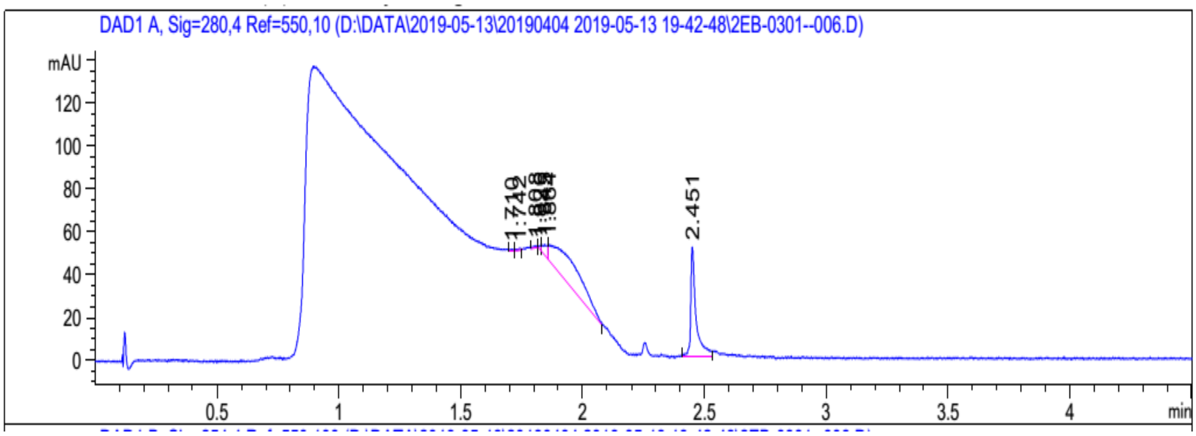

S51: HPLC chromatogram of **4**

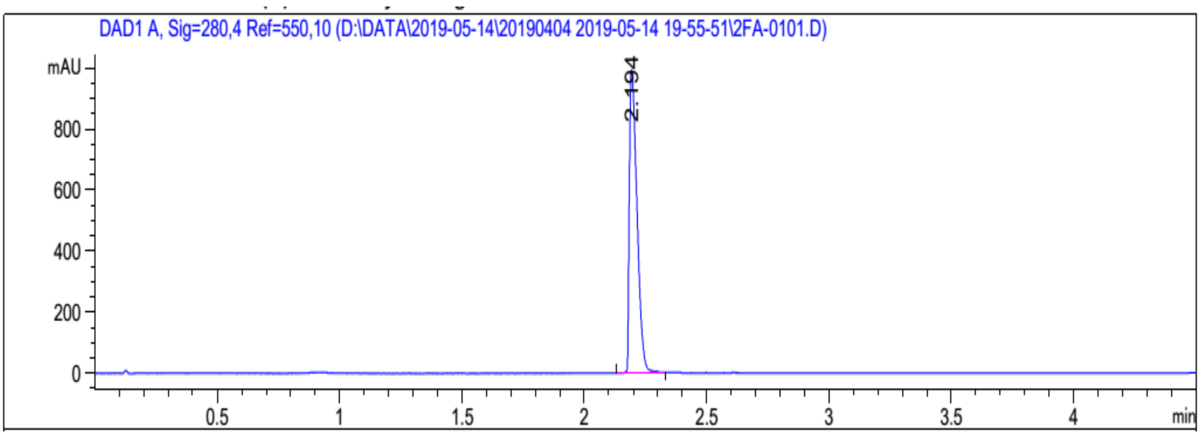

S52: HPLC chromatogram of **5**

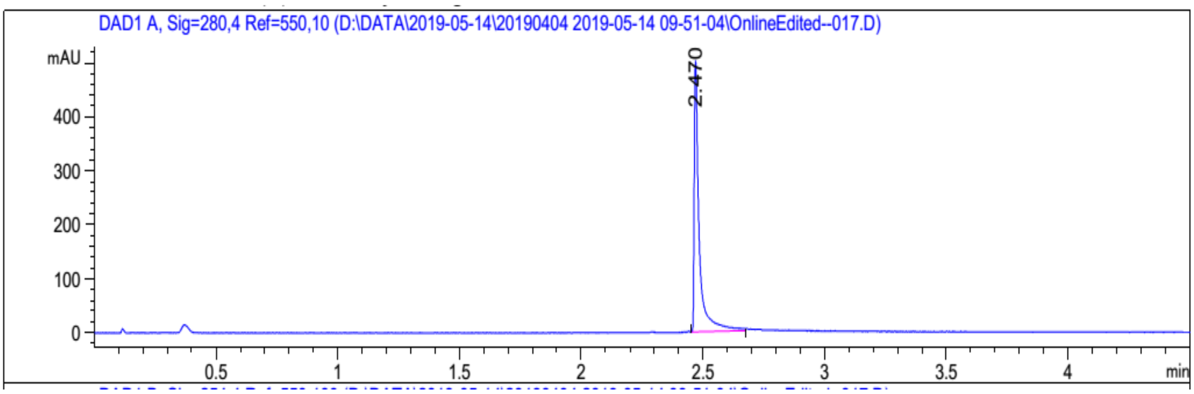

S53: HPLC chromatogram of **6**

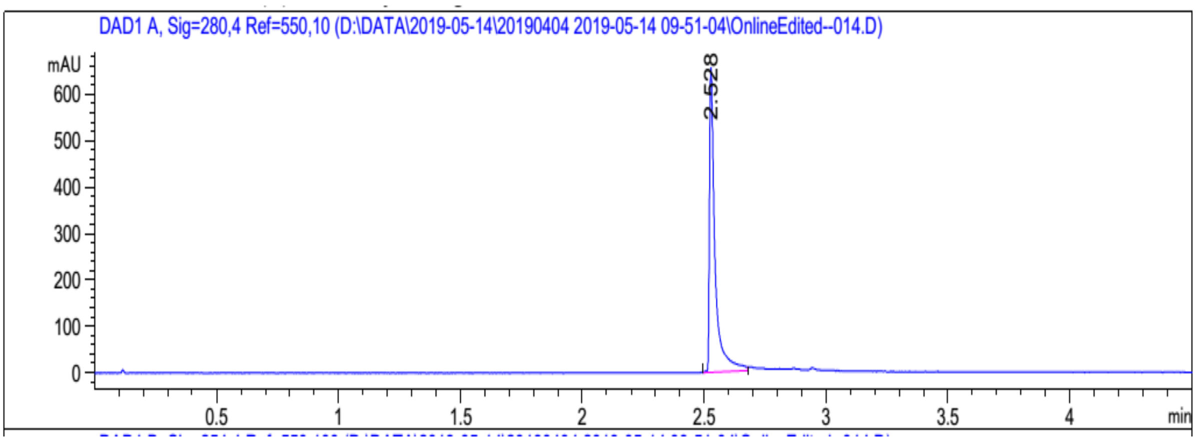

S54: HPLC chromatogram of **7**

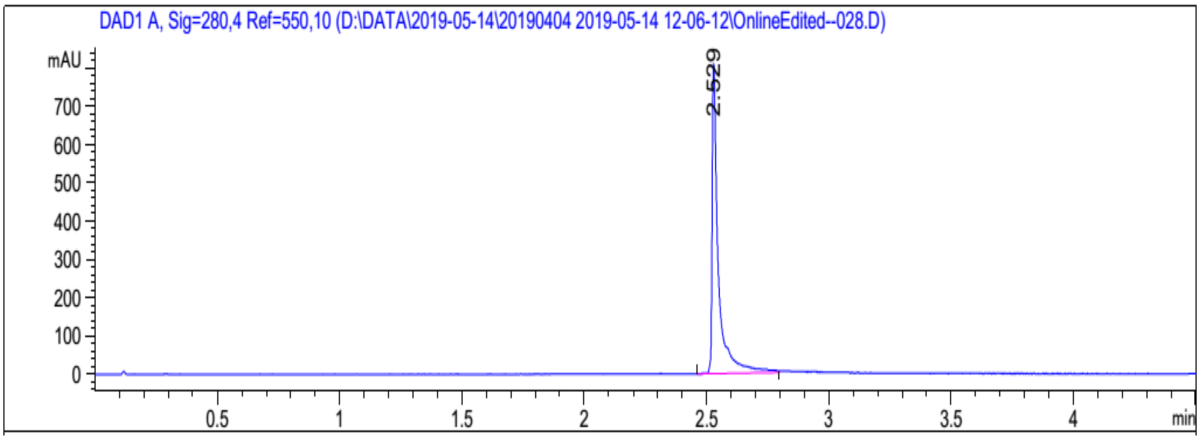

S55: HPLC chromatogram of **8**

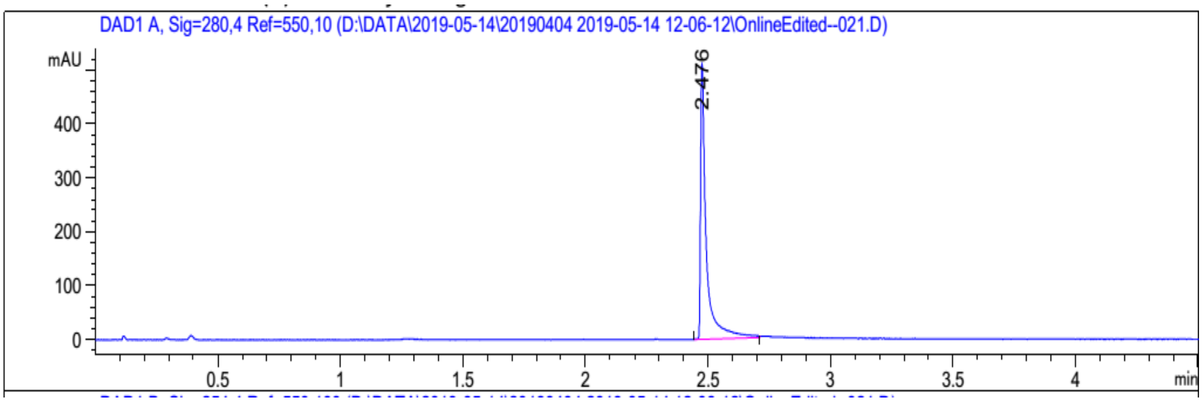

S56: HPLC chromatogram of **9**

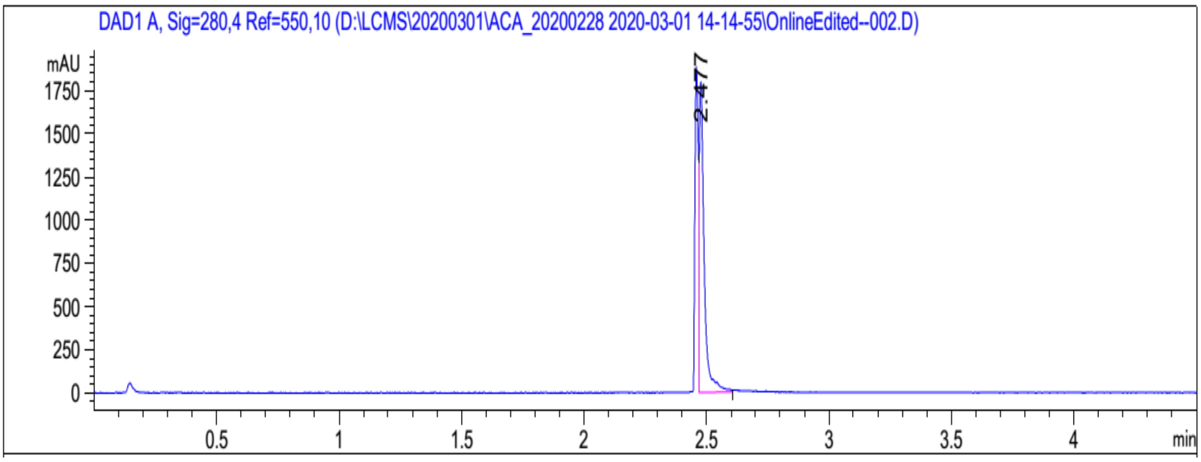

S57: HPLC chromatogram of **10**

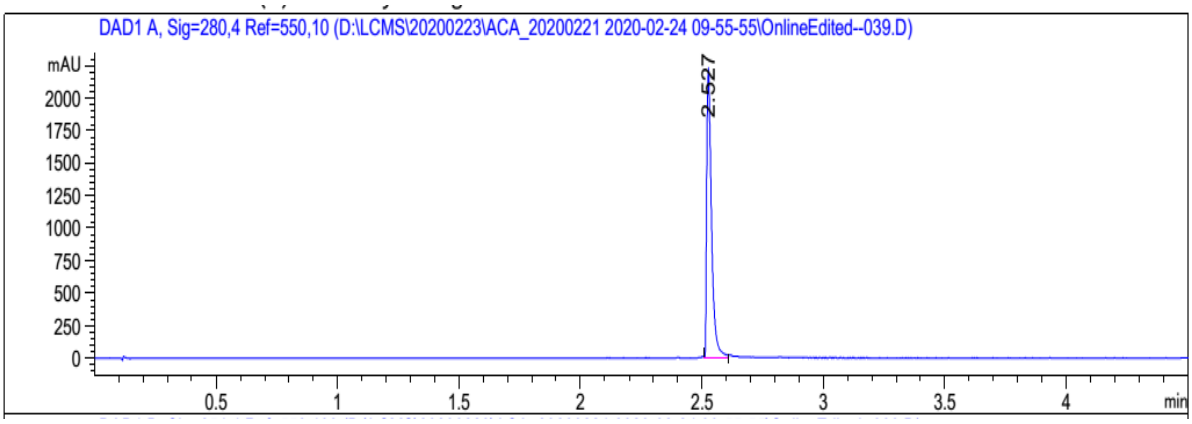

S58: HPLC chromatogram of **11**

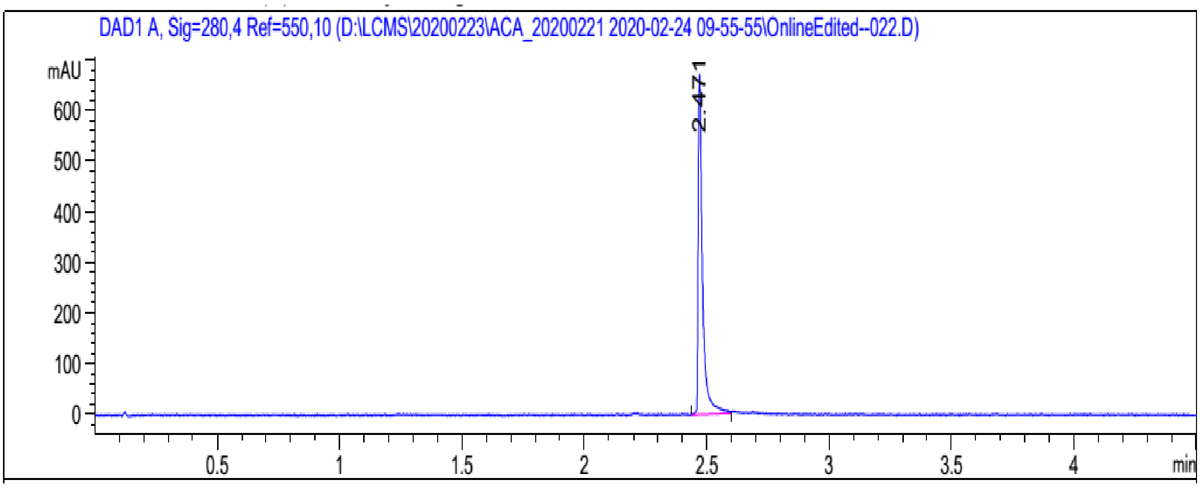

S59: HPLC chromatogram of **12**

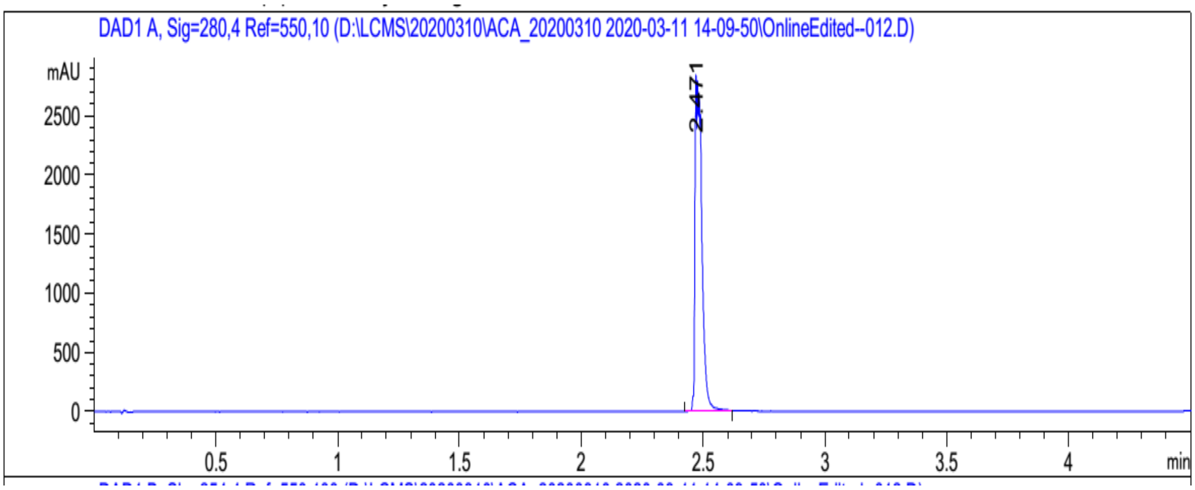

S60: HPLC chromatogram of **13**

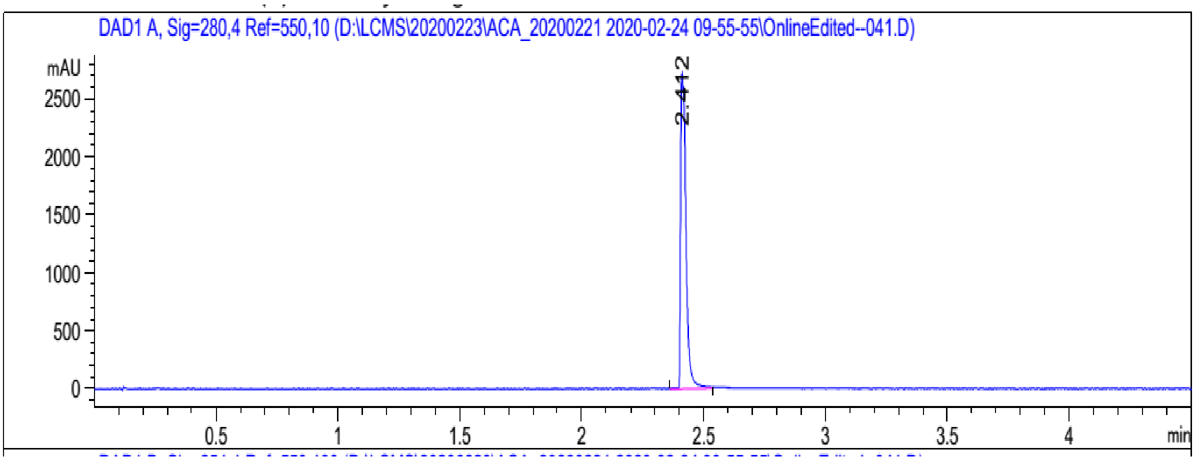

S61: HPLC chromatogram of **14**

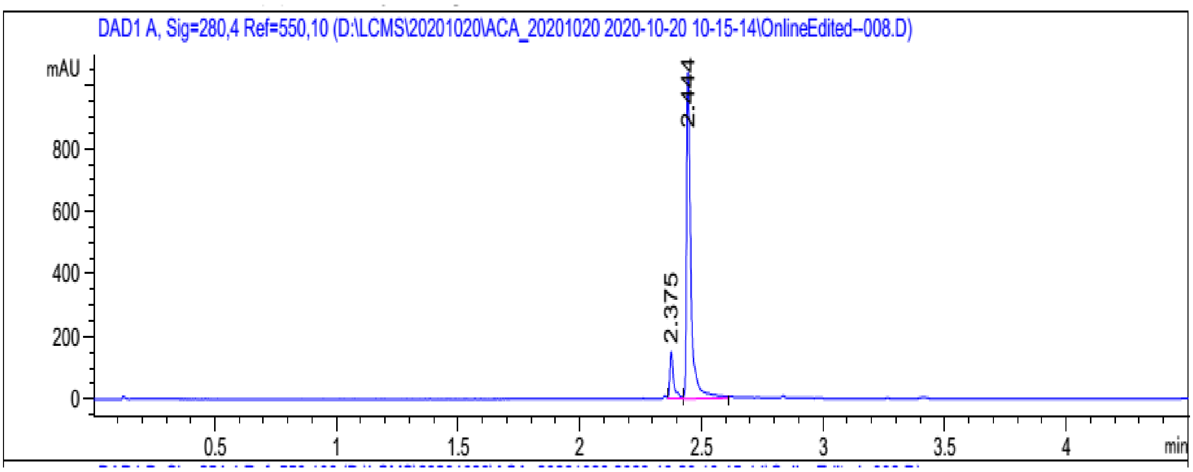

S62: HPLC chromatogram of **15**

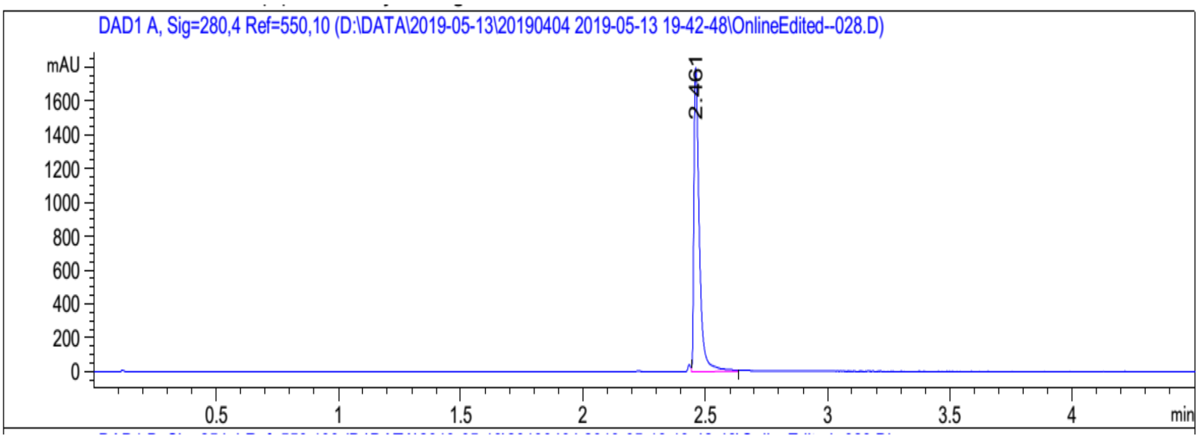

S63: HPLC chromatogram of **16**

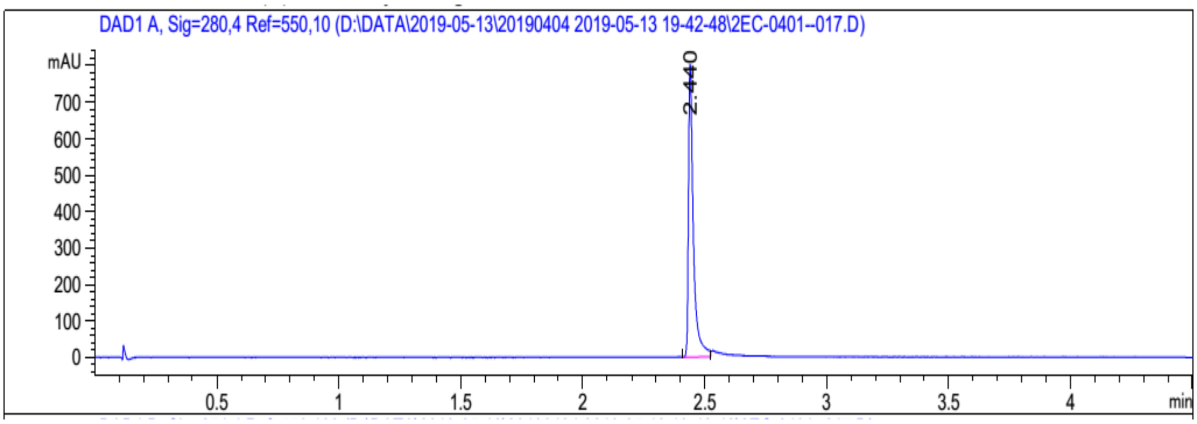

S64: HPLC chromatogram of **17**

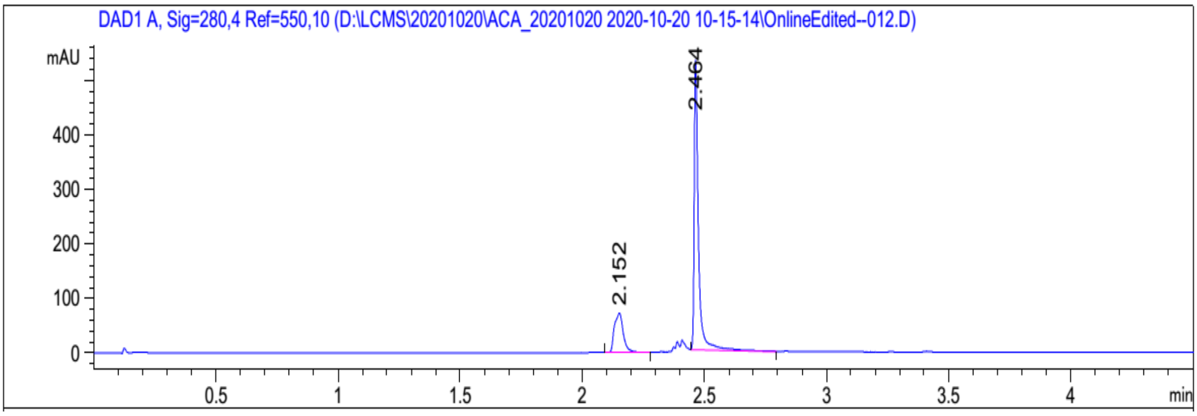

S65: HPLC chromatogram of **18**

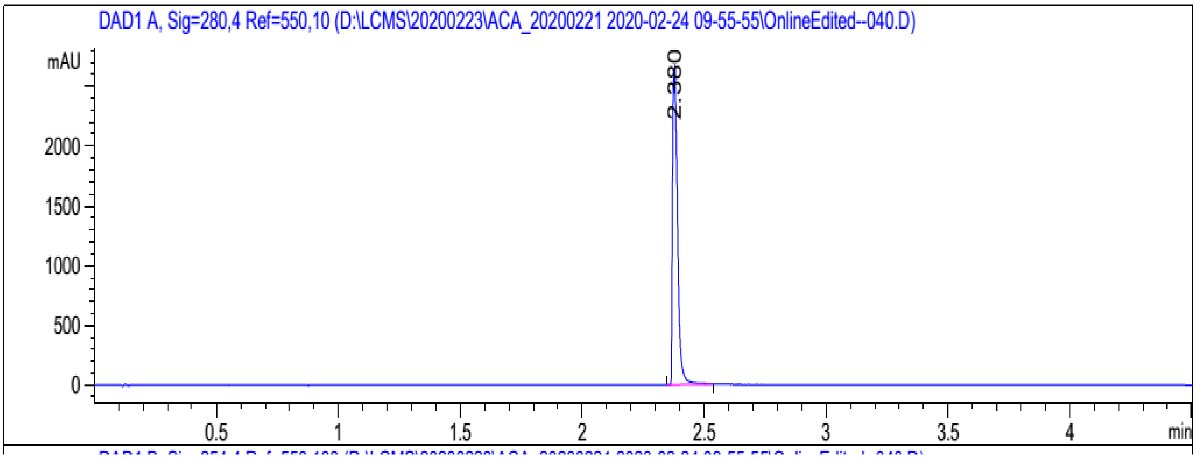

S66: HPLC chromatogram of **19**

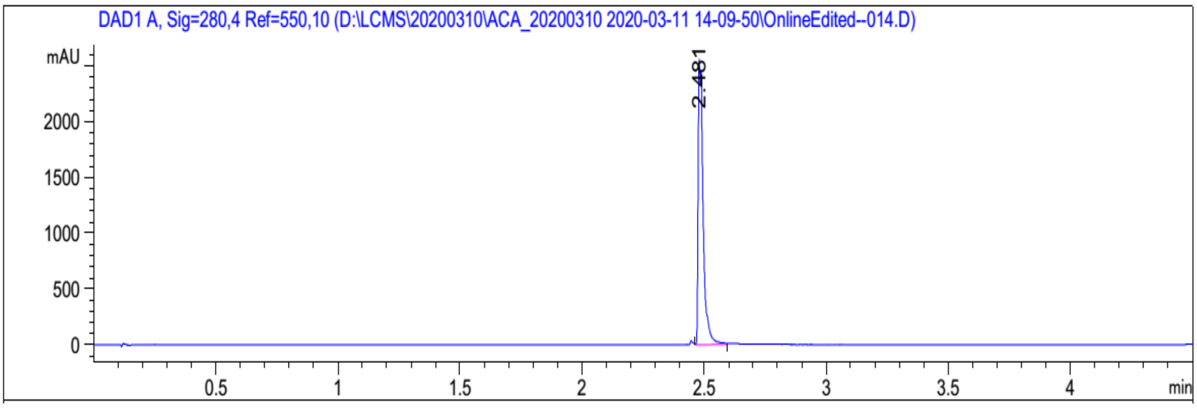

S67: HPLC chromatogram of **20**

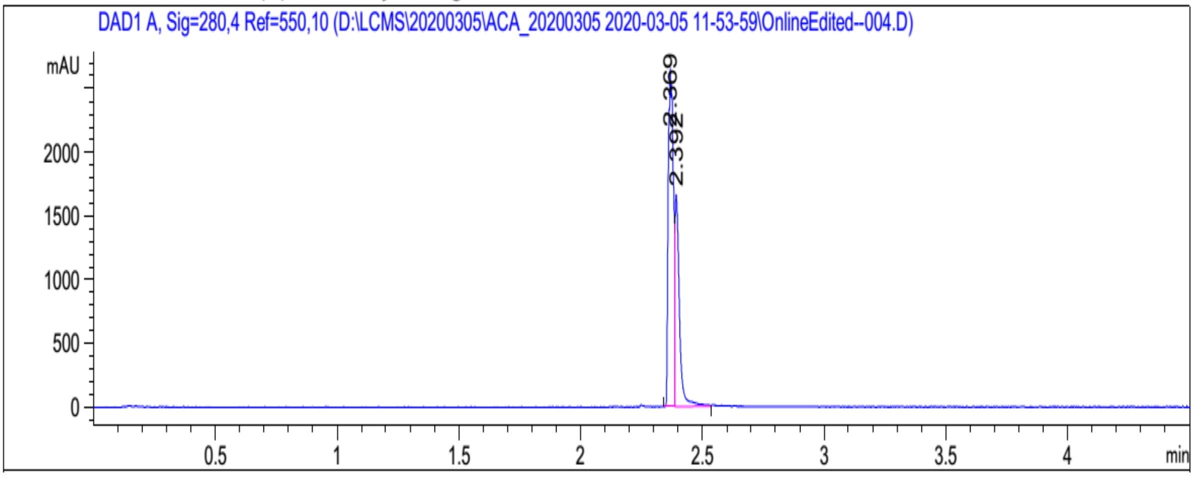

S68: HPLC chromatogram of **21**

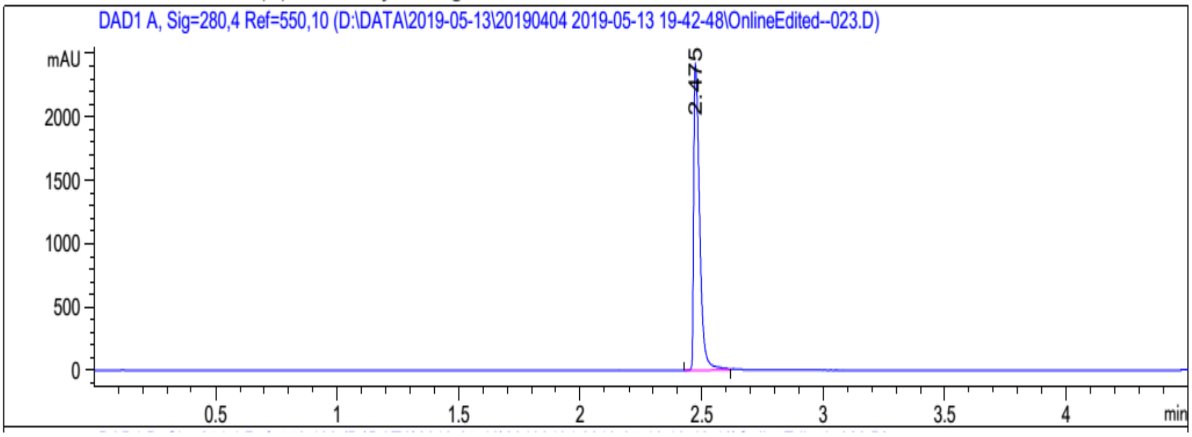

S69: HPLC chromatogram of **22**

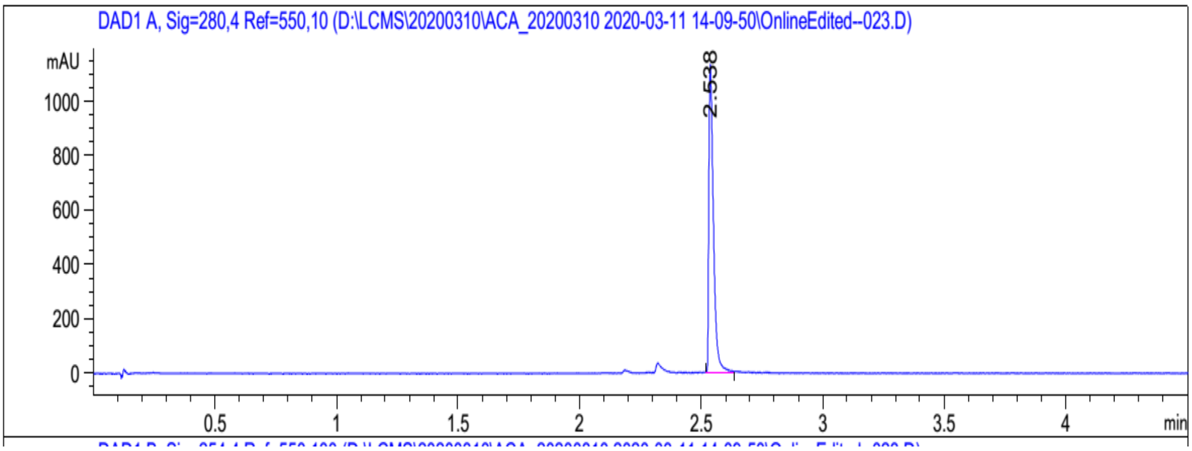

S70: HPLC chromatogram of **23**

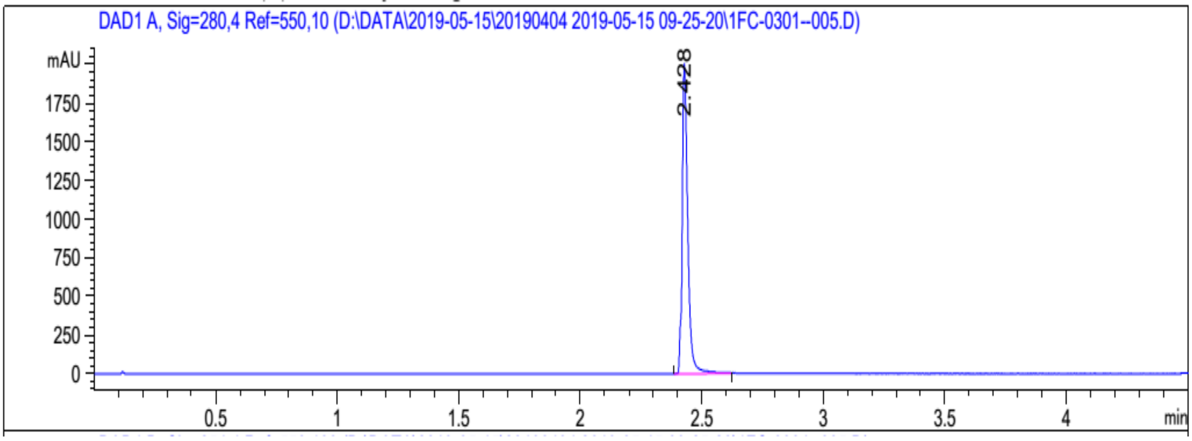

S71: HPLC chromatogram of **24**

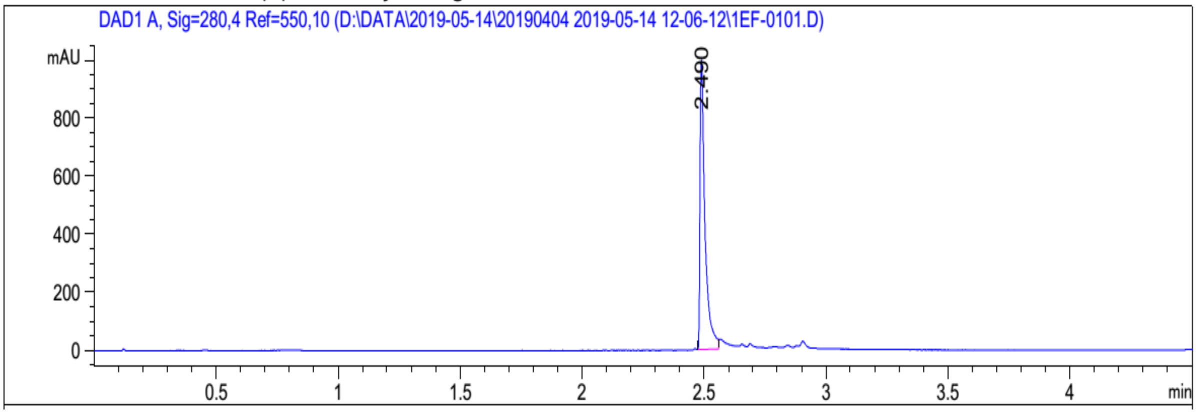

S72: HPLC chromatogram of **25**

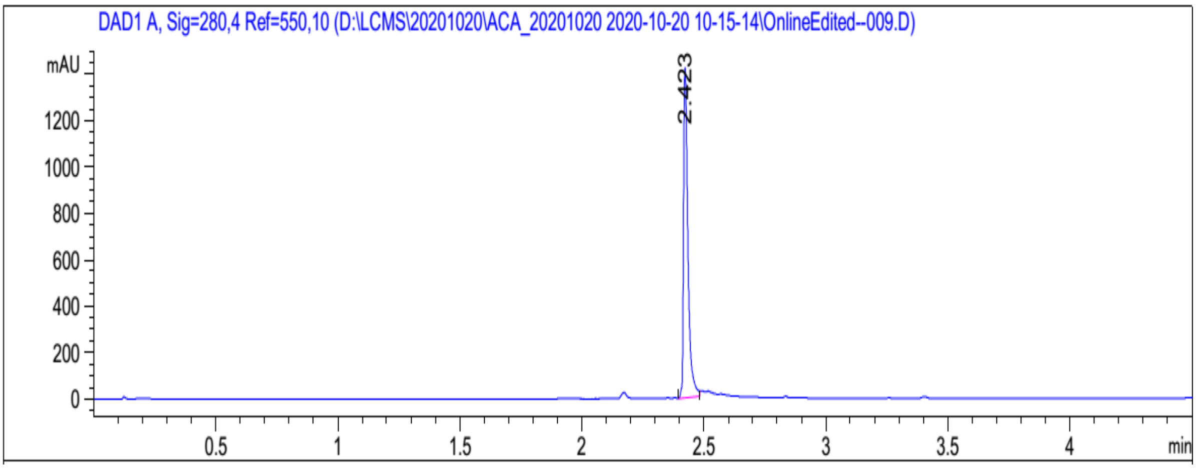

S73: HPLC chromatogram of **26**

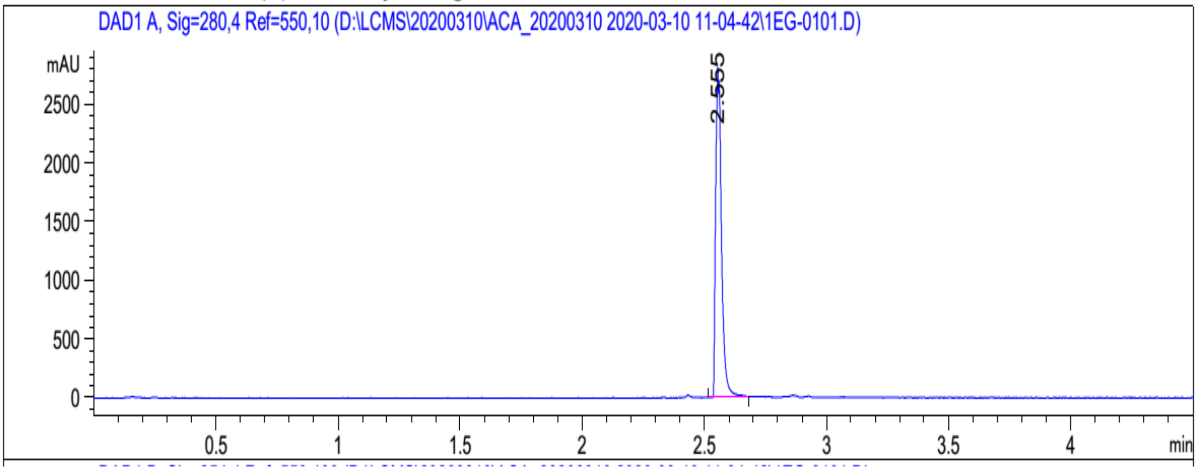

S74: HPLC chromatogram of **27**

MS Spectra of representative compounds

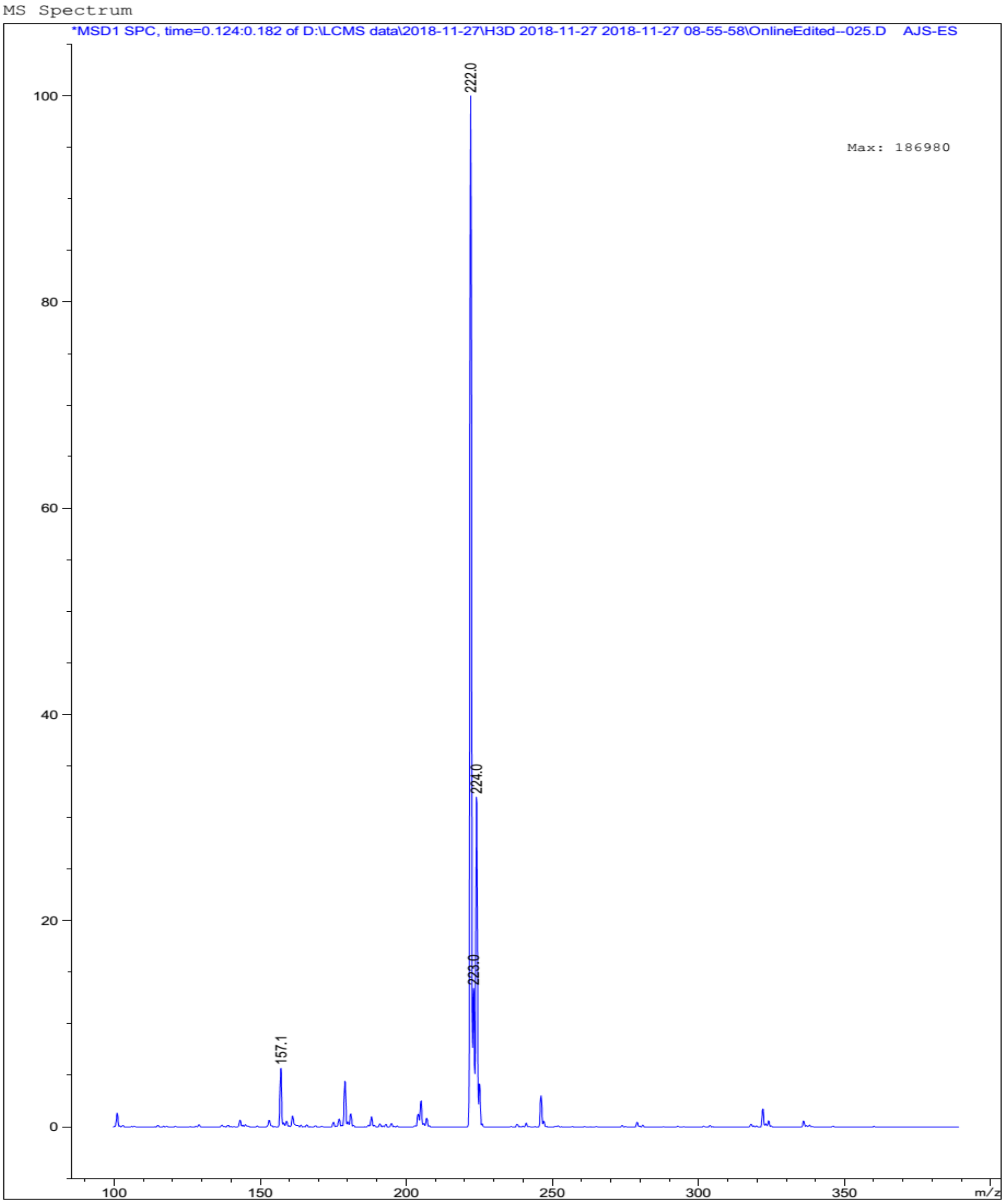

S75: HPLC-MS of **2**

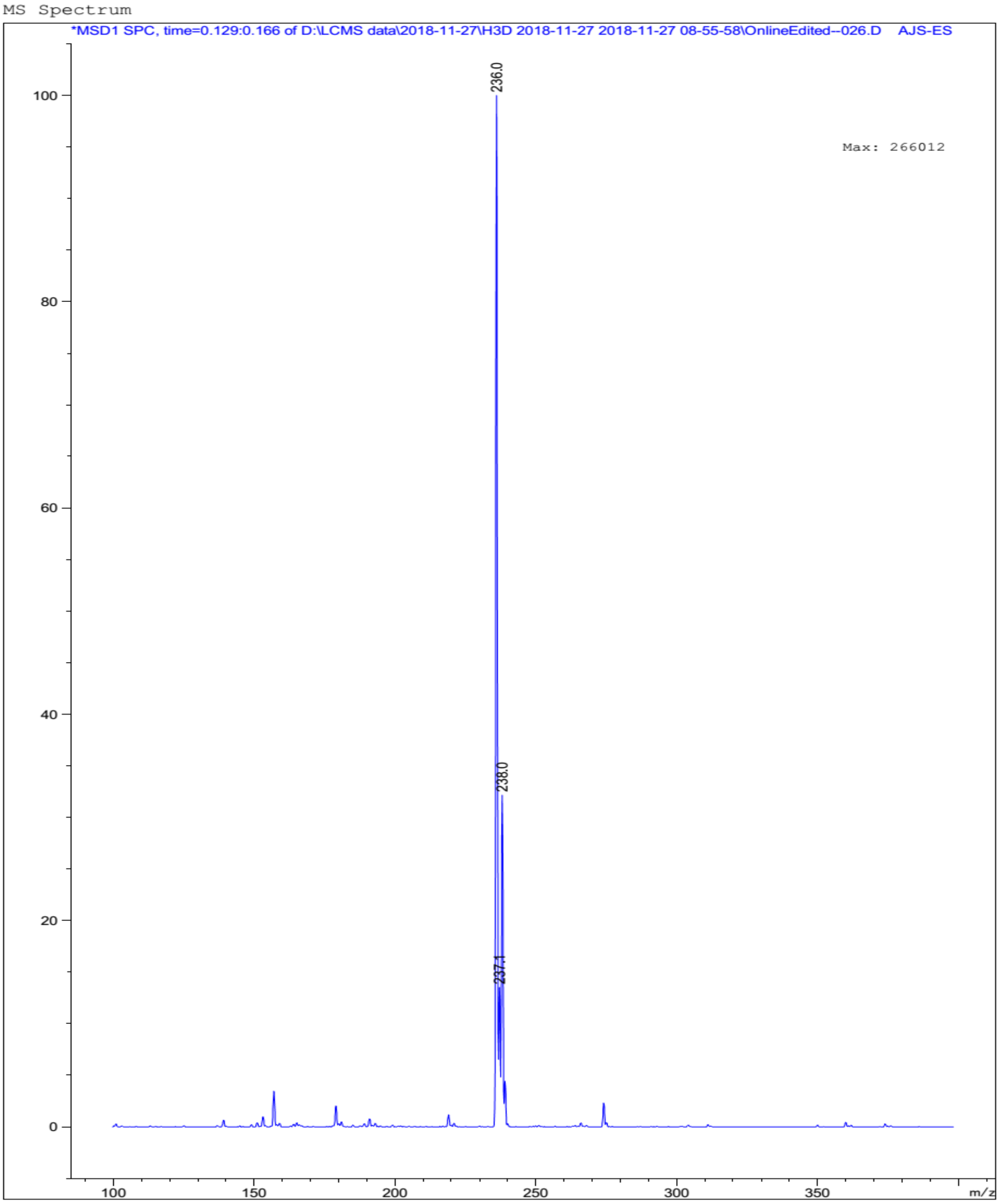

S76: HPLC-MS of **3**

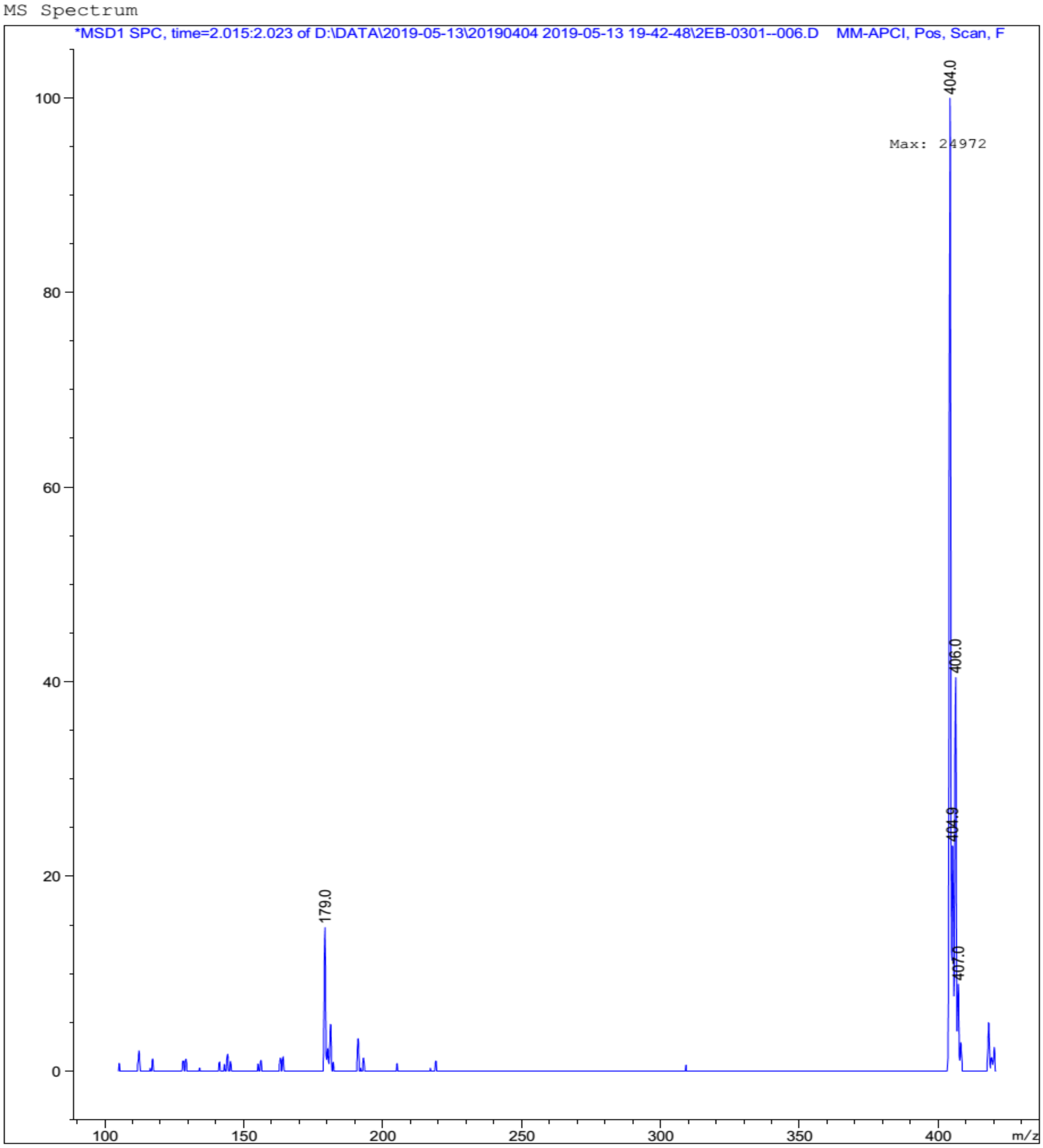

S77: HPLC-MS of **4**

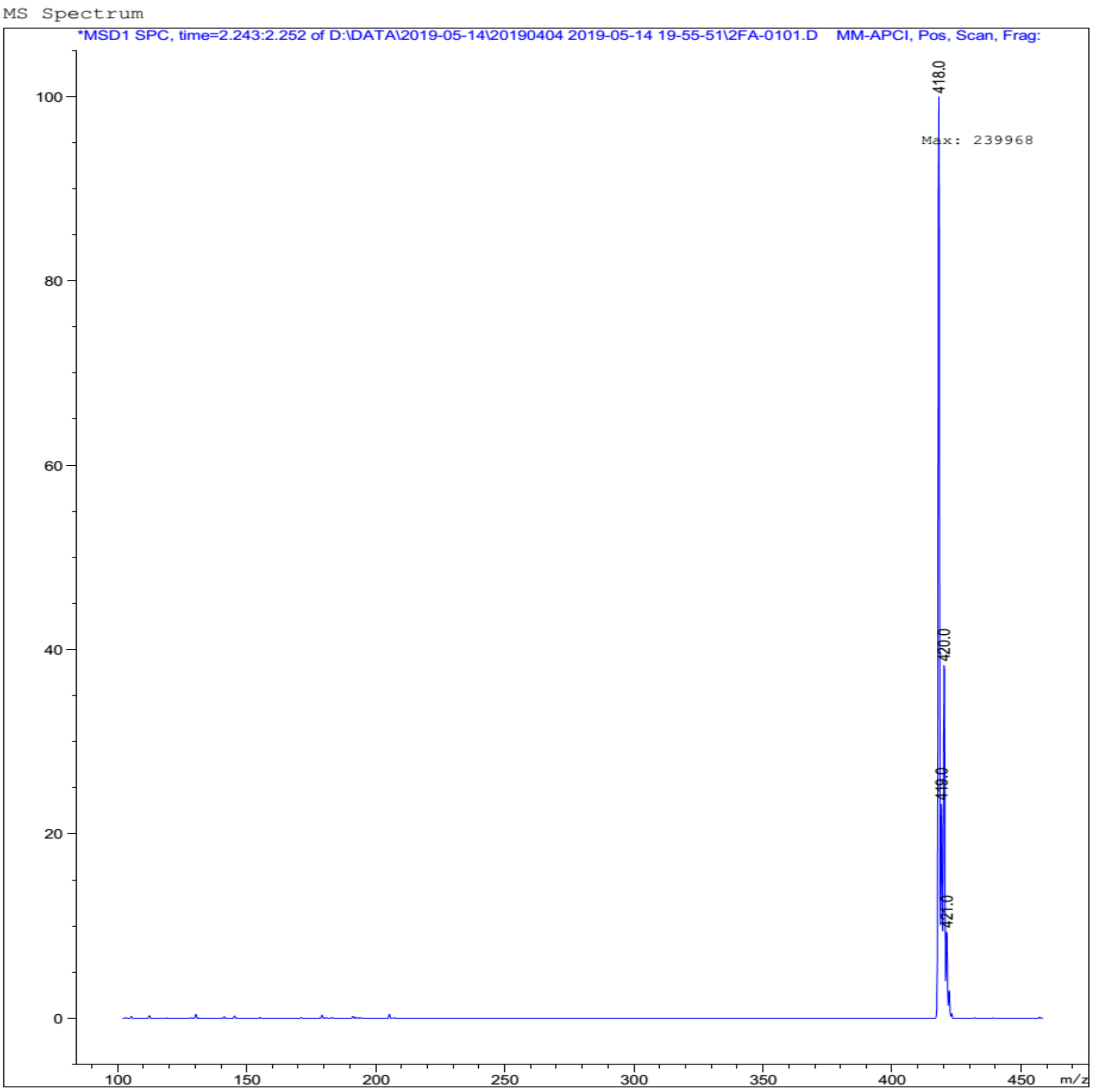

S78: HPLC-MS of **5**

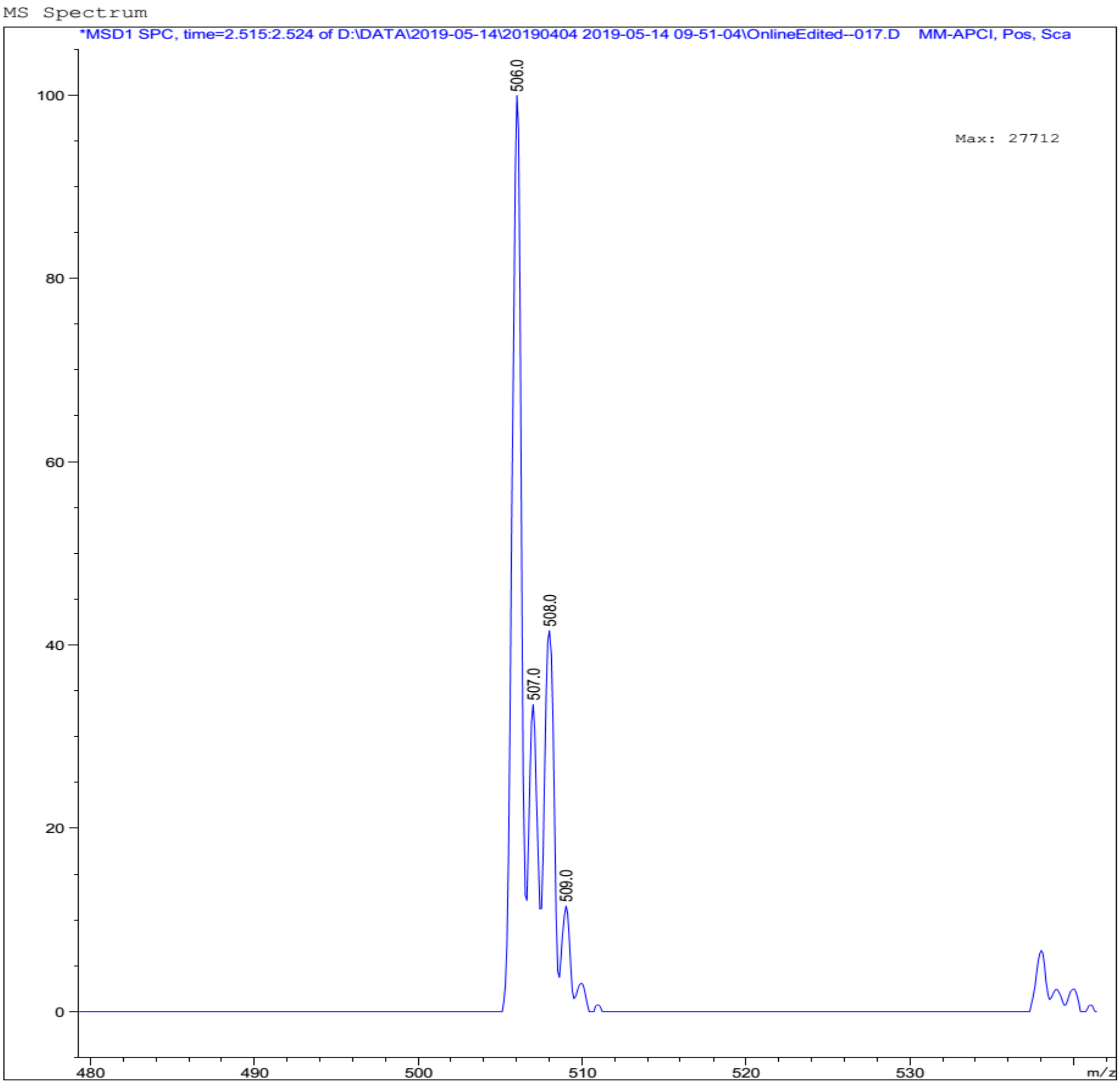

S79: HPLC-MS of **6**

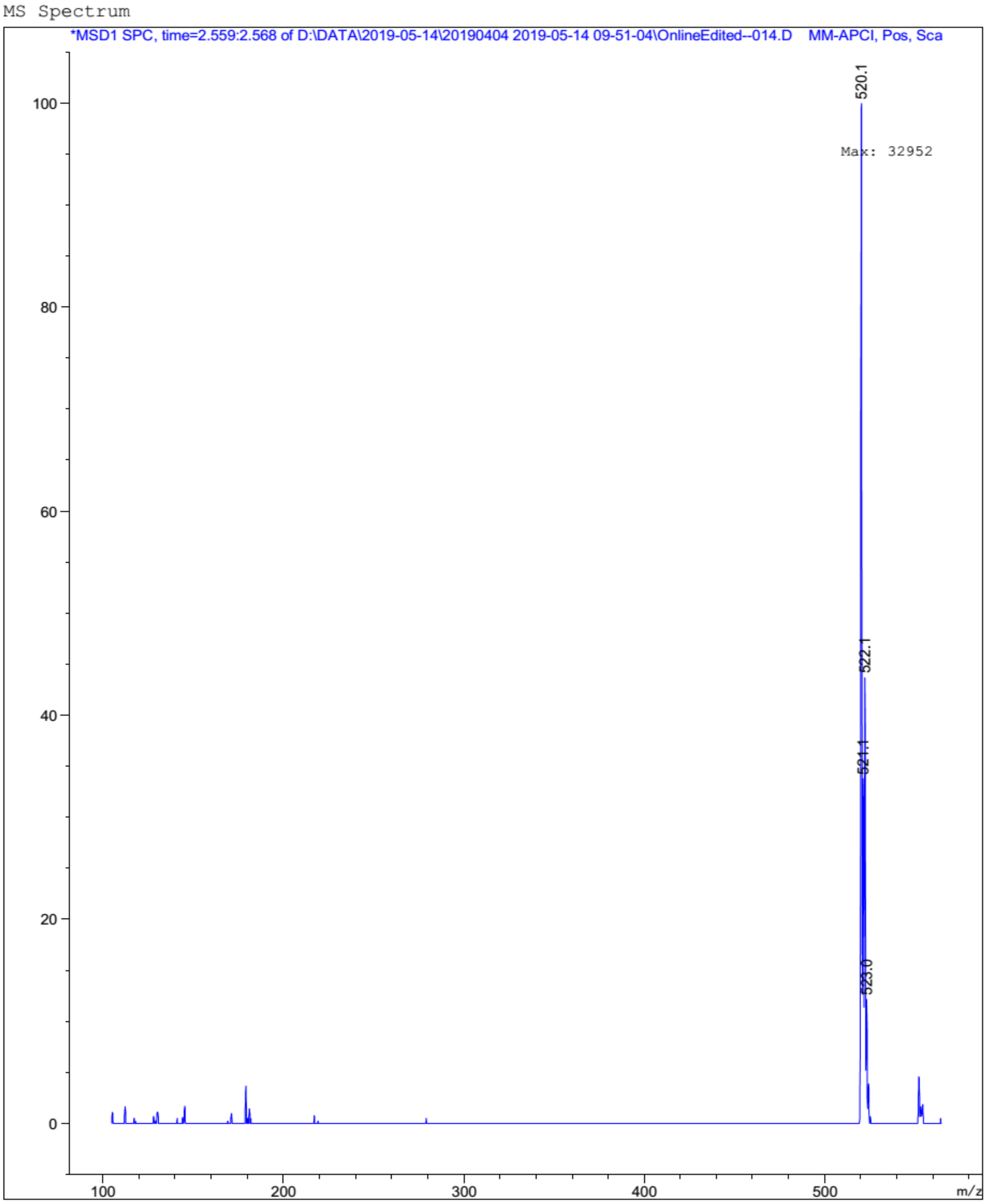

S80: HPLC-MS of **7**

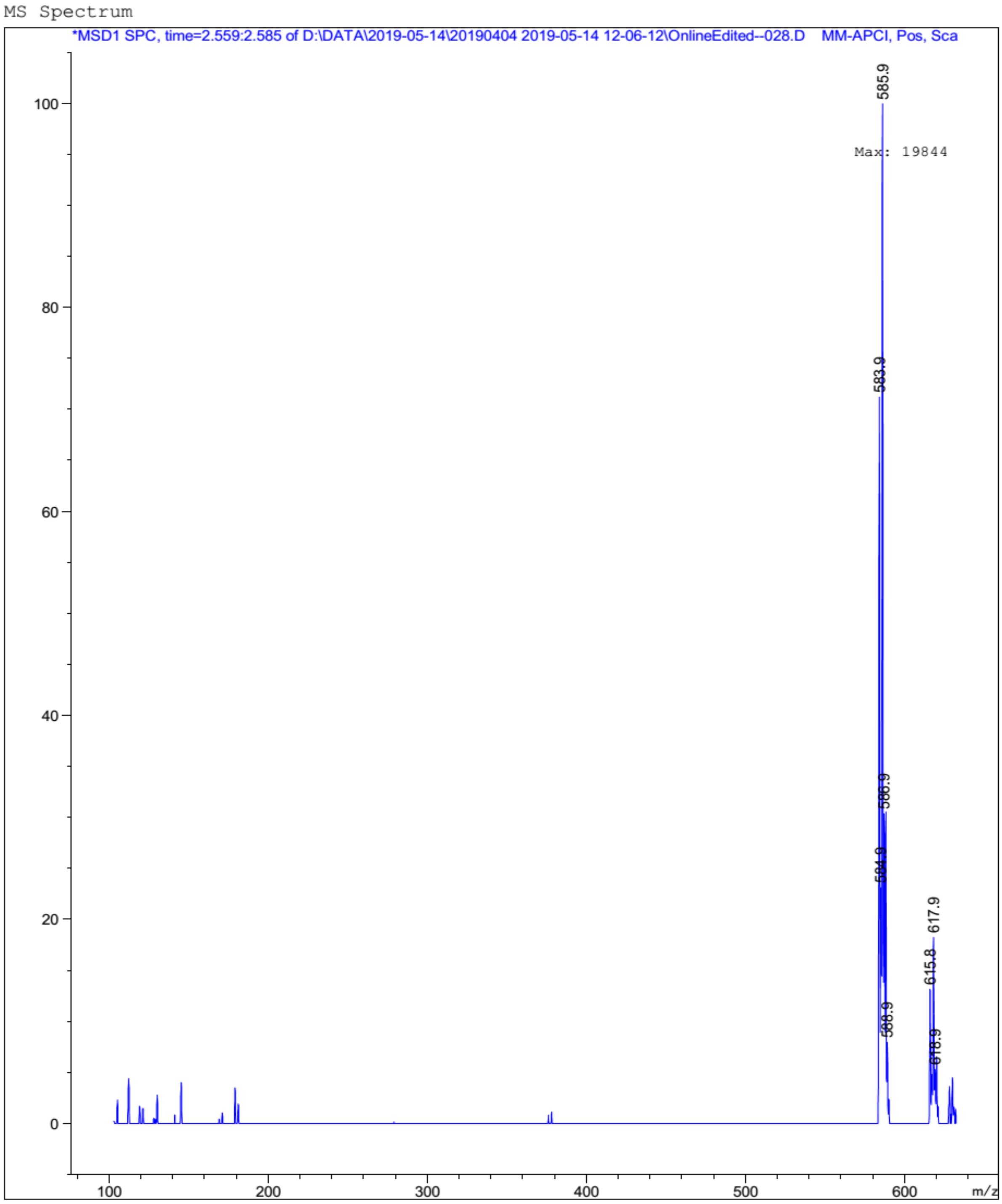

S81: HPLC-MS of **8**

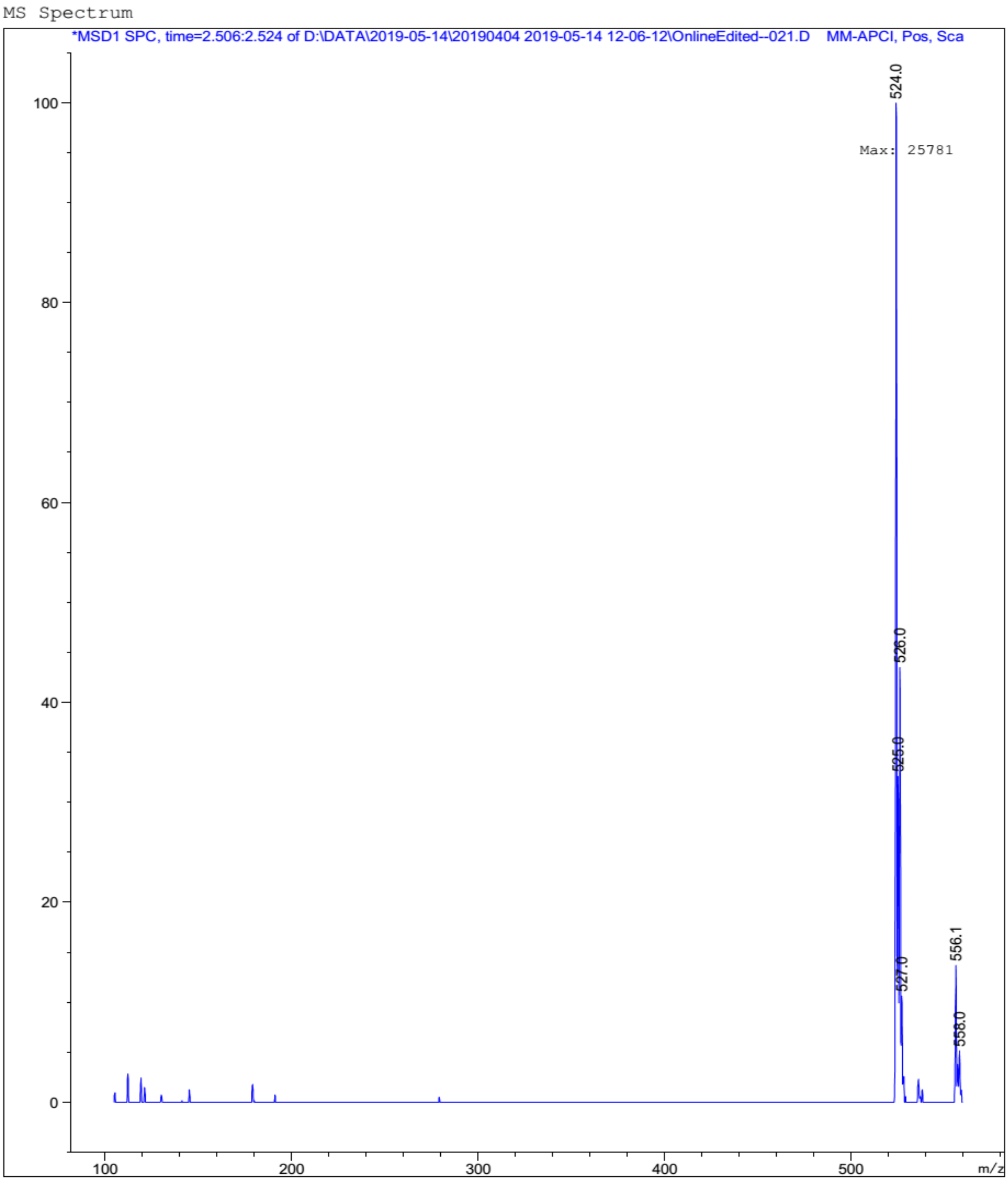

S82: HPLC-MS of **9**

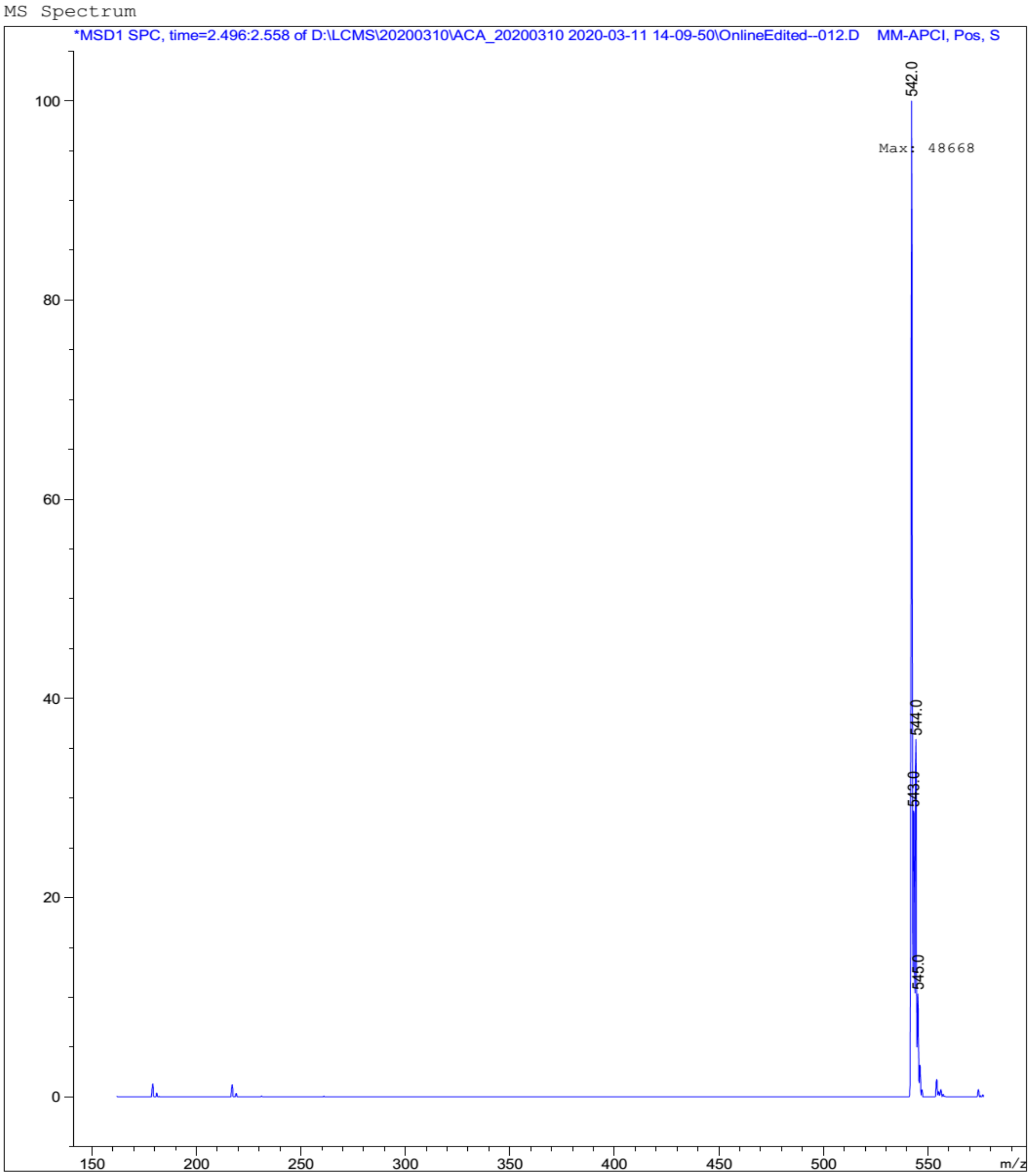

S83: HPLC-MS of **10**

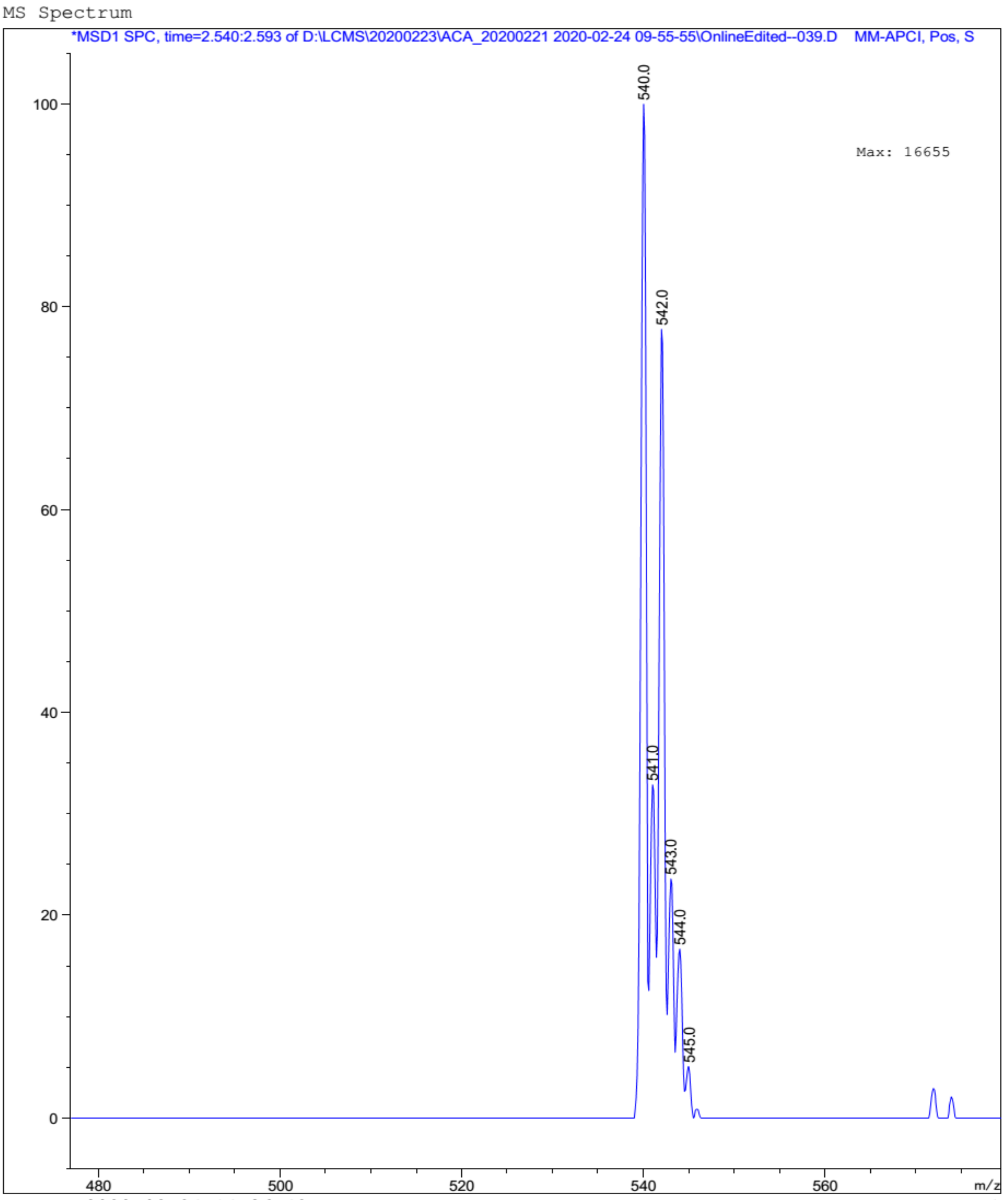

S84: HPLC-MS of **11**

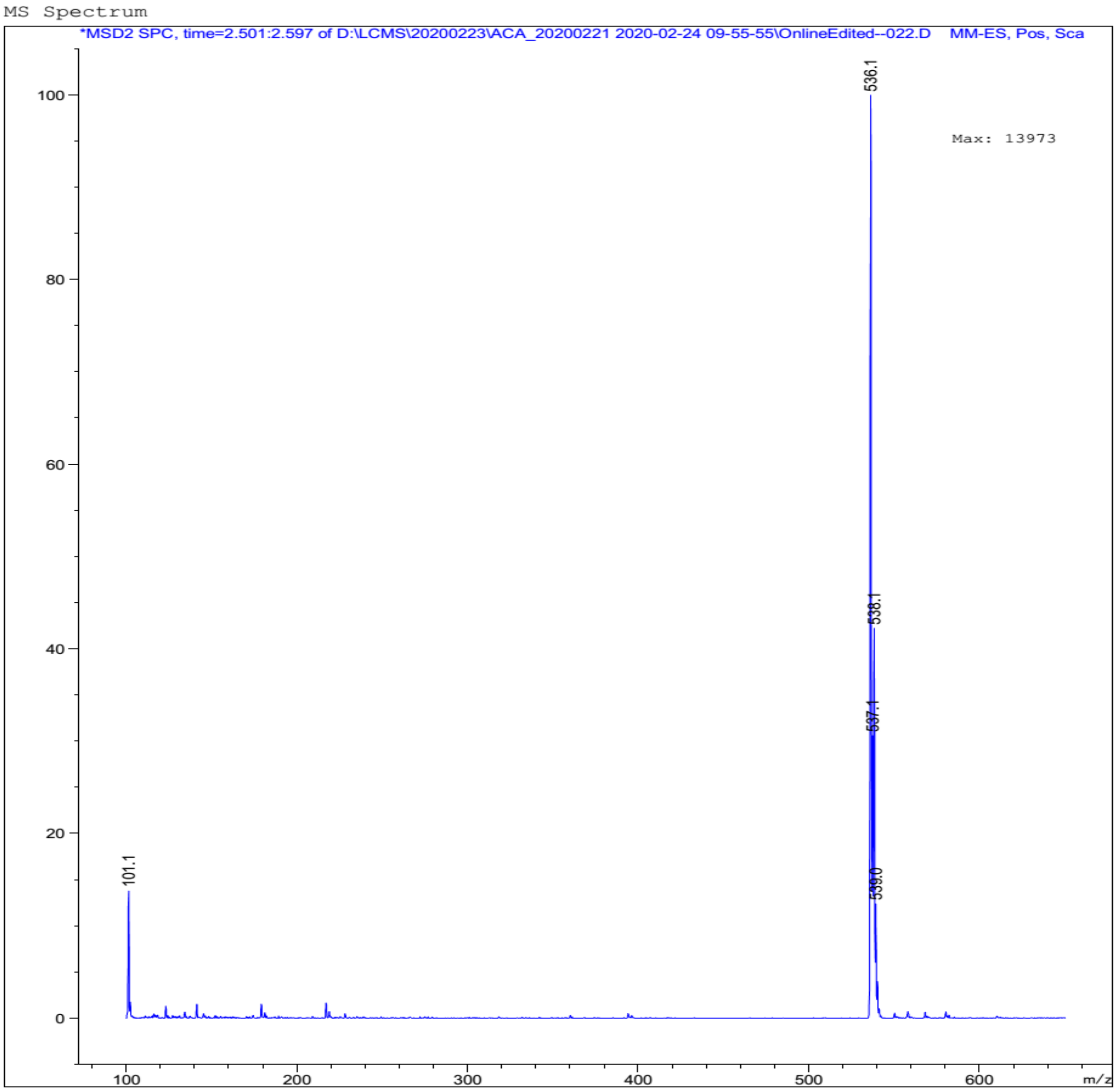

S85: HPLC-MS of **12**

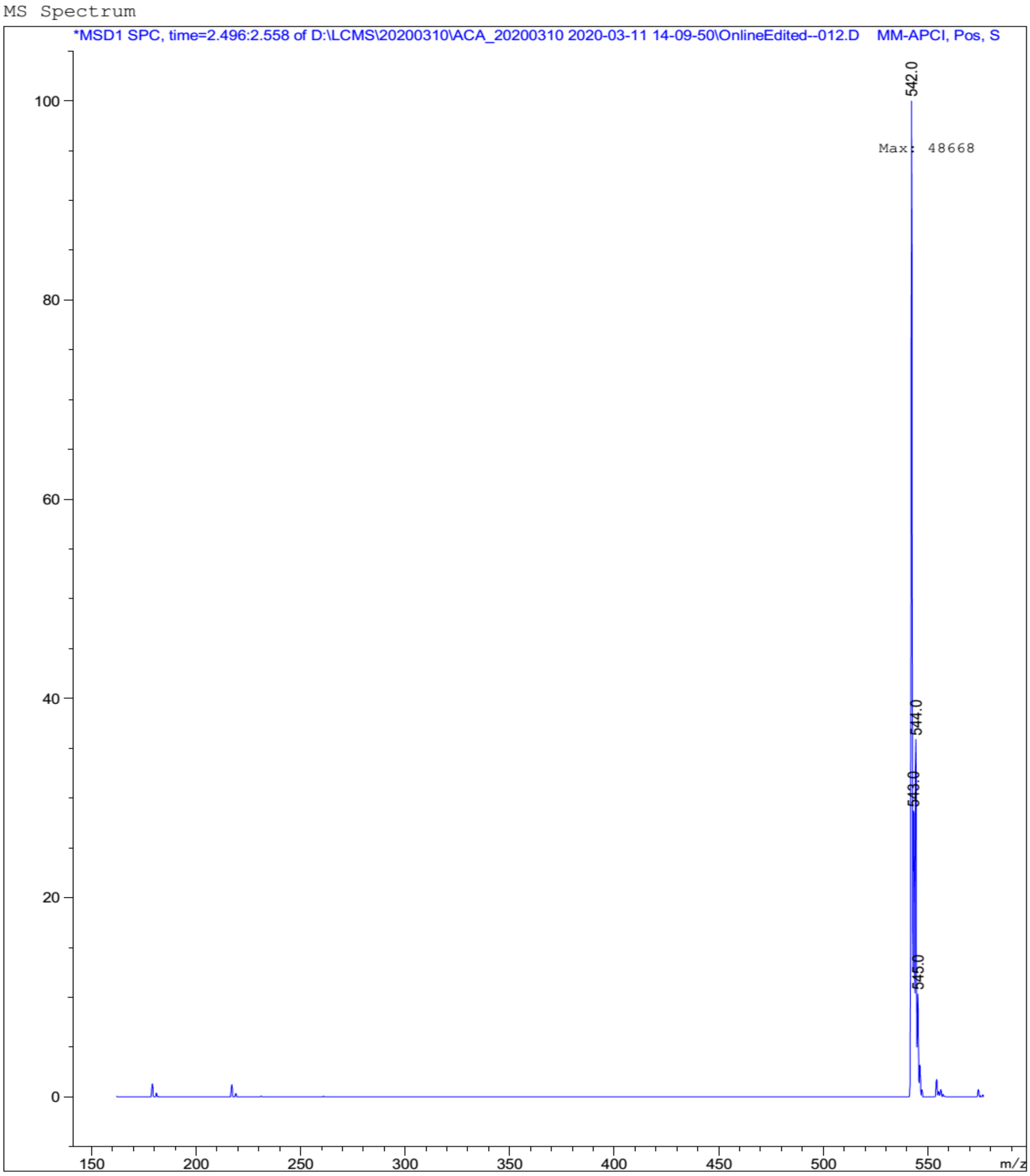

S86: HPLC-MS of **13**

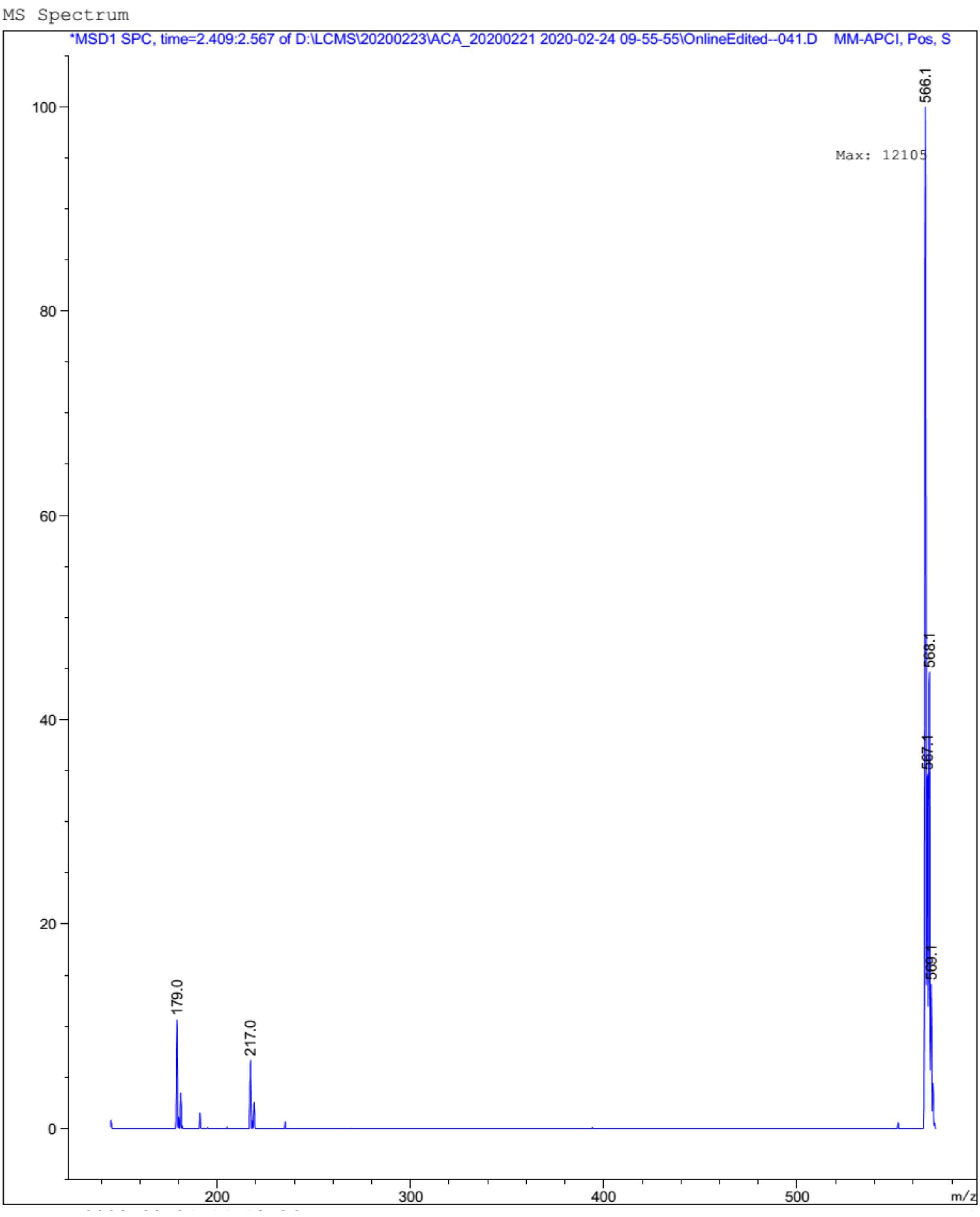

S87: HPLC-MS of **14**

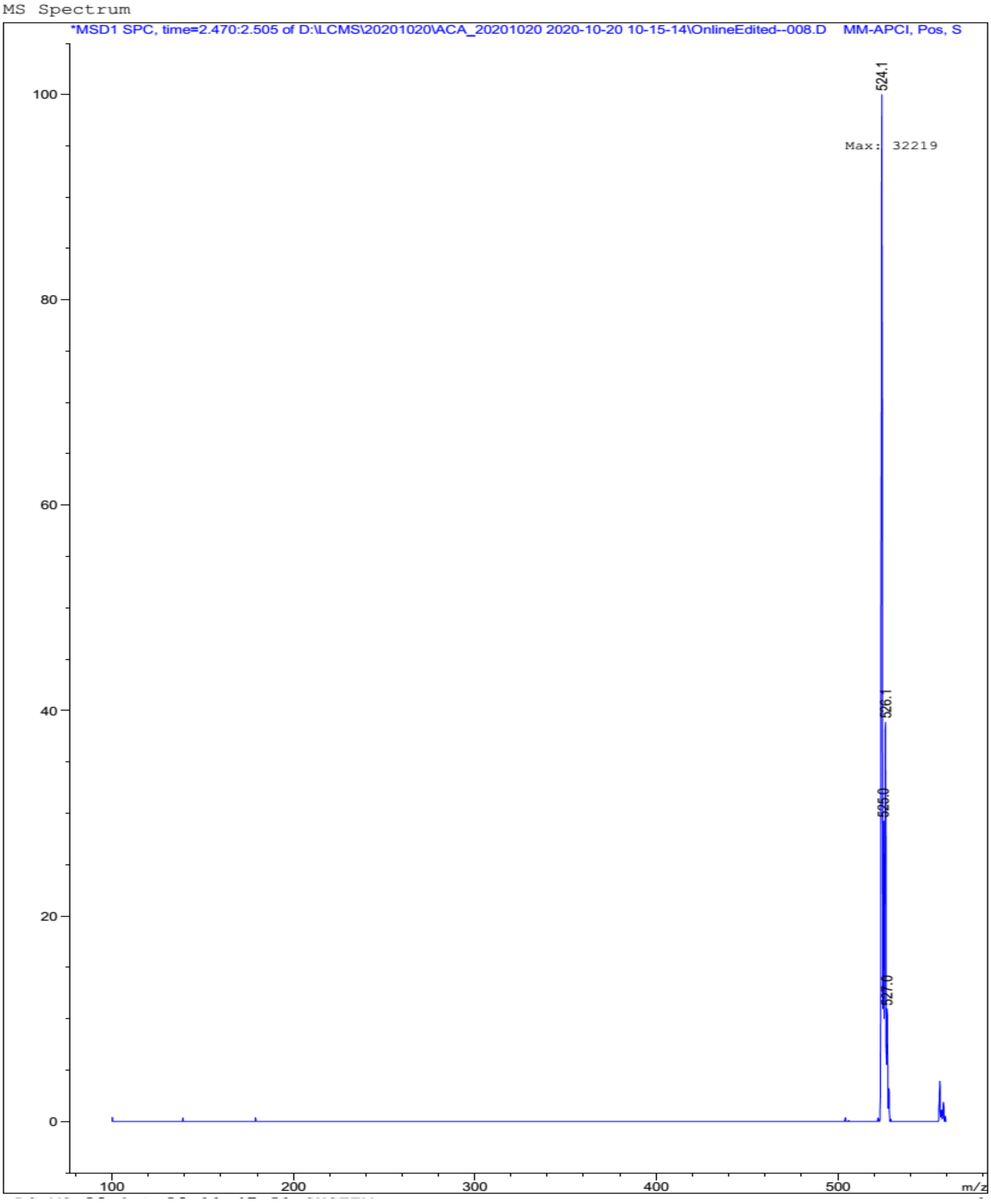

S88: HPLC-MS of **15**

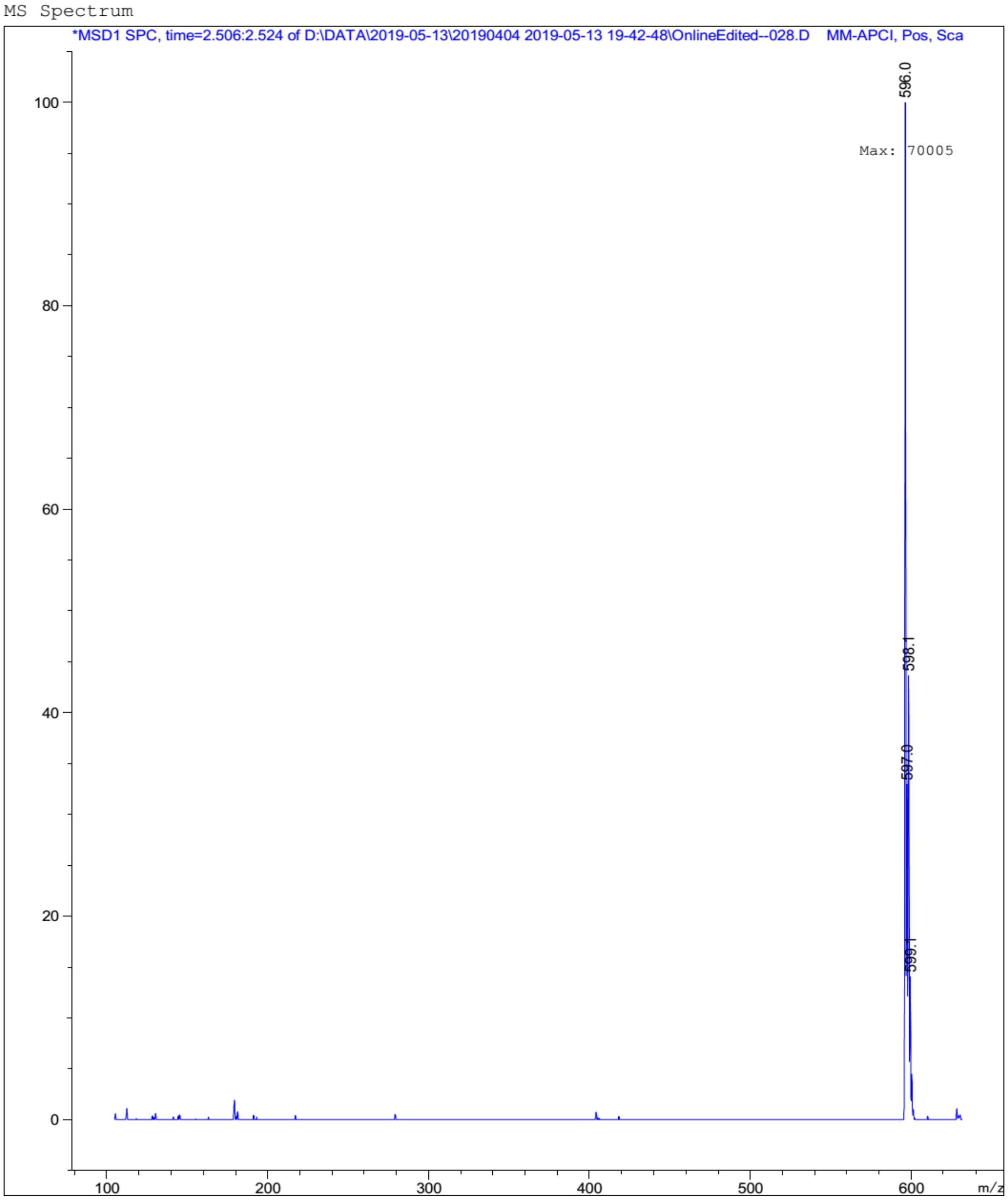

S89: HPLC-MS of **16**

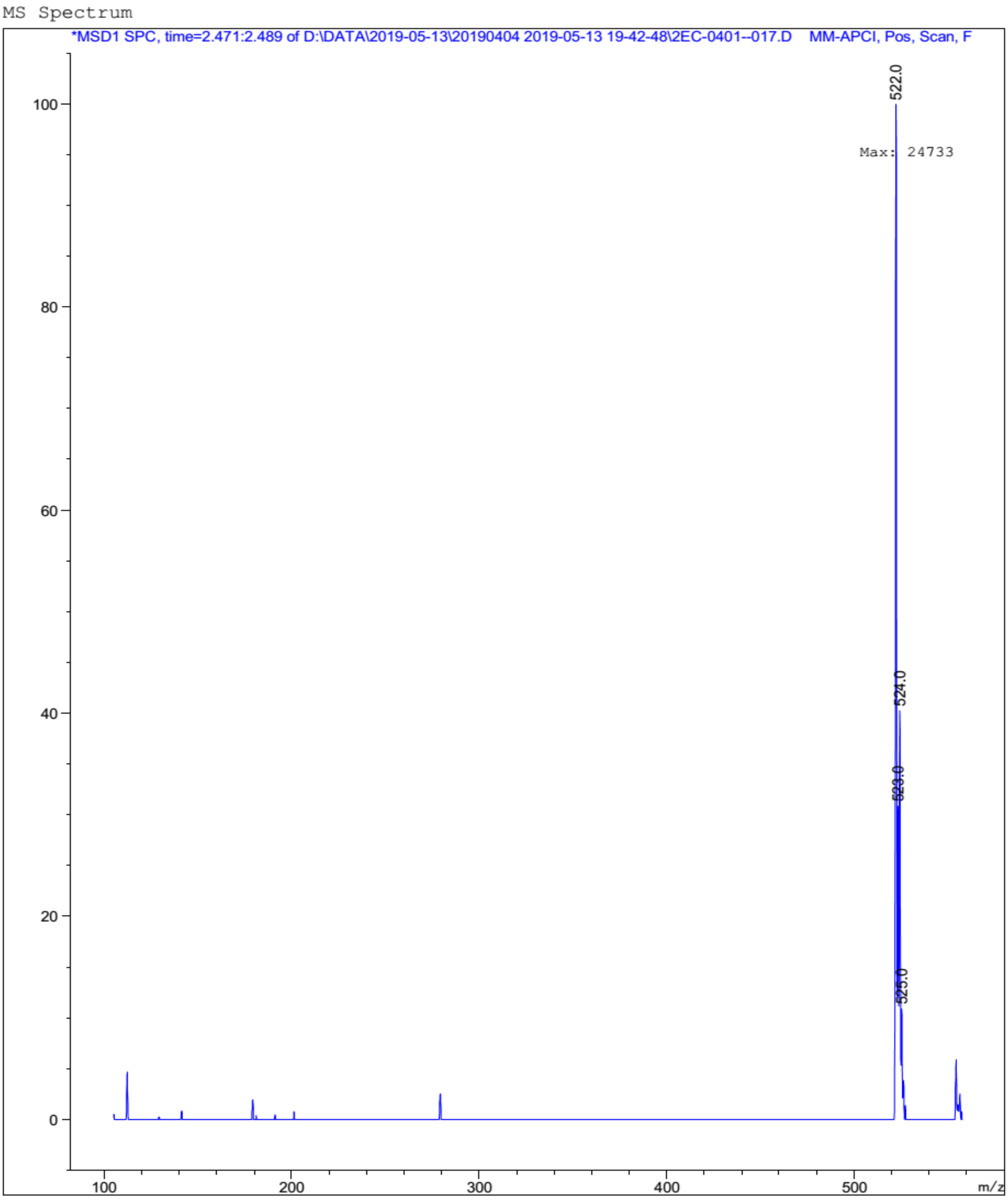

S90: HPLC-MS of **17**

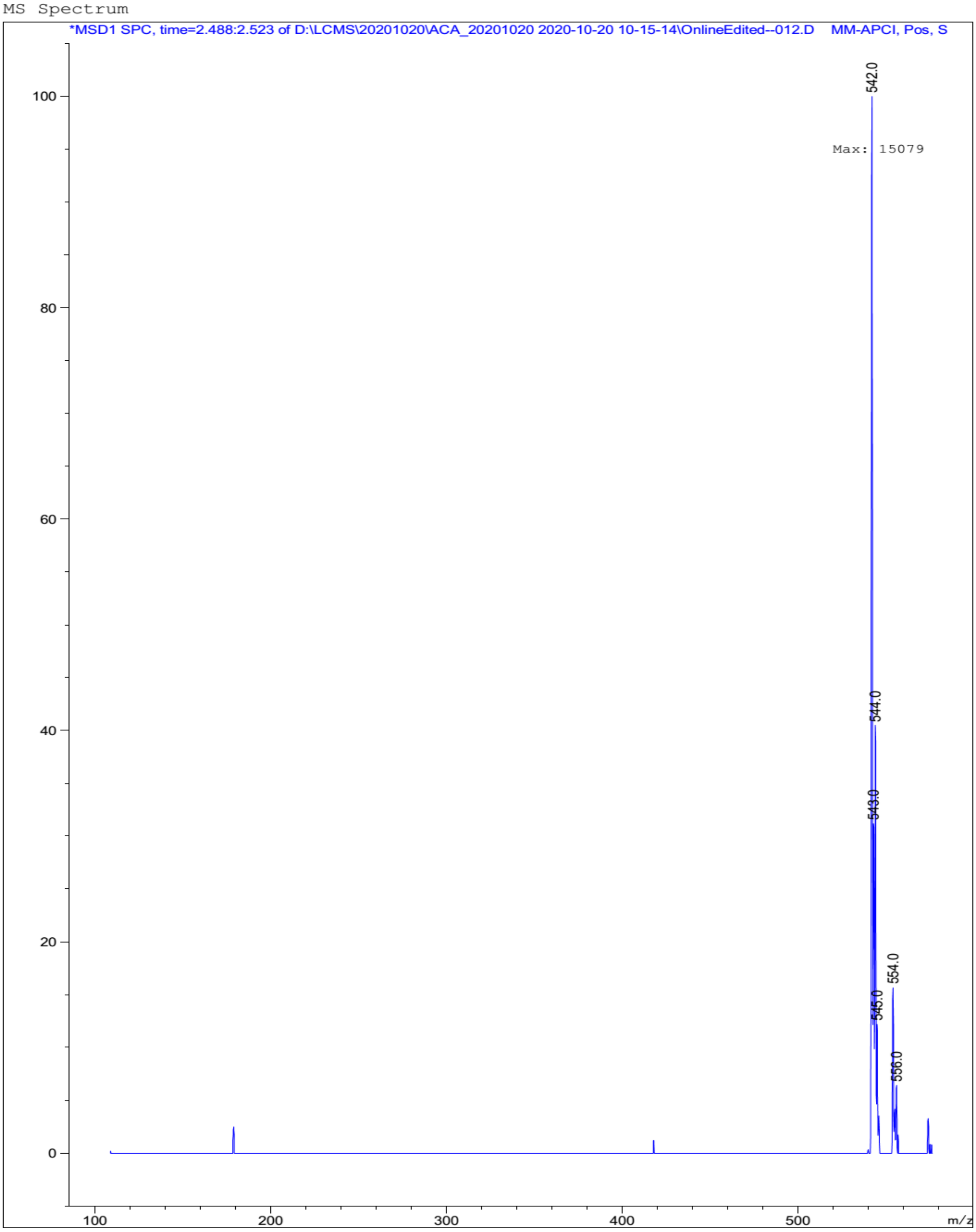

S91: HPLC-MS of **18**

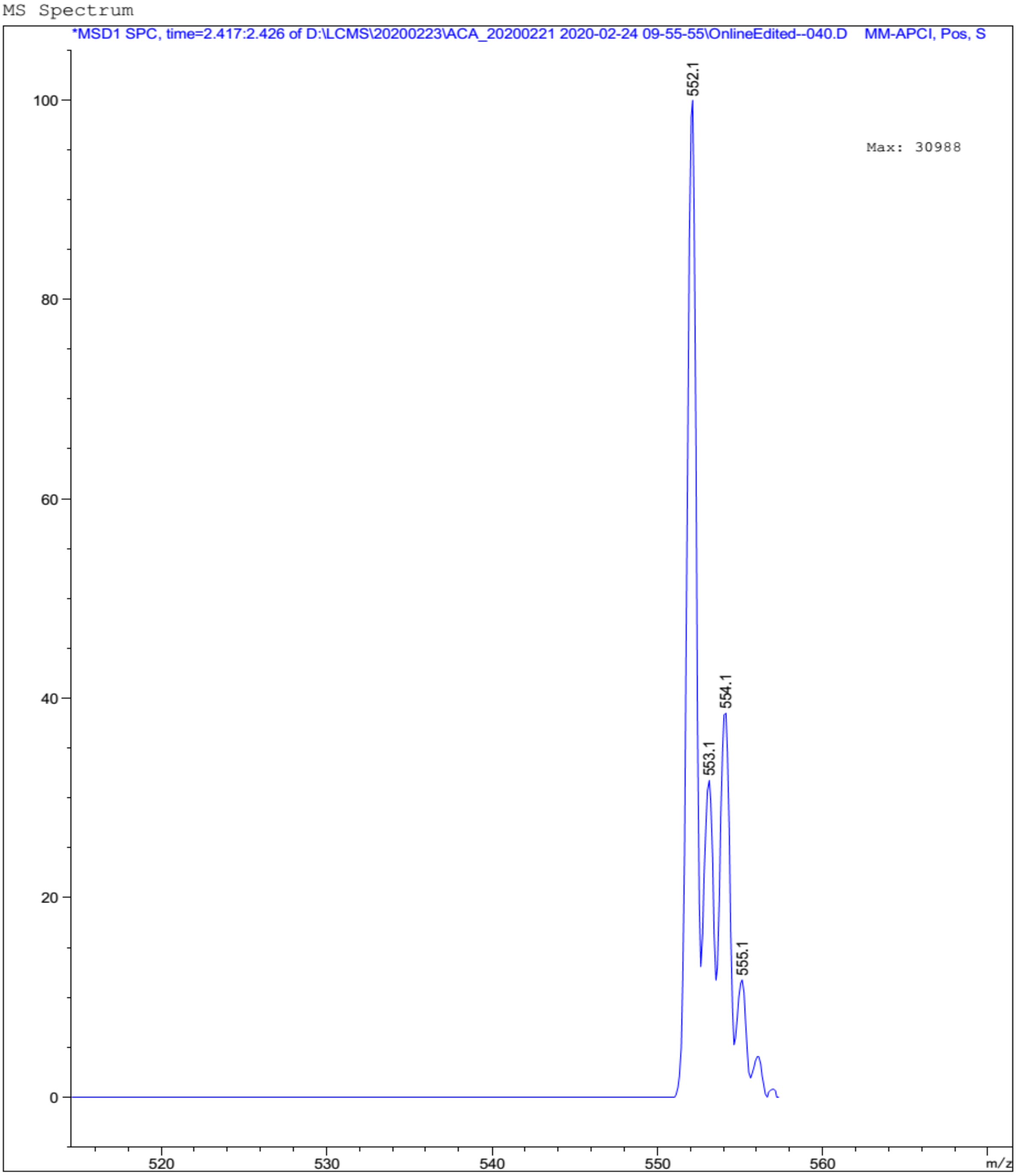

S92: HPLC-MS of **19**

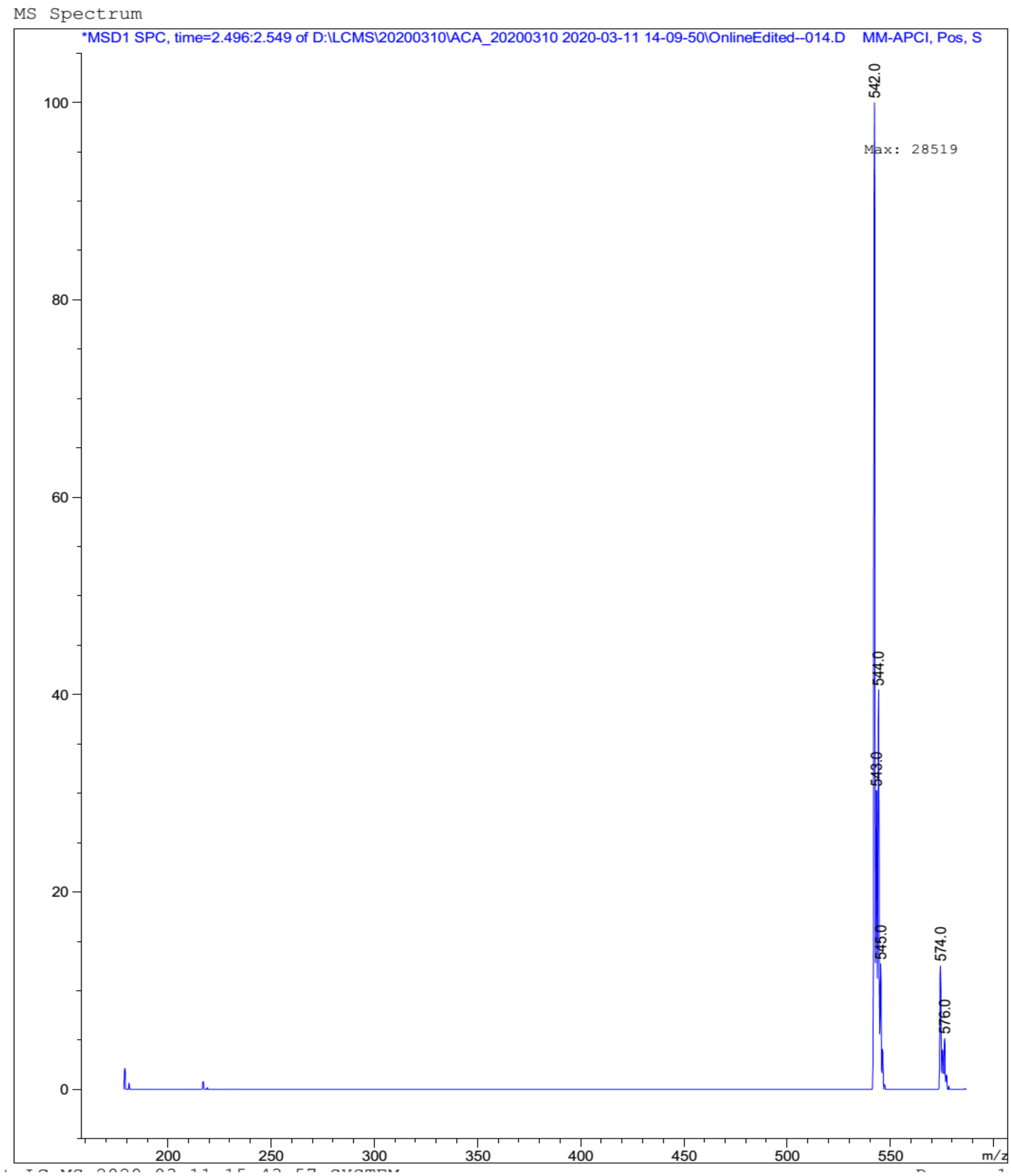

S93: HPLC-MS of **20**

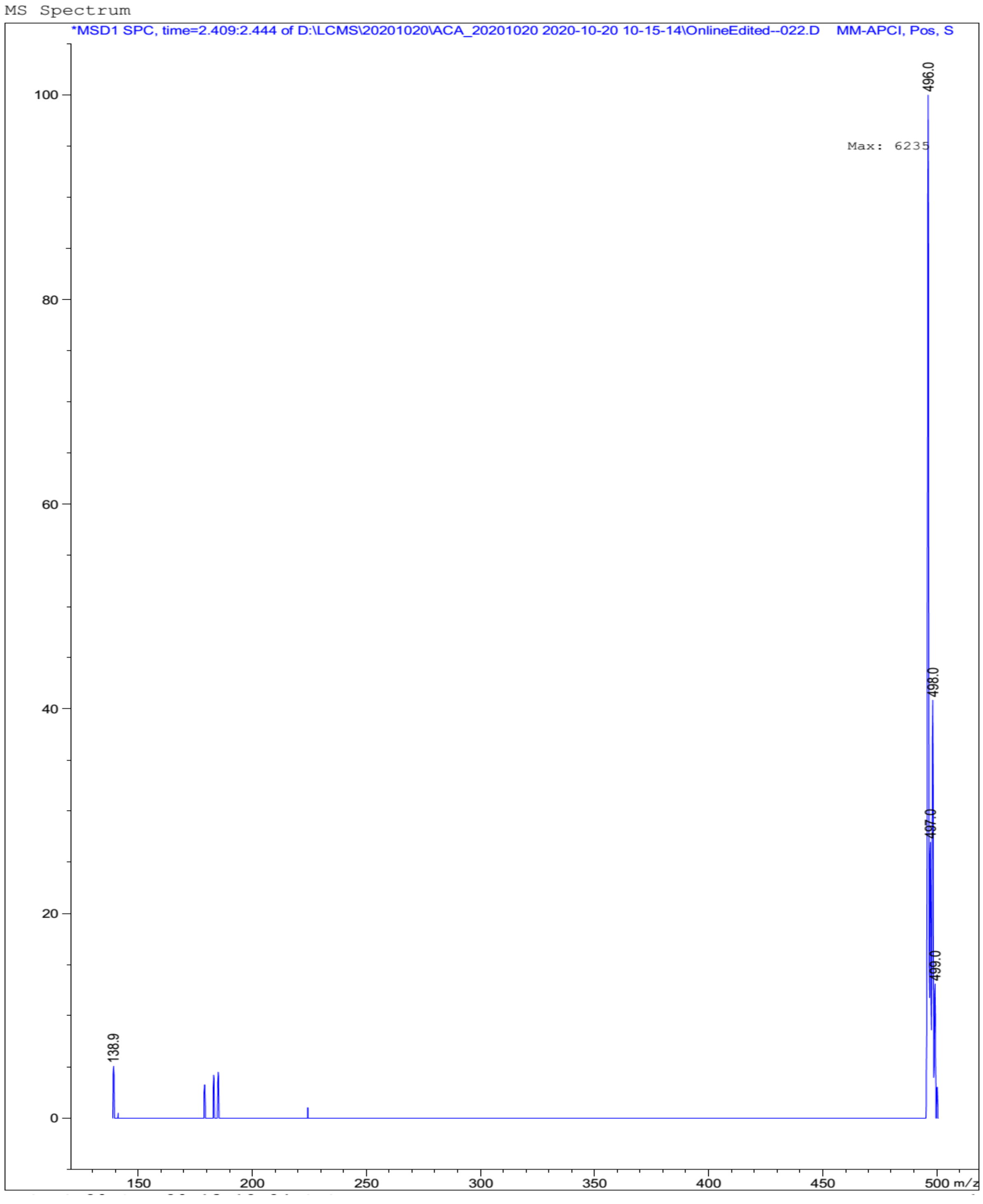

S94: HPLC-MS of **21**

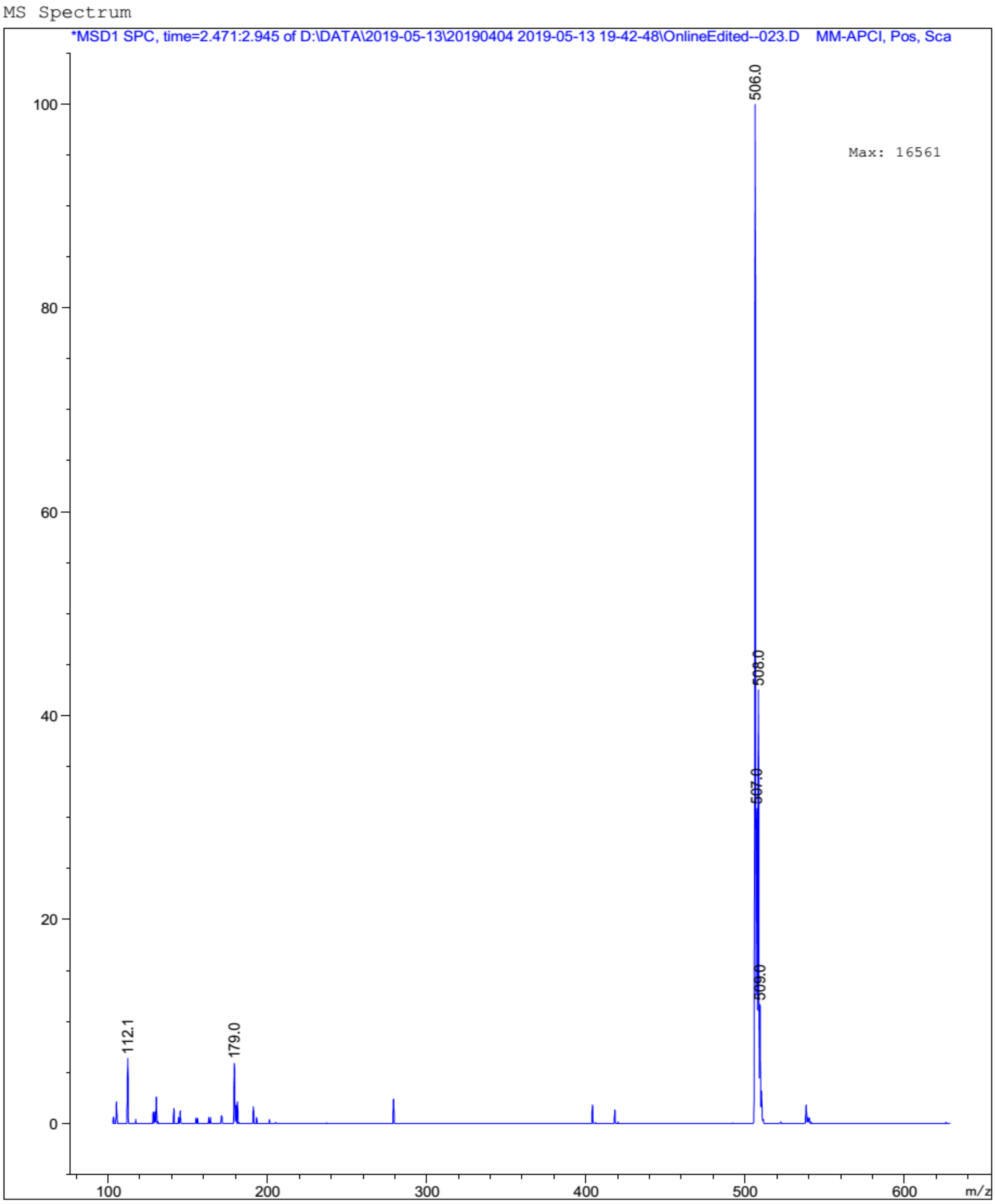

S95: HPLC-MS of **22**

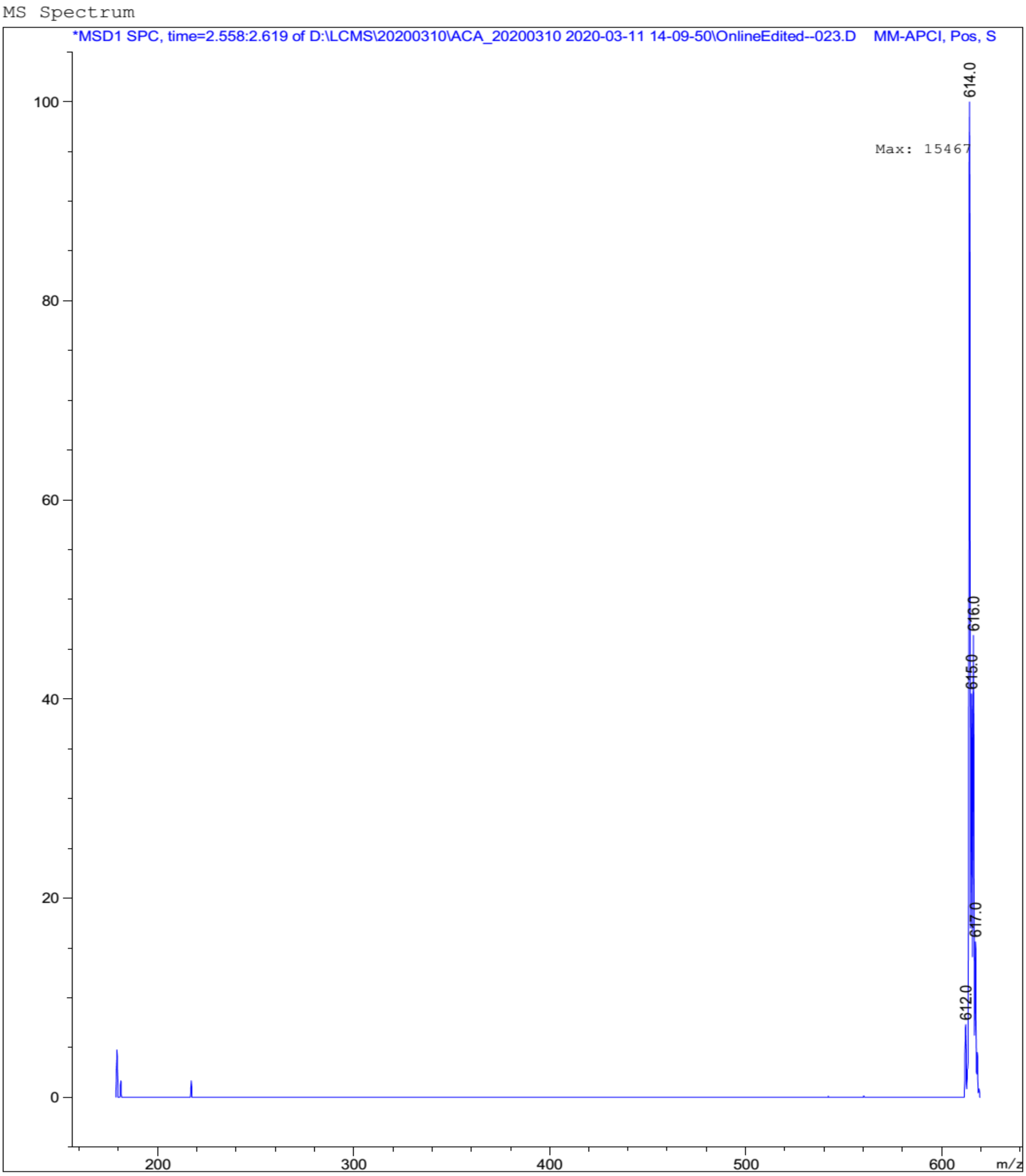

S96: HPLC-MS of **23**

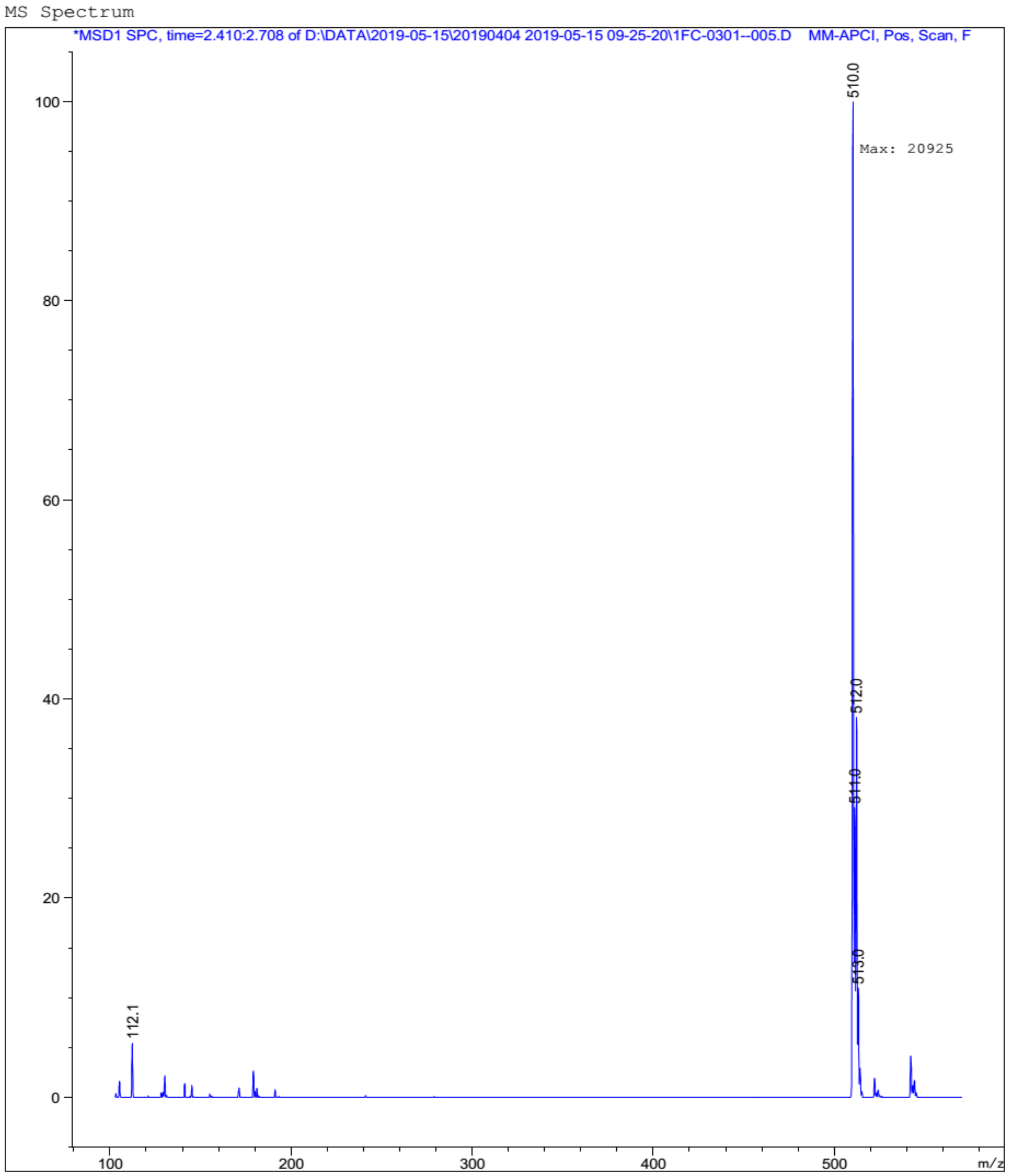

S97: HPLC-MS of **24**

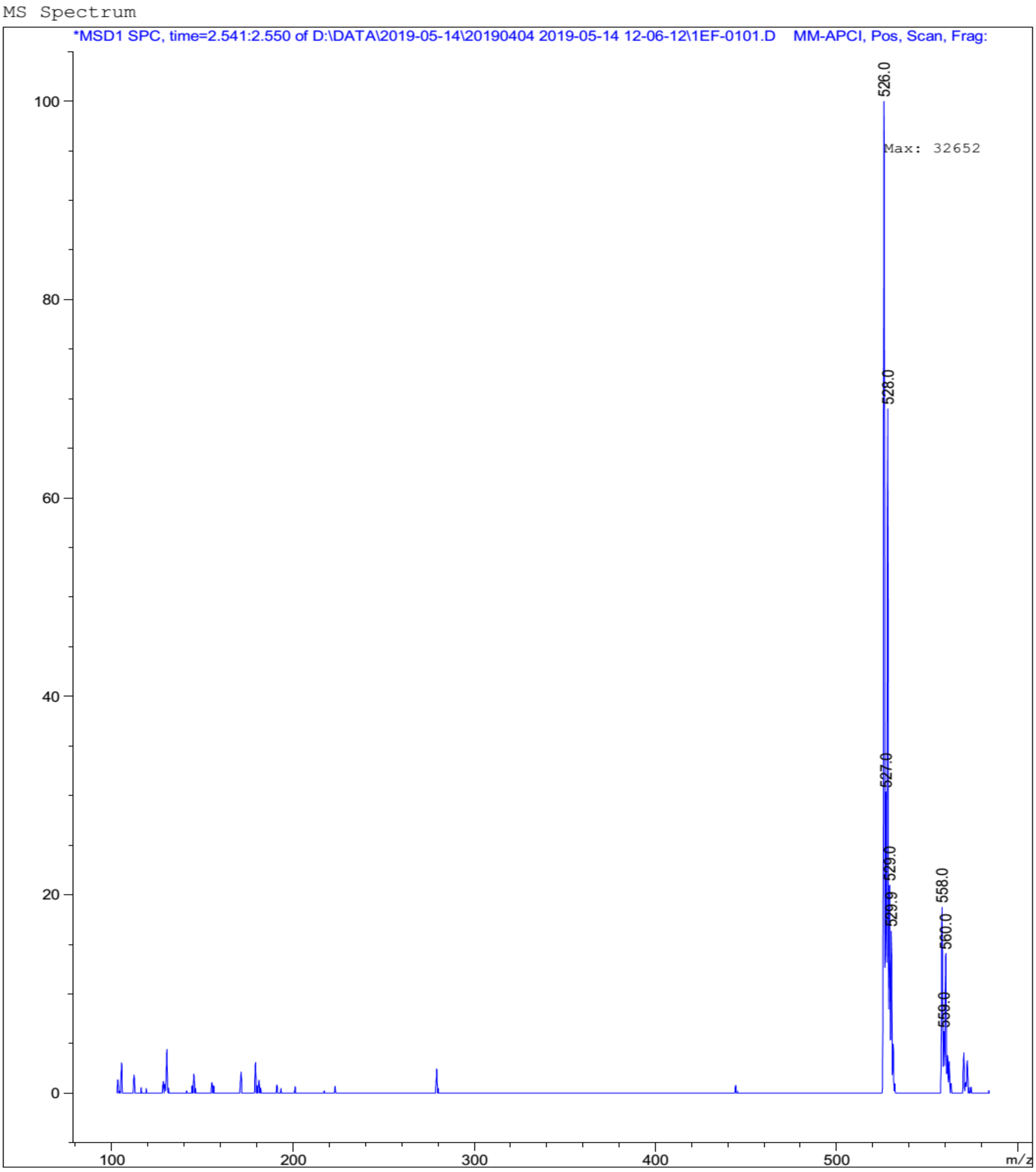

S98: HPLC-MS of **25**

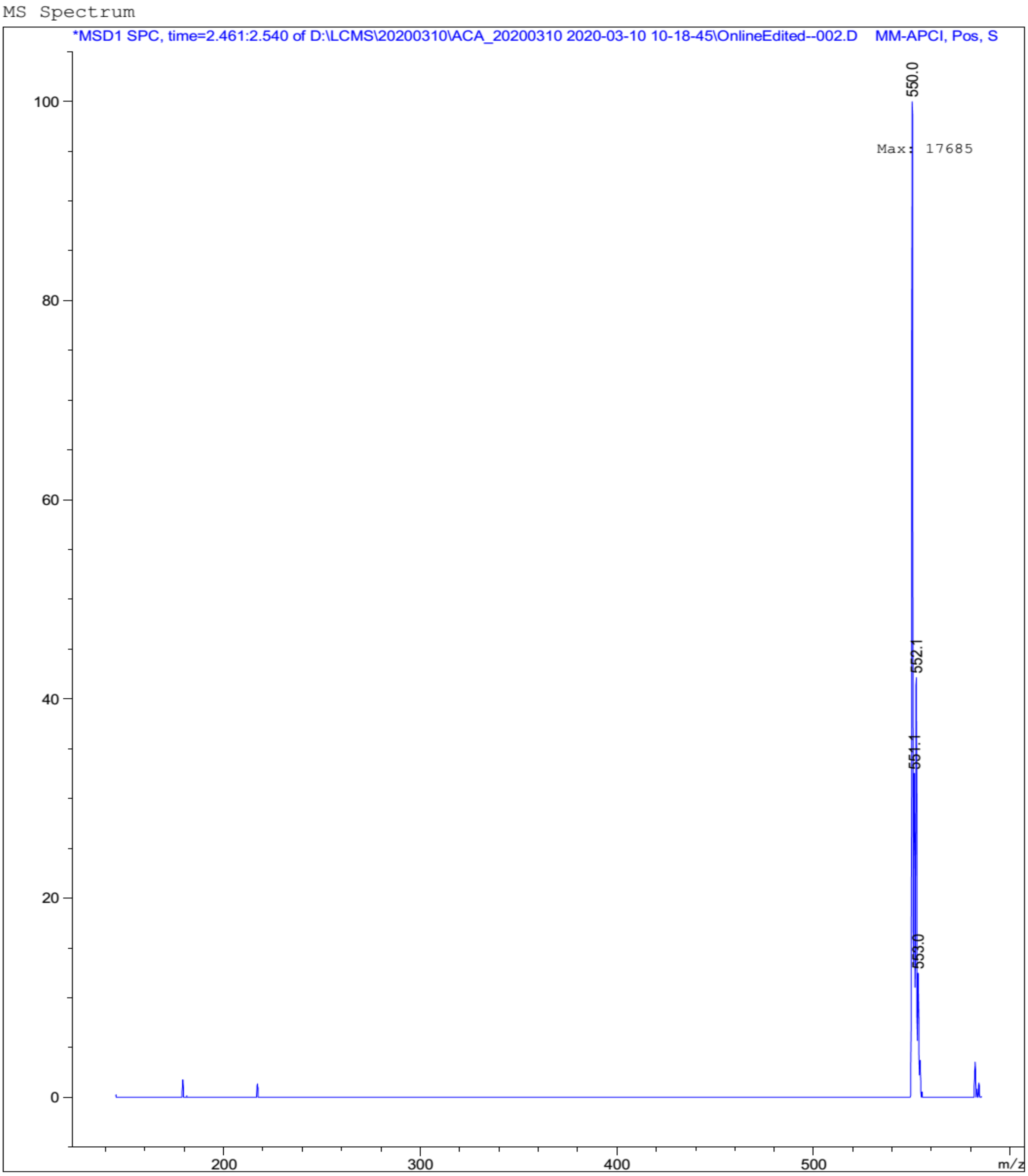

S99: HPLC-MS of **26**

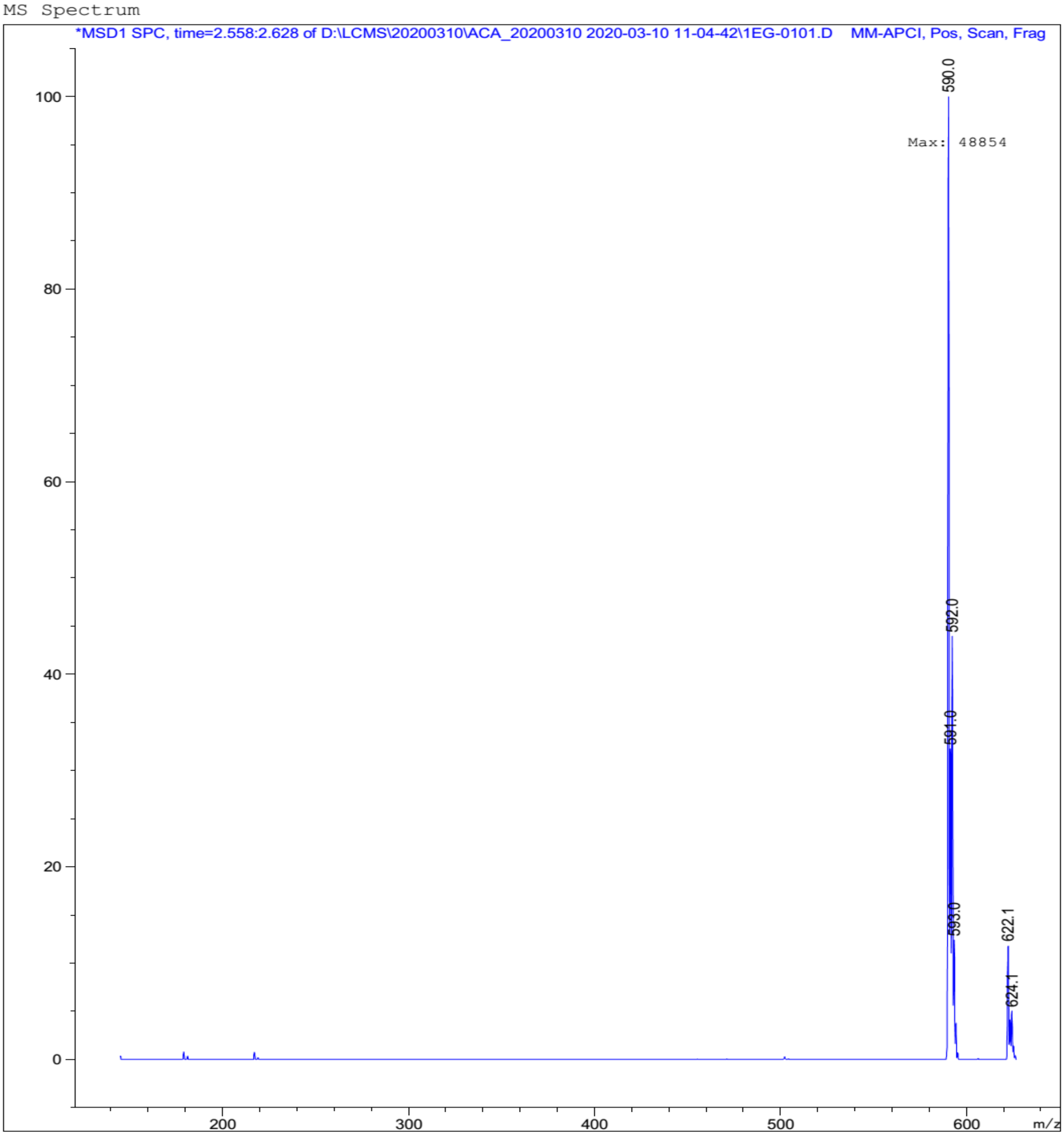

S100: HPLC-MS of **27**
